# Epidemiologic changes of infectious diseases in the post-SARS era in China, 2004–2018

**DOI:** 10.1186/s12889-023-16756-8

**Published:** 2023-11-06

**Authors:** Yizhe Luo, Binxiong Wu, Yameng Xu, Lele Ai, Heng Lv, Jiahong Wu, Weilong Tan

**Affiliations:** 1 Department of Epidemiology, School of Public Health, Nanjing Medical University, Nanjing, 211166 P.R. China; 2 Nanjing Bioengineering (Gene) Technology Center for Medicines, Nanjing, 210002 P.R. China; 3 Guizhou Medical University, Guiyang, Guizhou 550025 P.R. China

**Keywords:** Infectious diseases, Incidence, Trends, Seasons, China, Surveillance

## Abstract

**Objectives:**

To outline 44 major infectious diseases in the post-SARS (severe acute respiratory syndrome) in China and describe their long-term trends and changes by age, sex, epidemic season, and province.

**Background:**

After the outbreak of severe acute respiratory syndrome (SARS) in 2003, with the change of infectious disease prevention and control system and the improvement of residents’ quality of life, the incidence and mortality of infectious diseases have undergone major changes.

**Methods:**

The data of 44 major infectious diseases in China from 2004 to 2018 were obtained from the monthly analysis report of the China Information System for Disease Control and Prevention (CISDCP) and the Public Health Science Data Center. Joinpoint r regression models were used to examine trends in incidence and mortality for 44 major and important infectious diseases from 2004 to 2018.

**Results:**

From 2004 to 2018, 20,105, 500, 772 patients (10, 306, 546, 523 males and 9, 798, 954, 249 females) were diagnosed with 44 major infectious diseases. The overall incidence of 44 infectious diseases increased significantly from 294.6 per 100,000 people in 2004 to 479.1 per 100,000 people in 2010, with 7.9% APC (95% CI 5.2% -10.7%, *P* < 0.001), then slowed, and then increased to 561.2 per 100,000 people in 2018, with 1.5% APC (-0.1%—3.2%, *P* = 0.070). The overall mortality rose significantly, from 0.49 to 1.13 per 100,000 people between 2004 and 2011, with an APC increase of 11.6% (7.7% -15.6%, *P* < 0.001), and then remained stable until 2018. Among these, the prevalence of vaccine-preventable diseases and gastrointestinal & enteroviral diseases remained high and increased year by year. Patients with zoonotic diseases have the greatest risk of death, while patients with sexually transmitted and blood-borne diseases have the greatest number of deaths. Incidence rates vary considerably across geographic regions. Western China has a disproportionate burden of infectious diseases compared with eastern regions.

**Conclusions:**

After the event of SARS in 2003, infectious disease preventing and controlling model has undergone major changes in China, and certain achievements have been made in this field. Although overall morbidity and case fatality rates are still rising, they have leveled off. In reducing the disproportionate disease burden in the western region, expanding vaccination programs, preventing further increases in rates of sexually transmitted diseases, renewing efforts for emerging and persistent infectious diseases, and addressing seasonal and unpredictable outbreaks (such as the COVID-19 pandemic), there are still remain many challenges.

**Supplementary Information:**

The online version contains supplementary material available at 10.1186/s12889-023-16756-8.

## Introduction

Over the past few decades, China has undergone a rapid epidemiological transition with dramatic successful control in infectious diseases and life expectancy increased from 35 years in 1949 to 77.4 years in 2019 [1]. Thanks to the improvement of sanitary condition and the development of vaccines and drugs, the overall morbidity and mortality in China have steadily declined along with concerns on infectious diseases beginning to subside [2]. However, human health in the new era is still threatened by some infectious diseases [3].

In the past two decades, the epidemiology of infectious diseases has changed dramatically. Classic infectious diseases such as smallpox, plague, cholera, schistosomiasis, and kala-azar have disappeared or been decreasing. Influenza, dengue, Ebola, Zika and other emerging and re-emerging infectious diseases are still circulating globally [4]. The SARS epidemic in 2003 exposed the shortcomings of the global infectious disease prevention system [5]. The COVID-19 has further highlighted the necessity of infectious disease surveillance, prevention, and control. Since 2004, significant changes have taken place in the mode of infectious disease prevention and control in China. Electronic reporting initiated in 1985 was replaced by direct online reporting in 2004 (China Information System for Disease Control and Prevention (CISDCP)), realizing the transformation from non-coordinated prevention and control to joint prevention and control [6]. CISDCP realized the real-time and online reporting and monitoring of the case information of major infectious diseases in China. In addition, in order to comprehensively improve the level of infectious disease prevention and control, a mobile phone reporting application was launched in 2008 and integrated into the existing system till today. Anyway, it is virtually a long way for human beings to understand, prevent, and control infectious diseases [1].

Judging from the epidemic of infectious diseases in the past 20 years and its impact on the economy and society, we still need to pay attention to the monitoring and control of infectious diseases. However, there is little research on the changes in the pattern of infectious diseases in China after 2004. In order to describe the changing epidemiology in the post-SARS era and highlight potential challenges for major control strategies, we summarized the epidemic characteristics of main infectious diseases and explored their epidemic regularity for future control. To our knowledge, this is by far the largest epidemiological study on infectious diseases, which collects data from all over China and covers the largest population and the most extended period.

## Method

### Data collection

Data were obtained from China Information System for Disease Control and Prevention (CISDCP) (https://www.chinacdc.cn/) and the Public Health Science Data Center (https://www.phsciencedata.cn/Share/). The web-based system has been in operation at all administrative levels and regions since the SARS outbreak in 2003. In 1955, the national infectious disease surveillance system was officially launched. The number of major infectious diseases increased from 18 in 1955 to 25 in 1978. These diseases are divided into two classes, A and B. In 1989, China promulgated the first law on the prevention and treatment of infectious diseases and divided infectious diseases into three classes: A, B, and C. At present, there are 40 major infectious diseases in China (COVID-19 was officially announced as a Class B infectious disease in 2020). Considering that non-drug interventions and other measures during the coronavirus pandemic may break the original epidemic patterns of infectious diseases, especially respiratory (such as tuberculosis and influenza) and other common endemic infectious diseases, we did not include major infectious diseases in 2019 and beyond in our study.

### Classification

In order to make a more accurate statistical analysis of each common infectious disease, we reclassified major infectious diseases. We used hepatitis A, B, C, and E instead of simply saying viral hepatitis. The 40 major infectious diseases were reclassified into seven categories and 44 diseases. The seven categories include quarantinable diseases; vaccine-preventable diseases; gastrointestinal and enteroviral diseases; vector-borne diseases; zoonotic diseases; bacterial infections; sexually transmitted and blood-borne diseases. The main revision was the division of viral hepatitis into vaccine-preventable diseases (hepatitis A, B and D), zoonotic diseases (including hepatitis E) and sexually transmitted and blood-borne infections (hepatitis C) (see Table 1 and Supplementary classification criteria).

**Table 1:** Classification of 44 infectious diseases.

Disease classification
1. Quarantinable diseases
Haemorrhagic Fever
Cholera
Plague
2. Vaccine-preventable diseases
Mumps
Rubella
Measles
Seasonal Influenza
Hepatitis B
Hepatitis A
Pertussis
Hepatitis D
Diphtheria
Neonatal Tetanus
Poliomyelitis
3. Gastrointestinal or enterovirus borne diseases
Other Infectious diarrhea
Dysentery
Hand, Foot, and Mouth Disease
Acute Haemorrhagic Conjunctivitis
Typhoid/Paratyphoid
4. Vector-borne diseases
Malaria
Japanese Encephalitis
Typhus
Schistosomiasis
Kala-azar
Dengue
Filariasis
5. Zoonotic infections
Brucellosis
Hepatitis E
Rabies
Hydatid disease
Leptospirosis
Anthrax
H7N9
H5N1
H1N1
SARS
6. Bacterial infections
Tuberculosis
Scarlet Fever
Meningitis
Leprosy
7. Sexually transmitted and blood-borne infections
Gonorrhoea
Syphilis
HIV/AIDS
Hepatitis C

### Statistical analysis

We defined morbidity (per 100,000 people) as annual cases divided by population size; case fatality rate (per 1,000) as annual deaths divided by annual cases; total mortality (per 100,000) calculated as annual deaths divided by population size. Descriptive analysis was used to show morbidity, case-fatality, and mortality for 44 infectious diseases from 2004 to 2018. Heatmaps were used to show the trends and distribution characteristics of the incidence, case fatality rate and death toll over time for each infectious disease by category. Maps were used to analyze the geographic distribution characteristics of different infectious diseases. We also analyzed major infectious diseases by age group and the seasonal variation of each disease by month.

We used joinpoint regression models (see Supplementary method) to examine incidence trends from 2004 to 2018. The cut-off points of jointpoint regression models were selected or explained according to the policy. The *Z* test was used to assess whether the annual percentage change (APC) was significantly different from zero. We described trends in 44 major infectious diseases over 15 years as increasing (if *p* < 0.05), decreasing (if *p* < 0.05), and stabilizing (if *p* ≥ 0 05). We used IBM SPSS Statistics (version 26), R (version 4.1.0), and Joinpoint (version 4.9.0.0) for data analysis.

## Results

### General trends in infectious diseases

**Fig. 1:**
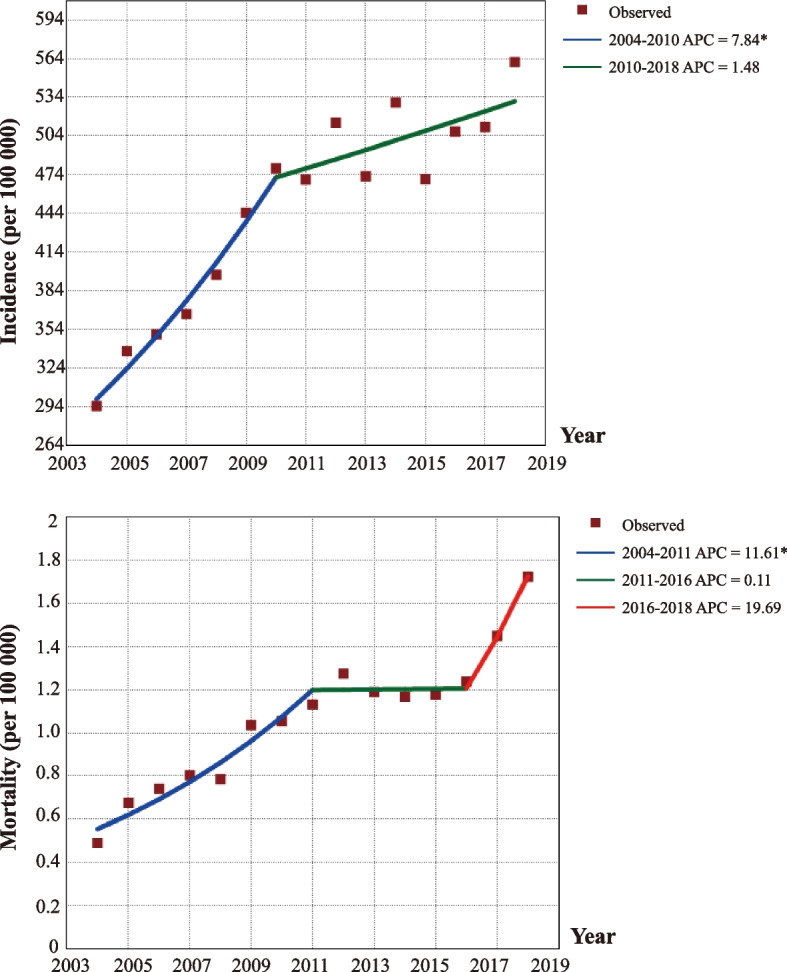
Trends in morbidity and mortality for 44 major infectious diseases, 2004–2018. *Statistically significant trends. APC = Annual Percentage Change

From 2004 to 2018, 20, 105, 500, 772 cases (10, 306, 546, 523 males and 9, 798, 954, 249 females) were diagnosed with 44 infectious diseases in China. The joinpoint regression model identified a turning point in 2010 and two distinct linear trends in overall incidence (Fig. 1). The overall incidence of 44 infectious diseases increased significantly from 294.6 per 100,000 people in 2004 to 479.1 per 100,000 people in 2010, with 7.9% APC (95% confidence interval 5.2% to 10.7%, *P* < 0.001), then slowed, and then increased to 561.2 per 100,000 people in 2018, with 1.5% APC (-0.1% to 3.2%, *P* = 0.070). The overall mortality rose significantly, from 0.49 to 1.13 per 100,000 people between 2004 and 2011, with an APC increase of 11.6% (7.7% to 15.6%, *P* < 0.001), and then remained stable until 2018 (*P* = 0.976, 2011–2016; *P* = 0.152, 2016–2018, Supplementary Table 1). The total number of deaths from infectious diseases also rose, from 7,135 in 2004 to 42,729 in 2018, with an overall relative increase of 498.86% (Supplementary Table 2).

### Trends in infectious disease categories

**Fig. 2:**
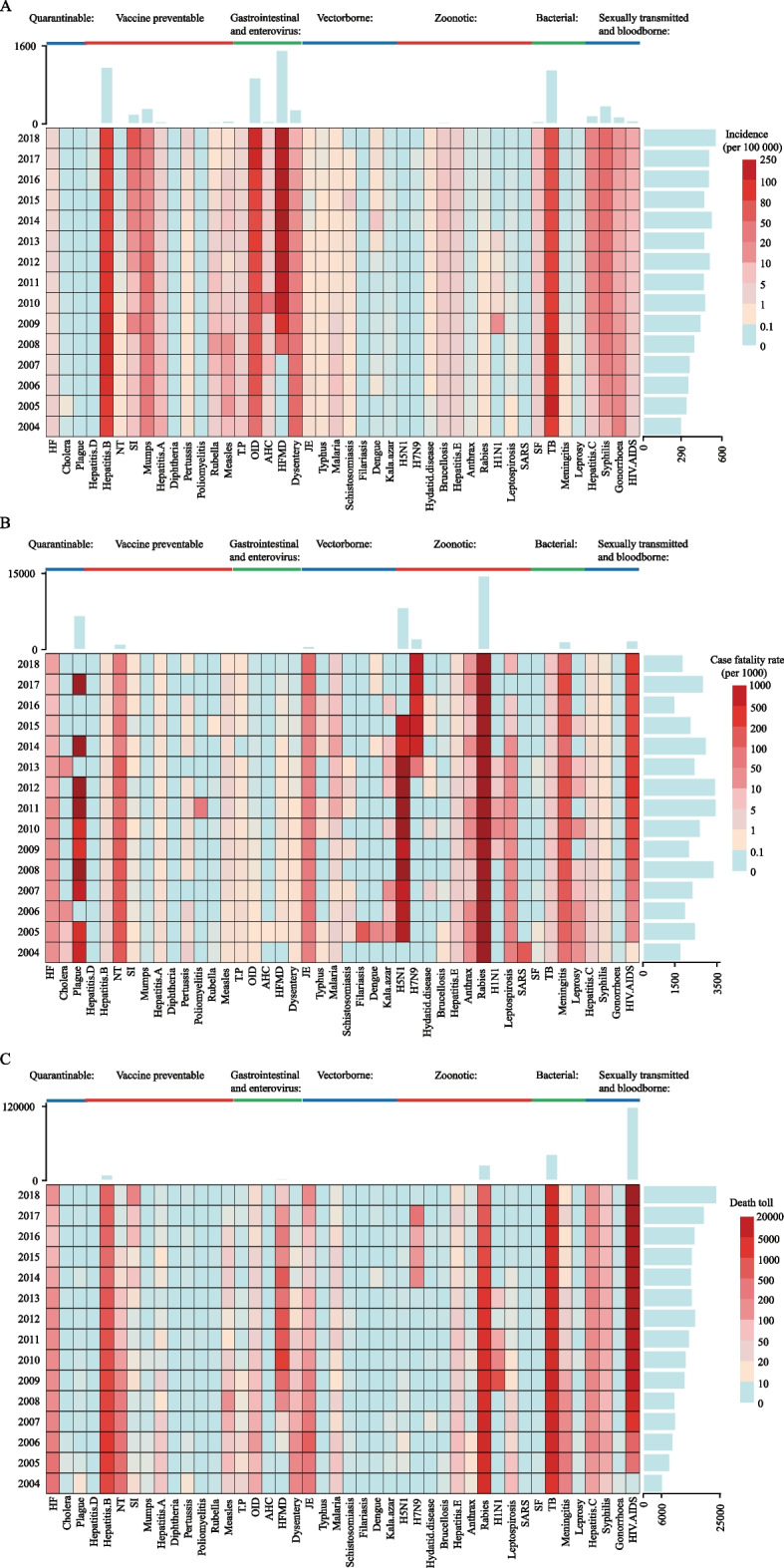
Incidence and case-fatality and death toll trends for 44 major infectious diseases, 2004–2018. 15-year trends in (**A**) incidence (per 100 000) (**B**) case fatality (per 1000) and (**C**)) death toll of 44 major infectious diseases by year from 2004 to 2018. The Y-axis (left) represents time, from 2004 to 2018; the bars on the Y-axis (right) represent the sum of the corresponding parameters for each year for 44 major infectious diseases. The X-axis represents 44 major infectious diseases, with each vertical row representing an infectious disease; the bars on the X-axis (above the x axis) represent the sum of the corresponding parameters for each infectious disease over a 15-year period (2004–2018). The different colors represent the incidence, severity and number of deaths. The light blue box represents that the incidence of the infectious disease, and the case fatality rate or number of deaths are zero

**Fig. 3:**
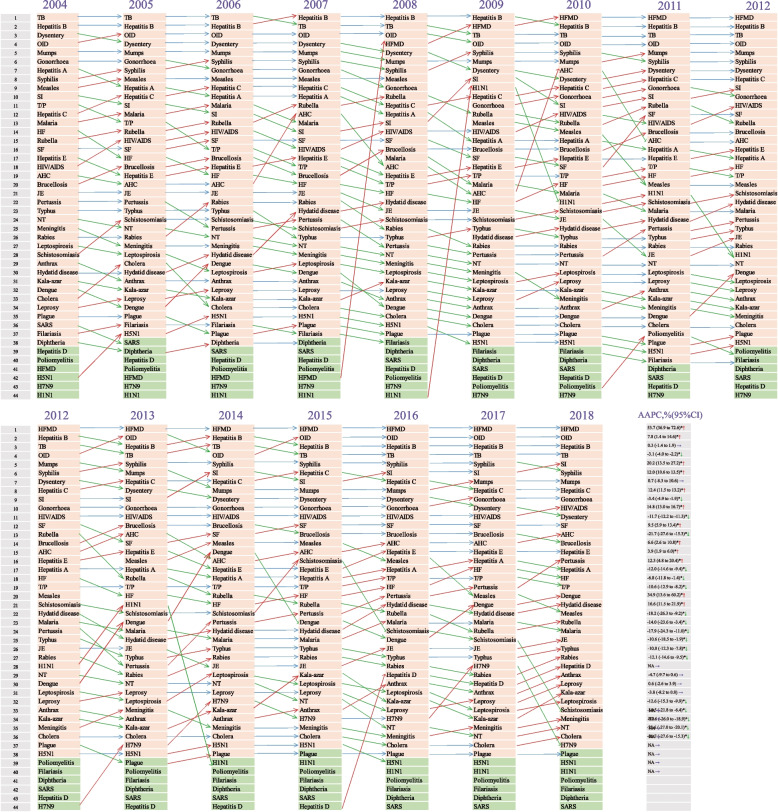
Incidence ranking of 44 major infectious diseases, 2004–2018. The incidence rates of 44 major infectious diseases were ranked by year from 2004 to 2018. The annual percentage change (APC) in the incidence of each infectious disease with 95% confidence intervals (95% CI) is listed on the far right. In the APC column, the upward arrow (red) represents the overall upward trend, the downward arrow (green) represents the downward trend, and the right arrow (purple) represents the stable trend of the incidence of each infectious disease from 2004 to 2018. Orange means no cases of infectious disease

Among the seven categories, vaccine-preventable and gastrointestinal and enteroviral diseases accounted for 69.52% of all reported cases of major infectious diseases (Fig. 2). Figure 2 and Supplementary Table 3 show trends in morbidity, case fatality, and death toll for 44 infectious diseases over 15 years by seven categories, as well as relative rankings (Fig. 3, Supplementary Figs. 1–3 and Supplementary Table 4). In general, the prevalence of vaccine-preventable diseases, gastrointestinal and enteroviral diseases, bacterial infections, and sexually transmitted and blood-borne diseases remained high and was increasing year by year. Zoonotic diseases had the greatest risk of death, while sexually transmitted and blood-borne diseases had significantly increased deaths. For a detailed discussion of trends for 44 infectious diseases, see Supplementary Discussion.

### Variations by age, gender and season

**Fig. 4:**
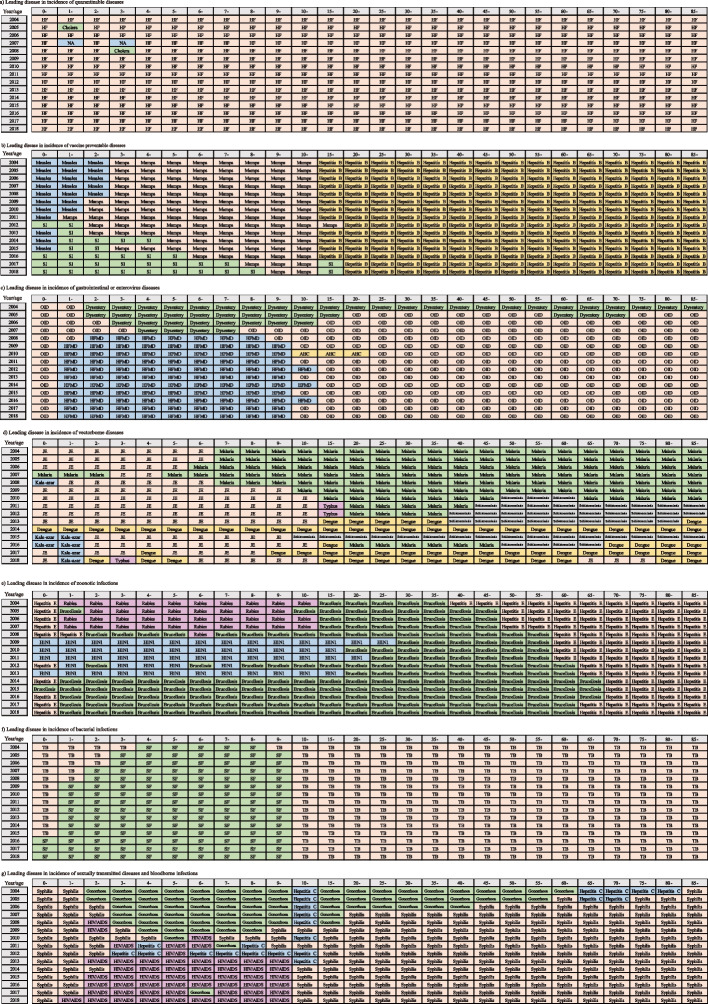
Infectious diseases with the highest incidence by age, 2004–2018. The infectious diseases that appear in the box represent the major infectious diseases with the highest incidence in the corresponding age group in the current year

Incidence of infectious diseases varied by age (Fig. 4 and Supplementary Table 5). Among the three quarantinable infectious diseases of haemorrhagic fever, cholera, and plague, hemorrhagic fever was the dominant disease in a year in all age groups. Among vaccine-preventable diseases, the dominant one was measles among children under 2 years old before 2011, and it was surpassed by seasonal influenza since 2012. Main diseases in people aged 3–10 and 15–85 were mumps and hepatitis B, respectively. Among gastrointestinal diseases and enteroviral diseases, OID was the most common infectious disease in children aged 0 years and people over 10 years old, and HFMD was the main disease in children aged 1–9 years. For vector-borne diseases, Japanese encephalitis was the leading diagnosis in children aged less than 6 years, whereas it was surpassed by malaria, dengue, and schistosomiasis in people older than 6 years. Dengue fever and schistosomiasis were the most common infectious disease since 2014. Among zoonotic infections, brucellosis was the most common infection in the 1–55 age group, apart from the 2009–2013 outbreak of influenza A (H1N1). Hepatitis E was predominant in those aged 0 years and older than 55 years. For bacterial infections diseases, scarlet fever dominated among children aged 1 to 9 years, while tuberculosis was the leading disease in people aged 0 years and older than 9 years. Syphilis, gonorrhea, HIV/AIDS were the most common diseases among all age groups. HIV/AIDS has increased since 2010, especially among children aged 2–9 years.

Generally, significant associations were observed between different age groups and infectious diseases (Supplementary Tables 6–8). The most common infectious diseases in early childhood included vector-borne diseases, gastrointestinal and enterovirus diseases. Diseases that primarily affected adults included zoonotic diseases and sexually transmitted and bloodborne diseases. In terms of gender differences, the number of cases and incidence of almost all infectious diseases, especially hepatitis C, syphilis, gonorrhoea and HIV/AIDS, were higher in males than in females within the 15 years (*P* < 0.05), except for cholera, typhus, dengue, H5N1, hydatid disease, and syphilis (see Supplementary Figs. 4 and 5).

Over the 15 years, seasonal variation was observed in 34 infectious diseases (see Supplementary Fig. 6 and Table 3), especially haemorrhagic fever (October-December), diphtheria (May–July), H7N9 and H5N1 influenza (January) and dengue (September–October). Almost all infectious diseases peaked in summer and autumn.

### Regional difference

**Fig. 5:**
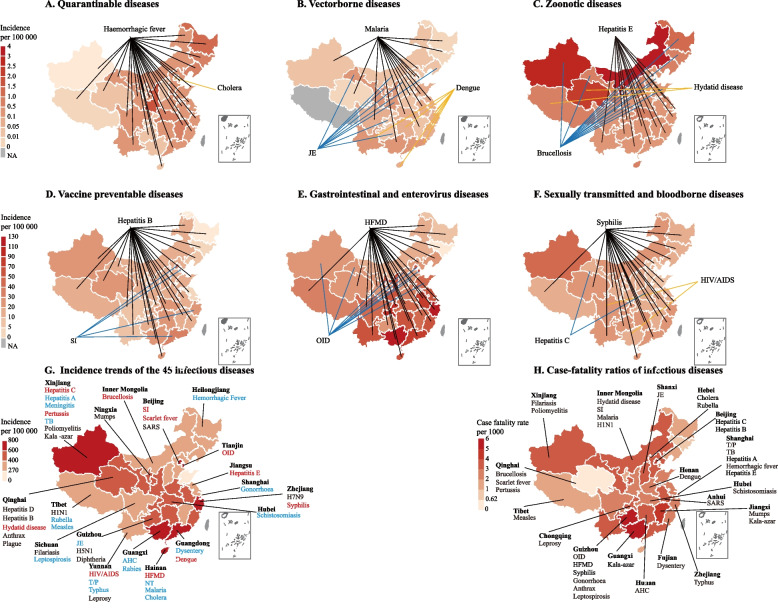
Geographical distribution of 44 major infectious diseases in 2018 and trends in morbidity and mortality of infectious diseases in different geographic regions in China from 2004 to 2018. The incidence rates of the 44 major infectious diseases vary widely by category. **A-F** Tuberculosis is predominant in all provinces, with no regional differences, so bacterial infections diseases are not shown in the figure. Lines indicate major infectious diseases in each province. **G** Incidence trends of 44 infectious diseases in different provinces in China. The infectious diseases with the highest average incidence were distributed in the designated provinces. Infectious diseases with an increasing incidence from 2004 to 2018 are shown in red; those with a decreasing incidence are shown in blue; and those that have remained stable are shown in black. **H** Case fatality rates of infectious diseases in different provinces in China. The case fatality rates of rabies, H7N9, H5N1 and other highly lethal diseases are not shown in the figure

From 2004 to 2018, the overall annual morbidity and case fatality rates of different infectious diseases varied widely among different geographic regions. The three provinces with the highest total annual incidence of 44 infectious diseases were Guangxi (772.20 cases per 100, 000), Xinjiang (765.51 cases per 100, 000) and Beijing (721.98 cases per 100, 000). With rabies, HIV and other highly lethal diseases excluded, the three provinces with the highest total annual fatality rate were Guizhou (215.62 deaths per 1000 cases), Guangxi (212.54 deaths per 1000 cases) and Beijing (168.97 deaths per 1000 cases). Among the 44 infectious diseases, the incidence of 12 infectious diseases increased significantly and 18 declined significantly (Fig. 5).

In 2018, region-specific patterns were showed in incidence of infectious diseases. For vaccine-preventable diseases, hepatitis B was the most common disease in 27 provinces in eastern and western China. Qinghai (155.54 cases per 100, 000), Hainan (152.29 cases per 100, 000) and Xinjiang (149.75 cases per 100, 000) were the three provinces with the highest overall yearly incidence. Seasonal influenza was mainly prevalent in Beijing, Zhejiang and Guangdong. OID and HFMD were the most common gastrointestinal and enteroviral diseases. HFMD remained the leading disease in most provinces, and OID was mostly prevalent in Beijing and Tianjin. Vector-borne diseases were relatively rare, but still dominated in Ningxia and Gansu. Among zoonotic diseases, hepatitis E was mostly prevalent in southern China, brucellosis was dominant in most northern region, and hydatid disease remained the leading zoonotic disease in western regions, such as Tibet, Qinghai, and Ningxia. Among sexually transmitted and blood-borne infections, hepatitis C predominated in Qinghai and Xinjiang. Syphilis was mostly prevalent in 28 provinces, with the highest incidence in Xinjiang (96.54 cases per 100, 000), and AIDS/HIV was highest in Sichuan and Guangxi. Overall, western China was continually burdened with disproportionate incidence of diseases. The incidence of zoonotic diseases, vaccine-preventable diseases and bacterial infections, especially in Qinghai, Inner Mongolia, Ningxia and other western provinces, were higher than in eastern regions.

## Discussion

### Epidemiological changes

Although the overall incidence of infectious diseases in China showed an increasing trend from 2004 to 2018, fortunately, the trend levelled off after 2010. Overall mortality from infectious diseases has risen significantly, with HIV/AIDS replacing tuberculosis and rabies as the leading cause of death from infectious diseases since 2008. The incidence of most vaccine-preventable diseases remained low, but seasonal changes in seasonal influenza, mumps, pertussis, and rubella suggested that these diseases, especially pertussis and seasonal influenza (incidence increased rapidly over the 15 years), should remain a public health priority. Among quarantinable diseases, classic infectious diseases such as cholera and plague almost disappeared. Except dengue and scarlet fever, the incidence of vector-borne diseases and bacterial infections diseases showed a decreasing trend. Over the 15 years, dengue and scarlet fever cases increased 20.7-fold and 4.2-fold, respectively. It is worth noting that, through regional and inter-departmental cooperation, such as the Five Middle Provinces and Three Southern Provinces Malaria Joint Control and Prevention Programme, and the improvement of the reference laboratory system for malaria diagnosis, and the implementation of the ‘1–3-7’ malaria surveillance and response strategy, China was certified by WHO in 2021 as the 40th malaria free country. However, continued increase in imported cases is a current challenge [7–9]. In contrast to the sharp decline in indigenous cases, imported malaria cases showed an explosive growth, with the percentage rising from 16.2% in 2004 to 99.9% in 2017 [10]. It is also worth noting that, due to the widespread distribution of *Anopheles sinensis* in China, the possibility of malaria comeback still exists, especially *P*. vivax malaria. Therefore, China still needs to remain vigilant against the resurgence of malaria. It is necessary to maintain a surveillance and response system and focus on hot spots and risk groups, with timely detection of imported cases, rapid reporting, and prevention of continued transmission. In addition, epidemiological and entomological surveillance is also necessary [10]. Increased incidences of hepatitis C, syphilis, and in particular HIV/AIDS, suggested that sexually transmitted diseases became an important new focus. Other gastrointestinal or enterovirus borne diseases still had relatively high prevalence despite continuous declines in dysentery and typhoid/paratyphoid. A clear geographic distribution of infectious diseases was observed, with bacterial infections and zoonotic diseases occurring more frequently and carrying a disproportionate health burden in western China.

Morbidity of Hepatitis B and mumps were dominant in vaccine-preventable diseases. Although the incidence of hepatitis B has declined since 2007, it remained one of the highest incidence infectious diseases in China for 15 years. With the implementation of the national immunization program and the improvement of sanitary conditions, the incidence of hepatitis A and B in Chinese adolescents and children has decreased significantly, while that in Chinese adults has increased [11, 12]. Thanks to the expanded immunization program, the incidence of mumps began to decline in 2012, but has increased since 2015, and it is now the most common infection among children aged 3–10 years [13]. Notably, diseases such as pertussis and seasonal influenza have experienced unexplained resurgence worldwide over the past decade, raising concerns about vaccine effectiveness, coverage, and more [14].

Tuberculosis is one of the top ten causes of death globally, with its death toll second only to HIV/AIDS in China. From 2004 to 2018, the tuberculosis incidence ranking dropped from 1st to 4th, with a significant decrease from 74.64 to 59.27 cases per 100, 000 (APC = -3.1%, *P* < 0.001). The national tuberculosis control programme, which began in 1991, was likely to be the primary cause of the decline [15]. At present, drug-resistant tuberculosis is a major threat to the control and elimination of tuberculosis in China [16]. In 2017, approximately 14% of tuberculosis patients worldwide died from MDR-TB [17].

Incidence of scarlet fever has been increasing globally. Outbreaks have occurred in Vietnam [18], Hong Kong [19], and the United Kingdom one after another since 2008 [20]. The relaxation of family planning policies and the increasing susceptible population have led to the resurgence of scarlet fever [21, 22]. This prompted public health departments to establish school-based surveillance and emergency response systems. Similar measures can help combat hand-foot-mouth disease, infectious diarrhea and other susceptible childhood diseases, but their effectiveness was unclear [23]. Scarlet fever was predominant among children aged 1 to 9 years in western and northern China, while tuberculosis was the main disease among people aged 0 and older than 9 years in southern China. The regional heterogeneity of morbidity indicated that different regions should carry out the prevention and control of infectious diseases according to their own epidemiological characteristics.

During the studied period, HIV/AIDS incidence and deaths in China continued to increase, especially in males, and the infection in younger population showed an upward trend [11]. High-risk sexual behavior appeared to be one of the reasons for the increasing incidence of HIV/AIDS [24]. As of the end of September 2018, 850,000 people living with HIV infection and 262,000 deaths were reported, and sexual behavior was the main route of transmission. In 2017, heterosexual transmission accounted for 69.6% of reported infections, and male homosexual transmission accounted for 25.5% [25]. Insufficient sex education, pursuit of casual sex, and social media hype seemed to be the factors contributing to the increasing incidence. Although the government has launched response policies, a series of measures are urgently needed for policy implementation, including publicity campaigns and more attention from the education sector, family and school-based sex education, and purification of the network environment [24].

Benefited from public health interventions and mass vaccination programmes, the overall morbidity and mortality from infectious diseases in China have declined sharply in recent decades. However, after the SARS outbreak in 2003, the overall incidence in China showed a gradual upward trend, which we confirmed in our study of 44 major infectious diseases [1]. Fortunately, this trend changed after 2010. Several factors may explain this growing trend. First, the timeliness of reporting has been improved. Many emerging infectious diseases have abrupt onset, high fatality rate, difficult early detection and diagnosis, and lack of targeted prevention and control measures. Thanks to the improvement of the monitoring system and the medical diagnosis, more occult cases have been discovered. In addition, the development of health care, such as the widespread use of polymerase chain reaction (PCR), has made the diagnosis of diseases more convenient. In fact, we often see a significant increase in the diagnosis of a disease when some fast, convenient and highly specific techniques are developed and used in medical applications [26]. Second, large-scale population movement increases the difficulty of prevention and control, and measures such as vaccination are difficult to implement. Frequent international business exchanges increase the risk of cross-border transmission of infectious diseases and unsafe sex increases the risk of sexually transmitted diseases. Third, the environment and the existing production and lifestyle promote the spread of infectious diseases. The urban and rural environmental sanitation in some areas is in a worrying situation, and the traditional production and lifestyle make zoonotic diseases continue to occur. Overall, current prevention and control work in China is facing dual pressures from traditional and emerging infectious diseases. Prominent problems include backward infrastructure, weak monitoring and testing capabilities, insufficient funding, and serious shortage of professionals. Implementation of relevant provisions of the Law on the Prevention and Control of Infectious Diseases should be further strengthened in order to build strong foundations and make up for shortcomings. More attention should be paid to talent team building and professional capabilities improvement, along with the increasing investment in hardware such as equipment and facilities.

### Recommendation for health promotion

Over the past few decades, China has adopted targeted strategies to prevent the spread of infectious diseases. Some measures have been proven to be effective and can provide insights and important lessons for other developing countries. First of all, improving the system of laws, regulations and policies. Legalize, institutionalize, standardize and standardize diseases prevention and control work. Secondly, it is vital to build a tight public health monitoring network. The national disease control information system of the national health security information project has been put into operation, and the world's largest, horizontal and vertical disease and health risk factor monitoring network has been built, and the monitoring network of key endemic diseases and drinking water quality covers all townships and towns in China, and the monitoring and intervention system of common diseases and health hazards of students who are mainly regarded as the nearest is becoming more and more perfect. For example, improved sanitation and water supply facilities, improved blood collection safety and large-scale vector control can add to the successful prevention of infectious diseases.

### Strength and limitation

Some limitations of our study should be noted. First, the effectiveness of monitoring systems and the accuracy of diagnosis based on data from reporting systems may be overestimated. Second, incidence may be underestimated due to self-selection bias, as people with a particular infectious disease are more likely to avoid screening than those without the infection. In addition, potential biases may affect morbidity and mortality reporting due to differences in the level of medical care in different regions, the level of hospital diagnosis, or inclusion/exclusion criteria.

## Conclusion

In conclusion, we described the epidemiological characteristics and changing trends of 44 infectious diseases during the longest post-SARS period (2004–2018) studied in China. While the overall incidence of infectious diseases has trended upward over the 15-year period, the trend has changed after 2010 (the rate of increase has slowed). From 2004 to 2018, 12 of the 44 infectious diseases showed an upward trend and 18 showed a downward trend. In addition, morbidity and mortality in the post-SARS era varied widely. HFMD has replaced tuberculosis and hepatitis B as the most common infectious disease; HIV/AIDS has replaced rabies as the most common cause of death. Among them, HFMD, OID and hepatitis B had the highest annual incidence rates; HFMD, dengue and seasonal influenza increased the most. Moreover, the disease burden in the western region persisted and far exceeded that in the eastern region. Therefore, public health departments should focus on preschool children, the elderly, male, and people living in the western region, and formulate customized and precise prevention and control strategies based on local epidemics in the future. In addition, since the implementation of the immunization program, China has achieved great success in controlling vaccine-preventable diseases, and the incidence of most diseases decreased sharply after being included in the Expanded Program on Immunization (EPI), while the seasonal influenza, which was not included in the EPI, showed a persistent incidence. It is worth noting that pertussis is making a comeback, which may expose problems such as insufficient vaccine immunization doses, insufficient duration of vaccine protection, and differences in vaccination strategies.

### Supplementary Information

**Additional file 1: Supplementary method.** Joinpoint regression. **Supplementary discussion.** **Table 1.** The incidence of seven categories, total incidence and mortality of infectious diseases from 2004 to 2018, per 100,000. **Table 2.** Fifteen-year trends in incidence, number of cases, mortality and deaths of 44 notifiable infectious diseases in China, 2004-2018. **Table 3.** Changes in number of cases, incidence (per 100 000), number of deaths, and case-fatality ratios (per 1000) for 44 notifiable infectious diseases in China, 2004-2018. **Table 4.** The annual percentage changes (APC) and the joinpoint year range for 44 current notifiable infectious diseases in China from 2004 to 2018. **Table 5.** Incidence (per 100 000) of 44 infectious diseases stratified by age groups (years). **Table 6.** Incidence (per 100 000) of 44 infectious diseases stratified by gender. **Table** **7.** Incidence (per 100 000) of 44 infectious diseases stratified by gender and transmission routes. **Table** **8.** Incidence (per 100 000) of 44 infectious diseases stratified by gender and transmission routes. **Fig.** **S1.** The annual percentage changes (APC) and turning point in the trend from 2004 to 2018 for total and seven categories of infectious diseases using the joinpoint regression models. **Fig.** **S2.** Ranks of mortality or number of deaths for 44 notifiable infectious diseases during the past 15-years from 2004 to 2018. **Fig.** **S3.** The trends of incidence of each infectious disease in both genders and its joinpoint(s) during the past 15-years from 2004 to 2018. **Fig.** **S3-1.** The trends of incidence of Haemorrhagic Fever in both genders and its joinpoint(s) during the past 15-years from 2004 to 2018 (* represented the statistical significant trends. Legends were the same to Fig S1). **Fig.** **S3-2.** The trends of incidence of Cholera in both genders and its joinpoint(s) during the past 15-years from 2004 to 2018 (* represented the statistical significant trends. Legends were the same to Fig S1). **Fig.** **S3-3.** The trends of incidence of Hepatitis B in both genders and its joinpoint(s) during the past 15-years from 2004 to 2018 (* represented the statistical significant trends. Legends were the same to Fig S1). **Fig.** **S3-4.** The trends of incidence of NT in both genders and its joinpoint(s) during the past 15-years from 2004 to 2018 (* represented the statistical significant trends. Legends were the same to Fig S1). **Fig.** **S3-5.** The trends of incidence of SI in both genders and its joinpoint(s) during the past 15-years from 2004 to 2018 (* represented the statistical significant trends. Legends were the same to Fig S1). **Fig.** **S3-6.** The trends of incidence of Mumps in both genders and its joinpoint(s) during the past 15-years from 2004 to 2018 (* represented the statistical significant trends. Legends were the same to Fig S1). **Fig.** **S3-7.** The trends of incidence of Hepatitis A in both genders and its joinpoint(s) during the past 15-years from 2004 to 2018 (* represented the statistical significant trends. Legends were the same to Fig S1). **Fig.** **S3-8.** The trends of incidence of Pertussis in both genders and its joinpoint(s) during the past 15-years from 2004 to 2018 (* represented the statistical significant trends. Legends were the same to Fig S1). **Fig.** **S3-9.** The trends of incidence of Rubella in both genders and its joinpoint(s) during the past 15-years from 2004 to 2018 (* represented the statistical significant trends. Legends were the same to Fig S1). **Fig.** **S3-10.** The trends of incidence of Measles in both genders and its joinpoint(s) during the past 15-years from 2004 to 2018 (* represented the statistical significant trends. Legends were the same to Fig S1). **Fig.** **S3-11.** The trends of incidence of T/P in both genders and its joinpoint(s) during the past 15-years from 2004 to 2018 (* represented the statistical significant trends. Legends were the same to Fig S1). **Fig.** **S3-12.** The trends of incidence of OID in both genders and its joinpoint(s) during the past 15-years from 2004 to 2018 (* represented the statistical significant trends. Legends were the same to Fig S1). **Fig.** **S3-13.** The trends of incidence of AHC in both genders and its joinpoint(s) during the past 15-years from 2004 to 2018 (* represented the statistical significant trends. Legends were the same to Fig S1). **Fig.** **S3-14.** The trends of incidence of Dysentery in both genders and its joinpoint(s) during the past 15-years from 2004 to 2018 (* represented the statistical significant trends. Legends were the same to Fig S1). **Fig.** **S3-15.** The trends of incidence of JE in both genders and its joinpoint(s) during the past 15-years from 2004 to 2018 (* represented the statistical significant trends. Legends were the same to Fig S1). **Fig.** **S3-16.** The trends of incidence of Typhus in both genders and its joinpoint(s) during the past 15-years from 2004 to 2018 (* represented the statistical significant trends. Legends were the same to Fig S1). **Fig.** **S3-17.** The trends of incidence of Malaria in both genders and its joinpoint(s) during the past 15-years from 2004 to 2018 (* represented the statistical significant trends. Legends were the same to Fig S1). **Fig.** **S3-18.** The trends of incidence of Schistosomiasis in both genders and its joinpoint(s) during the past 15-years from 2004 to 2018 (* represented the statistical significant trends. Legends were the same to Fig S1). **Fig.** **S3-19.** The trends of incidence of Dengue in both genders and its joinpoint(s) during the past 15-years from 2004 to 2018 (* represented the statistical significant trends. Legends were the same to Fig S1). **Fig.** **S3-20.** The trends of incidence of Kala-azar in both genders and its joinpoint(s) during the past 15-years from 2004 to 2018 (* represented the statistical significant trends. Legends were the same to Fig S1). **Fig.** **S3-21.** The trends of incidence of Hydatid disease in both genders and its joinpoint(s) during the past 15-years from 2004 to 2018 (* represented the statistical significant trends. Legends were the same to Fig S1). **Fig.** **S3-22.** The trends of incidence of Brucellosis in both genders and its joinpoint(s) during the past 15-years from 2004 to 2018 (* represented the statistical significant trends. Legends were the same to Fig S1). **Fig.** **S3-23.** The trends of incidence of Hepatitis E in both genders and its joinpoint(s) during the past 15-years from 2004 to 2018 (* represented the statistical significant trends. Legends were the same to Fig S1). **Fig.** **S3-24.** The trends of incidence of Anthrax in both genders and its joinpoint(s) during the past 15-years from 2004 to 2018 (* represented the statistical significant trends. Legends were the same to Fig S1). **Fig.** **S3-25.** The trends of incidence of Rabies in both genders and its joinpoint(s) during the past 15-years from 2004 to 2018 (* represented the statistical significant trends. Legends were the same to Fig S1). **Fig.** **S3-26.** The trends of incidence of Leptospirosis in both genders and its joinpoint(s) during the past 15-years from 2004 to 2018 (* represented the statistical significant trends. Legends were the same to Fig S1). **Fig.** **S3-27.** The trends of incidence of Scarlet fever in both genders and its joinpoint(s) during the past 15-years from 2004 to 2018 (* represented the statistical significant trends. Legends were the same to Fig S1). **Fig.** **S3-28.** The trends of incidence of Tuberculosis in both genders and its joinpoint(s) during the past 15-years from 2004 to 2018 (* represented the statistical significant trends. Legends were the same to Fig S1). **Fig.** **S3-29.** The trends of incidence of Meningococcal meningitis in both genders and its joinpoint(s) during the past 15-years from 2004 to 2018 (* represented the statistical significant trends. Legends were the same to Fig S1). **Fig.** **S3-30.** The trends of incidence of Leprosy in both genders and its joinpoint(s) during the past 15-years from 2004 to 2018 (* represented the statistical significant trends. Legends were the same to Fig S1). **Fig.** **S3-31.** The trends of incidence of Hepatitis C in both genders and its joinpoint(s) during the past 15-years from 2004 to 2018 (* represented the statistical significant trends. Legends were the same to Fig S1). **Fig.** **S3-32.** The trends of incidence of Syphilis in both genders and its joinpoint(s) during the past 15-years from 2004 to 2018 (* represented the statistical significant trends. Legends were the same to Fig S1). **Fig.** **S3-33.** The trends of incidence of Gonorrhoea in both genders and its joinpoint(s) during the past 15-years from 2004 to 2018 (* represented the statistical significant trends. Legends were the same to Fig S1). **Fig.** **S3-34.** The trends of incidence of HIV/AIDS in both genders and its joinpoint(s) during the past 15-years from 2004 to 2018 (* represented the statistical significant trends. Legends were the same to Fig S1). **Fig.** **S4.** The comparison of incidence of 44 current notifiable infectious diseases in boys or males and girls or females from 2004 to 2018 (using the chi-square test). **Fig.** **S5.** Trends in age incidence rates for each infectious disease in females and males, from 2004 to 2018. **Fig.** **S5-1.** Trends in age incidence rates for Haemorrhagic Fever, females and males, 2004-2018. **Fig.** **S5-2.** Trends in age incidence rates for Cholera, females and males, 2004-2018 (Legends were the same to Fig S5-1). **Fig.** **S5-3.** Trends in age incidence rates for Plague, females and males, 2004-2018 (Legends were the same to Fig S5-1). **Fig** **S5-4.** Trends in age incidence rates for Hepatitis D, females and males, 2004-2018 (Legends were the same to Fig S5-1). **Fig.** **S5-5.** Trends in age incidence rates for Hepatitis B, females and males, 2004-2018 (Legends were the same to Fig S5-1). **Fig.** **S5-6.** Trends in age incidence rates for NT, females and males, 2004-2018 (Legends were the same to Fig S5-1). **Fig.** **S5-7.** Trends in age incidence rates for Seasonal Influenza, females and males, 2004-2018 (Legends were the same to Fig S5-1). **Fig.** **S5-8.** Trends in age incidence rates for Mumps, females and males, 2004-2018 (Legends were the same to Fig S5-1). **Fig.** **S5-9.** Trends in age incidence rates for Hepatitis A, females and males, 2004-2018 (Legends were the same to Fig S5-1). **Fig** **S5-10.** Trends in age incidence rates for Diphtheria, females and males, 2004-2018 (Legends were the same to Fig S5-1). **Fig.** **S5-11.** Trends in age incidence rates for Pertussis, females and males, 2004-2018 (Legends were the same to Fig S5-1). **Fig.** **S5-12.** Trends in age incidence rates for Poliomyelitis, females and males, 2004-2018 (Legends were the same to Fig S5-1). **Fig.** **S5-13.** Trends in age incidence rates for Rubella, females and males, 2004-2018 (Legends were the same to Fig S5-1). **Fig.** **S5-14.** Trends in age incidence rates for Measles, females and males, 2004-2018 (Legends were the same to Fig S5-1). **Fig.** **S5-15.** Trends in age incidence rates for Typhoid and paratyphoid, females and males, 2004-2018 (Legends were the same to Fig S5-1). **Fig.** **S5-16.** Trends in age incidence rates for Infectious diarrhoea, females and males, 2004-2018 (Legends were the same to Fig S5-1). **Fig.** **S5-17.** Trends in age incidence rates for Acute haemorrhagic conjunctivitis, females and males, 2004-2018 (Legends were the same to Fig S5-1). **Fig.** **S5-18.** Trends in age incidence rates for Hand, foot, and mouth disease, females and males, 2004-2018 (Legends were the same to Fig S5-1). **Fig.** **S5-19.** Trends in age incidence rates for Dysentery, females and males, 2004-2018 (Legends were the same to Fig S5-1). **Fig.** **S5-20.** Trends in age incidence rates for Japanese encephalitis, females and males, 2004-2018 (Legends were the same to Fig S5-1). **Fig.** **S5-21.** Trends in age incidence rates for Typhus, females and males, 2004-2018 (Legends were the same to Fig S5-1). **Fig.** **S5-22.** Trends in age incidence rates for Malaria, females and males, 2004-2018 (Legends were the same to Fig S5-1). **Fig.** **S5-23.** Trends in age incidence rates for Schistosomiasis, females and males, 2004-2018 (Legends were the same to Fig S5-1). **Fig.** **S5-24.** Trends in age incidence rates for Filariasis, females and males, 2004-2018 (Legends were the same to Fig S5-1). **Fig.** **S5-25.** Trends in age incidence rates for Dengue, females and males, 2004-2018 (Legends were the same to Fig S5-1). **Fig.** **S5-26.** Trends in age incidence rates for Kala−azar, females and males, 2004-2018 (Legends were the same to Fig S5-1). **Fig.** **S5-27.** Trends in age incidence rates for H5N1, females and males, 2004-2018 (Legends were the same to Fig S5-1). **Fig.** **S5-28.** Trends in age incidence rates for H7N9, females and males, 2004-2018 (Legends were the same to Fig S5-1). **Fig.** **S5-29.** Trends in age incidence rates for Hydatid disease, females and males, 2004-2018 (Legends were the same to Fig S5-1). **Fig.** **S5-30.** Trends in age incidence rates for Brucellosis, females and males, 2004-2018 (Legends were the same to Fig S5-1). **Fig.** **S5-31.** Trends in age incidence rates for Hepatitis E, females and males, 2004-2018 (Legends were the same to Fig S5-1). **Fig.** **S5-32.** Trends in age incidence rates for Anthrax, females and males, 2004-2018 (Legends were the same to Fig S5-1). **Fig.** **S5-33.** Trends in age incidence rates for Rabies, females and males, 2004-2018 (Legends were the same to Fig S5-1). **Fig.** **S5-34.** Trends in age incidence rates for H1N1, females and males, 2004-2018 (Legends were the same to Fig S5-1). **Fig.** **S5-35.** Trends in age incidence rates for Leptospirosis, females and males, 2004-2018 (Legends were the same to Fig S5-1). **Fig.** **S5-36.** Trends in age incidence rates for SARS, females and males, 2004-2018 (Legends were the same to Fig S5-1). **Fig.** **S5-37.** Trends in age incidence rates for Scarlet fever, females and males, 2004-2018 (Legends were the same to Fig S5-1). **Fig.** **S5-38.** Trends in age incidence rates for Tuberculosis, females and males, 2004-2018 (Legends were the same to Fig S5-1). **Fig.** **S5-39.** Trends in age incidence rates for Meningococcal meningitis, females and males, 2004-2018 (Legends were the same to Fig S5-1). **Fig.** **S5-40.** Trends in age incidence rates for Leprosy, females and males, 2004-2018 (Legends were the same to Fig S5-1). **Fig.** **S5-41.** Trends in age incidence rates for Hepatitis C, females and males, 2004-2018 (Legends were the same to Fig S5-1). **Fig.** **S5-42.** Trends in age incidence rates for Syphilis, females and males, 2004-2018 (Legends were the same to Fig S5-1). **Fig.** **S5-43.** Trends in age incidence rates for Gonorrhoea, females and males, 2004-2018 (Legends were the same to Fig S5-1). **Fig.** **S5-44.** Trends in age incidence rates for HIV/AIDS, females and males, 2004-2018 (Legends were the same to Fig S5-1). **Fig.** **S6.** The seasonal variation by month of seven categories of 44 notifiable infectious diseases from 2004 to 2018.

## Data Availability

Data were obtained from China Information System for Disease Control and Prevention (CISDCP) (https://www.chinacdc.cn/) and the Public Health Science Data Center (https://www.phsciencedata.cn/Share/). All data generated or analysed during this study are included in this published article (and its supplementary information files).
