# Supplementary material for: Epidemiologic changes of infectious diseases in the post-SARS era in China, 2004–2018
Source: BMC Public Health. 2023 Nov 6;23:2171. doi: 10.1186/s12889-023-16756-8 (PMC10626686; doi:10.1186/s12889-023-16756-8)
Supplement: Supplementary file 1 — Additional file 1: Supplementary method. Joinpoint regression. Supplementary discussion. Table 1. The incidence of seven categories, total incidence and mortality of infectious diseases from 2004 to 2018, per 100,000. Table 2. Fifteen-year trends in incidence, number of cases, mortality and deaths of 44 notifiable infectious diseases in China, 2004-2018. Table 3. Changes in number of cases, incidence (per 100 000), number of deaths, and case-fatality ratios (per 1000) for 44 notifiable infectious diseases in China, 2004-2018. Table 4. The annual percentage changes (APC) and the joinpoint year range for 44 current notifiable infectious diseases in China from 2004 to 2018. Table 5. Incidence (per 100 000) of 44 infectious diseases stratified by age groups (years). Table 6. Incidence (per 100 000) of 44 infectious diseases stratified by gender. Table 7. Incidence (per 100 000) of 44 infectious diseases stratified by gender and transmission routes. Table 8. Incidence (per 100 000) of 44 infectious diseases stratified by gender and transmission routes. Fig. S1. The annual percentage changes (APC) and turning point in the trend from 2004 to 2018 for total and seven categories of infectious diseases using the joinpoint regression models. Fig. S2. Ranks of mortality or number of deaths for 44 notifiable infectious diseases during the past 15-years from 2004 to 2018. Fig. S3. The trends of incidence of each infectious disease in both genders and its joinpoint(s) during the past 15-years from 2004 to 2018. Fig. S3-1. The trends of incidence of Haemorrhagic Fever in both genders and its joinpoint(s) during the past 15-years from 2004 to 2018 (* represented the statistical significant trends. Legends were the same to Fig S1). Fig. S3-2. The trends of incidence of Cholera in both genders and its joinpoint(s) during the past 15-years from 2004 to 2018 (* represented the statistical significant trends. Legends were the same to Fig S1). Fig. S3-3. The trends of incidence of Hepatit [file 12889_2023_16756_MOESM1_ESM.docx]

**Supplementary information**

**Appendix Classification criteria: description and classification of 44 notifiable infectious diseases**

After the SARS outbreak in 2003, China updated and established a web-based CISDCP to rapidly identify new cases and respond to the outbreak. The system covers national notifiable infectious diseases in all 31 provinces in mainland China. We analyzed data for the fifteen years from 2004 to 2018. A total of 44 notifiable infectious diseases were included in this study. We adapted and revised Zhang and Wilson’s 2012 classification of 44 CISDCP notifiable infectious diseases into seven main categories reflecting the causes, characteristics and policy responses of the diseases. The main revision was to list hepatitis A, B and D as vaccine-preventable diseases. Hepatitis C was classified as an expanded category of sexually transmitted diseases and blood-borne infections, and hepatitis E was also classified as a zoonotic infection.

The 44 notifiable infectious diseases are divided into the following seven classes: quarantinable diseases, which include haemorrhagic fever, cholera, and plague; vaccine-preventable diseases, which include mumps, rubella, measles, seasonal Influenza, hepatitis B, hepatitis A, pertussis, hepatitis D, diphtheria, neonatal tetanus, and poliomyelitis; gastrointestinal or enterovirus borne diseases, which include OID (infectious diarrhoeal diseases other than cholera, bacterial and amoebic dysentery, typhoid and paratyphoid), dysentery, hand, foot, and mouth disease, acute haemorrhagic conjunctivitis, and typhoid/paratyphoid; vector-borne diseases, which include Malaria, japanese encephalitis, typhus, schistosomiasis, kala-azar, dengue, and filariasis; zoonotic infections, which include brucellosis, hepatitis E, rabies, hydatid disease, leptospirosis, anthrax, H7N9, H5N1, H1N1, and SARS; bacterial infections, which include tuberculosis, scarlet fever, meningococcal meningitis, and leprosy; sexually transmitted and blood-borne infections, gonorrhoea, syphilis, HIV/AIDS, and hepatitis C.

**Changes to the list of notifiable diseases in China, 2004-2018**

| **Year** | **Newly added** | **Cancel-reporting** |
| --- | --- | --- |
| 2004 | SARS and highly pathogenic avian influenza A (H5N1) |  |
| 2008 | Hand, foot and mouth disease (HFMD) |  |
| 2009 | Influenza A H1N1 |  |
| 2013 | H7N9 avian influenza | Influenza A H1N1 |

**Supplementary method****: Joinpoint regression**

The Joinpoint model is mainly used for the analysis of the trend change characteristics of time series data. The dependent variables of the applicable data are mainly the number of events (number of cases), morbidity/mortality or composition ratio, etc. The data distribution type can be selected when it conforms to normal distribution, exponential distribution or Poisson distribution. The Joinpoint regression model, also known as piecewise regression, broken-line or multi-phase regression, was proposed by Kim in 2000^1^. The basic idea is to divide a long-term trend line into segments, each segment is described by a continuous linear line. Commonly used linear models can only describe or predict one trend, and time series models also have many limitations The joinpoint regression model has no strict requirements on whether there is a trend in the data series itself. In recent years, more and more researchers have used it to determine the trend analysis of tumors, tuberculosis, AIDS, and smoking.

The Joinpoint regression model is based on the assumption that the Z test is used for segmentation points. First, it is assumed that there are no segmentation points, that is, H0: there are 0 segmentation points. At this time, traditional linear regression can be used for analysis; H1: There are at least one segmentation points. If H0 is rejected, then check whether the difference between one segment point and n segment points is statistically significant, and so on.

As suggested by the program developers, we allow up to 5 connection points for estimation and use the Bayesian Information Criterion (BIC) to select the best fitting model. APC = annual percent change. Joinpoint regression analysis was performed using the joinpoint regression program version 4.9.0 (National Cancer Institute Division of Statistical Research and Applications).

**Supplementary discussion**

***Quarantinable diseases***

Cholera has largely disappeared within the 15 years. The incidence of plague and hemorrhagic fever showed an overall downward trend, with relative APC of -21.7% (-27.6% to -15.3%, *P* <0.001) and -6.8% (-11.8% to -1.4%, *P* <0.1), respectively.

***Vaccine-Preventable Diseases***

Overall, two turning points were identified in 2008 and 2015 in the fifteen-year trend in vaccine-preventable diseases. During the studied period, the incidence of hepatitis A, measles, rubella and neonatal tetanus decreased to a lower level. For example, hepatitis A dropped from 7th to 17th and measles dropped from 9th to 22th, respectively in the incidence rankings. The incidence of hepatitis A decreased between 2004 and 2012 (APC=-15.4%, *P* <0.001), and the rate of decline slowed between 2012 and 2018 (APC = -7.3%, *P* =0.018). Measles incidence decreased from 5.43 to 0.28 per 100,000 population, with an 18.2% annual average decline (*P* =0.001). The incidence of rubella remained at a low level, and one joinpoint was identified in 2008. Rubella incidence increased noticeably, with 59.8% APC (*P* <0.001) before 2008, but decreased sharply with -32.9% APC (*P* <0.001 from 2008 to 2018). Incidence of hepatitis B, seasonal influenza, and mumps, which were the most common vaccine-preventable diseases in 2018, remained high. Over the 15 years, hepatitis B incidence was above 68 per 100,000 people, with two turning points in 2007 and 2015, rising before 2007 (APC=9.5%, *P* =0.008, 2004-2007) and decreasing from 2007 (APC= -3.7%, *P* =0.001, 2007-2015), and then remaining low (*P* =0.435, 2015-2018). The incidence of seasonal influenza increased nearly 14.5-fold, from 3.8 per 100,000 people in 2004 to 55.1 per 100,000 in 2018, a mean annual relative increase of 20.2% (*P* <0.001). There were no reported cases of diphtheria and poliomyelitis.

***Gastrointestinal and enteroviral diseases***

Two joinpoint years, 2007 and 2010, were identified in the fifteen-year gastrointestinal and enteroviral disease trends. The incidence of dysentery decreased steadily in the past 15 years, with an average annual decrease of 11.7% (*P* <0.001). During the study, HFMD incidence increased significantly from 37 to 169.4 cases per 100,000 people between 2008 and 2010 (APC=87.3%, *P* =0.04), remaining a high and stable incidence (*P* =0.412, 2010-2018). Obvious increase was also observed in other infectious diarrhea (OID) from 2004 to 2006, from 31.7 to 84.8 per 100,000 (APC=34.7%, *P* =0.04), followed by a stable trend to between 2006 and 2009 (*P* =0.826), and a slow incidence rise between 2009 and 2018 (APC=6.2%, *P* =0.001).

***Vector-borne diseases***

There was a general decline in vector-borne diseases except dengue fever. Sharp declines occurred in malaria, from 2.89 per 100,000 in 2004 to 0.18 per 100,000 in 2018 (APC=-17.9%, *P* <0.1, with a fall in ranking from 13th to 24th). Over the past 15 years, the rankings of Japanese encephalitis, typhus and schistosomiasis dropped from 21th, 23th and 2th8 in 2004 to 25th, 26th and 33th in 2018, respectively. The incidence of Japanese encephalitis declined rapidly before 2015, with -17.9% APC (*P* <0.001), then remained stable between 2015 and 2018 (*P* =0.332). Although the Typhus incidence continued to decline across the studied period, one turning point appeared in 2013, with a significant decrease APC of -14.8% (*P* <0.001). Similarly, 2015 was identified as the joinpoint in the trend for schistosomiasis, with a sharp decline in the following 4 years (APC=-73.9%, *P* <0.001, 2015-2018, extreme values for the 2015 schistosomiasis outbreak not excluded). Of all vector-borne diseases, Japanese encephalitis had the highest mortality rate, but it also showed a steady downward trend, from 0.015 per 100,000 in 2004 to 0.0097 per 100,000 in 2018. With a small outbreak in 2014 with an incidence rate of 3.46 per 100,000 people, the incidence of dengue fever increased from 0.019 to 0.37 per 100,000 over the past 15 years, showing a relative increase of 19.5 times, which increased its ranking from 32nd to 20th.

***Zoonotic Infections***

Except for brucellosis and hepatitis E, all zoonotic infections were at low risk. A low but stable incidence occurred in rabies (*P* =0.169), and one joinpoint was identified in 2007, followed by a 17.2% annual average decline until 2018 (*P* <0.001, 2007-2018). Rabies remains the No. 1 infectious cause of death. Leptospirosis showed a steady downward trend (APC=-12.6%, *P* <0.001), but the incidence of brucellosis, hydatid disease and hepatitis E showed a significant upward trend in the 15 years, with an average annual change of 6.6%, 16.6% and 3.9%, respectively. Although the joinpoint year of hydatid disease was identified in 2007, APC decreased from 68.8% (*P* <0.001, 2004-2007) to 5.4% (*P* =0.002, 2007-2018). Similarly, 2014 and 2012 were identified to be the joinpoint year respectively for brucellosis and hepatitis E. A significant increase in brucellosis was observed between 2004 and 2014 (APC=14.6%, *P* <0.001), followed by a stable trend until 2018 (*P* =0.075). A noticeably increase in hepatitis E was observed before 2012 (APC=6.8%, *P* <0.001), and then the incidence remained stable until 2018 (*P* =0.953). In addition to the 2009 H1N1 influenza outbreak (11.09 per 100,000 people), other avian influenza and SARS, including H7N9 and H5N1, occurred occasionally over the studied period.

***Bacterial infections***

During the studied period, the overall bacterial infections incidence decreased from 76.32 per 100 000 in 2004 to 64.97 per 100 000 in 2018 (APC=-2.6%, *P* <0.001). Tuberculosis maintained a steady decrease (APC=-3.1%, *P* <0.001). Although meningococcal meningitis declined by 22.6% over the 15 years, the incidence trends varied around 2012 (APC=29.9% vs 11.5%; *P* <0.001 vs *P* =0.014). Interestingly, the incidence of meningococcal meningitis in female showed two turning points in 2008 and 2011, with relative APC of 23.4% and -40.5% (*P* =0.002 vs *P* =0.025, 2004-2008 vs 2008-2011), respectively, followed by a slower decline in 2018 (APC=-14.3%, *P* <0.001, 2011-2018). In contrast, scarlet fever incidence became 3.89 times higher across the 15 years, from 1.46 to 5.68 per 100 000 (APC=9.5%, *P* <0.001, with a ranking rise from 16th in 2004 to 12th in 2018).

***Sexually transmitted diseases and bloodborne infections***

A turning point existed in 2011 in the 15-year trends in sexually transmitted diseases and blood-borne infections. The overall incidence first increased rapidly, with 10.0% APC (*P* <0.001, 2004-2011), and then slowed down, with 3.3% APC (*P* <0.001, 2011-2018). The incidence of hepatitis C increased from 3.03 to 15.79 per 100 000 between 2004 and 2018, a 5.2-fold relative increase over the studied period. Although the incidence of syphilis increased in the past 15 years, two jointpoint years appeared in 2006 and 2010 (APC=35.1%, *P* <0.001, 2004-2006; APC=20.9%, *P* <0.001, 2006-2010; APC=2.9%, *P* <0.001, 2010-2018). The year 2012 was the turning point of gonorrhoea, with an average annual decrease of 10.7% before 2012 (*P* <0.001, 2004-2012), and incidence increase after 2012 (APC=7.2%, *P* <0.001, 2012-2018). HIV/AIDS was one of the leading causes of death, besides rabies, avian influenza, and other highly lethal diseases. From 2004 to 2018, the HIV/AIDS cases increased by 7.5 times, with a sharp increase from 2004 to 2006 (APC=49.6%, *P* <0.001), and a slow increase from 2006 to 2018 (APC=9.9%, *P* <0.001).

**Table 1. The incidence of seven categories, total incidence and mortality of infectious diseases from 2004 to 2018, per 100,000**

| **Year** | **Incidence of Seven Categories** | | | | | | | **Total Incidence** | **Total**  **Mortality** |
| --- | --- | --- | --- | --- | --- | --- | --- | --- | --- |
|  | **Quarantinable**  **diseases** | **Vaccine-preventable**  **diseases** | **Gastrointestinal**  **or enterovirus**  **borne diseases** | **Vector-borne**  **diseases** | **Zoonotic**  **infections** | **Bacterial infections** | **Sexually**  **transmitted and**  **blood-borne infections** |  |  |
| 2004 | 1.95 | 106.82 | 74.70 | 3.77 | 2.55 | 76.32 | 28.51 | 294.63 | 0.49 |
| 2005 | 1.68 | 119.06 | 80.38 | 3.96 | 3.01 | 99.01 | 29.61 | 336.72 | 0.68 |
| 2006 | 1.17 | 126.07 | 91.62 | 5.77 | 3.34 | 88.50 | 32.77 | 349.23 | 0.74 |
| 2007 | 0.85 | 131.23 | 91.85 | 4.36 | 3.66 | 91.22 | 36.49 | 359.66 | 0.80 |
| 2008 | 0.70 | 138.81 | 118.87 | 2.68 | 4.01 | 90.72 | 40.73 | 396.51 | 0.79 |
| 2009 | 0.67 | 139.04 | 159.14 | 1.89 | 15.76 | 82.83 | 45.35 | 444.67 | 1.04 |
| 2010 | 0.73 | 115.63 | 230.63 | 1.28 | 5.25 | 75.89 | 49.65 | 479.06 | 1.06 |
| 2011 | 0.81 | 128.58 | 204.36 | 0.97 | 6.13 | 75.90 | 53.68 | 470.42 | 1.13 |
| 2012 | 0.99 | 130.80 | 245.38 | 0.88 | 5.43 | 74.12 | 56.55 | 514.16 | 1.28 |
| 2013 | 0.95 | 110.06 | 227.48 | 1.37 | 6.48 | 69.37 | 57.09 | 472.81 | 1.19 |
| 2014 | 0.85 | 105.74 | 284.49 | 4.19 | 6.61 | 69.67 | 58.40 | 529.95 | 1.17 |
| 2015 | 0.76 | 102.23 | 229.01 | 3.21 | 6.55 | 68.46 | 60.46 | 470.68 | 1.18 |
| 2016 | 0.65 | 108.02 | 264.75 | 0.79 | 5.94 | 65.36 | 61.85 | 507.36 | 1.24 |
| 2017 | 0.82 | 126.73 | 244.16 | 0.87 | 5.41 | 65.95 | 66.99 | 510.92 | 1.45 |
| 2018 | 0.86 | 149.08 | 271.82 | 0.77 | 5.17 | 64.97 | 68.55 | 561.22 | 1.72 |
| Joinpoint | 2008 | 2008;2015 | 2007;2010 | - | - | - | 2011 | 2010 | 2011;2016 |
| APC | -22.2(-32.0, -10.9)^2004-2008^ | 6.7(-0.1, 14.0)^2004-2008^  -4.6(-7.9, -1.2)^2008-2015^ | 6.7(-7.8, 23.5)^2004-2007^  33.6(-0.3,79.0)^2007-2010^ | -10.5 | 4.9 | -2.6 | 10.0*(9.0, 11.0)^2004-2011^ | 7.9(5.2, 10.7)^2004-2010^ | 11.6(7.7, 15.6)^2004-2011^  0.1(-7.9, 8.8)^2011-2016^ |
| (95%CI) | 1.5(-1.8, 4.9)^2008-2018^ | 12.5(1.3,24.9)^2015-2018^ | 2.5(-0.7, 5.8)^2010-2018^ | (-16.7, -3.8) | (-0.3, 10.3) | (-3.5, -1.7) | 3.3*(2.4, 4.2)^2011-2018^ | 1.5(-0.1, 3.2)^2010-2018^ | 19.7(-8.1, 55.9)^2016-2018^ |
| T values | -4.1; 1.0 | 2.3; -3.2; 2.7 | 1.0; 2.3; 1.8 | -3.3 | 2.1 | -6.3 | 23.7; 8.0 | 6.7; 2.0 | 7.4; 0.0; 1.6 |
| *P* values | 0.002; 0.351 | 0.052; 0.016; 0.032 | 0.330; 0.052; 0.110 | 0.005 | 0.061 | ＜0.001 | 1. ＜0.001 | ＜0.001; 0.070 | ＜0.001; 0.976; 0.152 |

Note: APC, annual percentage changes. We classified 44 notifiable infectious diseases into seven categories. Quarantinable diseases (HF, cholera, and plague); vaccine-preventable diseases (hepatitis D, hepatitis B, hepatitis A, NT, SI, mumps, diphtheria, pertussis, poliomyelitis, rubella, and measles); gastrointestinal or enterovirus borne diseases (T/P, OID, AHC, HFMD, and dysentery); vector-borne diseases (JE, typhus, malaria, schistosomiasis, filariasis, dengue, and Kala-azar); zoonotic diseases (H5N1, H7N9, hydatid disease, brucellosis, hepatitis E, anthrax, rabies, H1N1, leptospirosis, and SARS); bacterial diseases (scarlet fever, tuberculosis, meningococcal meningitis, and leprosy); sexually transmitted and blood-borne diseases (hepatitis C, syphilis, gonorrhoea, and HIV/AIDS).

**Table 2. Fifteen-year trends in incidence, number of cases, mortality and deaths of 44 notifiable infectious diseases in China, 2004-2018**

| **Year** | **Incidence per 100,000** | **Number of cases** | **Mortality per 100,000** | **Number of deaths** |
| --- | --- | --- | --- | --- |
| 2004 | 294.625 | 3832831 | 0.492 | 7135 |
| 2005 | 337.213 | 4388979 | 0.676 | 10110 |
| 2006 | 350.277 | 4586757 | 0.740 | 11018 |
| 2007 | 366.001 | 4820718 | 0.804 | 14490 |
| 2008 | 396.513 | 5249152 | 0.786 | 15779 |
| 2009 | 444.675 | 5918653 | 1.037 | 20367 |
| 2010 | 479.061 | 6410200 | 1.056 | 21834 |
| 2011 | 470.421 | 6328366 | 1.132 | 24406 |
| 2012 | 514.163 | 6969509 | 1.275 | 28758 |
| 2013 | 472.810 | 6444319 | 1.190 | 27550 |
| 2014 | 529.951 | 7226880 | 1.167 | 27842 |
| 2015 | 470.676 | 6463127 | 1.178 | 28810 |
| 2016 | 507.362 | 7009201 | 1.238 | 31065 |
| 2017 | 510.925 | 7107126 | 1.450 | 35261 |
| 2018 | 561.220 | 7860066 | 1.724 | 42729 |

**Table 3. Changes in number of cases, incidence (per 100 000), number of deaths, and case-fatality ratios (per 1000) for 44 notifiable infectious diseases in China, 2004-2018**

| **Disease classification** | **Cases (n)*** | **Yearly incidence (per 100 000) †** | **Deaths (n)*** | **Case-fatality ratios (per 1000)** | **Seasonal feature** |
| --- | --- | --- | --- | --- | --- |
| Total | 90615884 | 487.163 | 347154 | 3.83 | April to September |
| 1. Quarantinable diseases | | | | | |
| Haemorrhagic Fever | 190203 | 0.95 | 1850 | 9.73 | October to December |
| Cholera | 2208 | 0.01 | 8 | 3.62 | August to October |
| Plague | 63 | 0.0004 | 26 | 412.70 | July, September |
| 2. Vaccine-preventable diseases | | | | | |
| Mumps | 4269946 | 21.26 | 24 | 0.01 | April to July (May to June) ‡ |
| Rubella | 547838 | 2.75 | 6 | 0.01 | April to June |
| Measles | 797833 | 4.02 | 476 | 0.60 | March to June |
| Seasonal Influenza | 2751722 | 13.46 | 372 | 0.14 | December to March (January) ‡ |
| Hepatitis B | 15545074 | 77.40 | 9576 | 0.62 | Not significant |
| Hepatitis A | 631053 | 3.17 | 234 | 0.37 | Not significant |
| Pertussis | 74249 | 0.36 | 30 | 0.40 | May to August (July to -August) ‡ |
| Hepatitis D | 1142 | 0.03 | 0 | 0.00 | Not significant |
| Diphtheria | 3 | 0.0001 | 0 | 0.00 | May to July |
| Neonatal Tetanus | 17619 | 0.09 | 1680 | 95.35 | Not significant |
| Poliomyelitis | 20 | 0.001 | 1 | 50.00 | July to September |
| 3. Gastrointestinal or enterovirus borne diseases | | | | | |
| Other Infectious diarrhea § | 12696517 | 62.84 | 589 | 0.05 | June to December (July to August, November to December) ‡ |
| Dysentery | 3832188 | 19.26 | 658 | 0.17 | June to December (July to August) ‡ |
| Hand, Foot, and Mouth Disease | 20640579 | 137.53 | 3691 | 0.18 | May to October |
| Acute Haemorrhagic Conjunctivitis | 720640 | 3.58 | 1 | 0.001 | April to July (May to June) ‡ |
| Typhoid/Paratyphoid | 272932 | 1.37 | 89 | 0.33 | May to October (July to August) ‡ |
| 4. Vector-borne diseases | | | | | |
| Malaria | 256699 | 1.30 | 308 | 1.20 | June to October (July to October) ‡ |
| Japanese Encephalitis | 43153 | 0.22 | 2005 | 46.46 | July to August |
| Typhus | 33343 | 0.17 | 7 | 0.21 | May to November (October) ‡ |
| Schistosomiasis | 78656 | 0.39 | 15 | 0.19 | March to November (October to November) ‡ |
| Kala-azar | 4692 | 0.02 | 13 | 2.77 | Not significant |
| Dengue | 71759 | 0.35 | 10 | 0.14 | September to October |
| Filariasis | 19 | 0.0002 | 1 | 52.63 | Not significant |
| 5. Zoonotic infections |  |  |  |  |  |
| Brucellosis | 524980 | 2.60 | 16 | 0.03 | March to July (May to June) ‡ |
| Hepatitis E | 358122 | 1.78 | 434 | 1.21 | January to May (March) ‡ |
| Rabies | 26315 | 0.13 | 25691 | 976.29 | June to November |
| Hydatid disease | 45408 | 0.22 | 13 | 0.29 | Not significant |
| Leptospirosis | 9154 | 0.05 | 213 | 23.27 | August to September (September)‡ |
| Anthrax | 5294 | 0.03 | 59 | 11.14 | July to August |
| H7N9 | 1400 | 0.02 | 561 | 400.71 | January |
| H5N1 | 48 | 0.0003 | 33 | 687.50 | January |
| H1N1 | 175733 | 2.64 | 916 | 5.21 | October to December (November) ‡ |
| SARS | 10 | 0.001 | 1 | 100.00 | April |
| 6. Bacterial infections | | | | | |
| Tuberculosis | 14816329 | 73.90 | 42465 | 2.87 | January to May |
| Scarlet Fever | 655396 | 3.23 | 10 | 0.02 | May to June, November to December |
| Meningitis | 10990 | 0.06 | 1001 | 91.08 | February to April (March) ‡ |
| Leprosy | 5045 | 0.03 | 21 | 4.16 | January to April (January)‡ |
| 7. Sexually transmitted and blood-borne infections | | | | | |
| Gonorrhoea | 1938534 | 9.68 | 19 | 0.01 | Not significant |
| Syphilis | 4991580 | 24.65 | 935 | 0.19 | Not significant |
| HIV/AIDS | 1293116 | 6.36 | 251325 | 194.36 | Not significant |
| Hepatitis C | 2278280 | 11.25 | 1771 | 0.78 | Not significant |

* 15 years total.

†Mean 15 years.

‡Periods in parentheses represent more typical seasonal characteristics, and the incidence of each disease is more concentrated in this period.

§Infectious diarrheal diseases other than cholera, bacillary dysentery, amoebic dysentery, typhoid, and paratyphoid.

SARS = severe acute respiratory syndrome.

**Table 4. The annual percentage changes (APC) and the joinpoint year range for 44 current notifiable infectious diseases in China from 2004 to 2018**

| **Classification** | **Total** | | | **Boys** | | | **Girls** | | |
| --- | --- | --- | --- | --- | --- | --- | --- | --- | --- |
|  | **Joinpoint year range** | **APC, %** | ***P* Values** | **Joinpoint year range** | **APC, %** | ***P* Values** | **Joinpoint year range** | **APC, %** | ***P* Values** |
| **1. Quarantinable diseases** | | | | | | | | | |
| HF | 2004-2008 | -25.0*(-33.2, -15.7) | 0.001 | 2004-2008 | -24.5*(-32.5, -15.6) | 0.001 | 2004-2009 | -21.9*(-29.0, -14.2) | <0.001 |
|  | 2008-2012 | 8.8(-9.4, 30.7) | 0.314 | 2008-2012 | 8.0(-9.4, 28.8) | 0.335 | 2009-2012 | 20.3(-21.1, 83.4) | 0.335 |
|  | 2012-2018 | -2.8(-8.6, 3.4) | 0.315 | 2012-2018 | -3.0(-8.6, 2.9) | 0.260 | 2012-2018 | -3.0(-9.7, 4.2) | 0.344 |
|  | Full range | -6.8*(-11.8, -1.4) | <0.1 | Full range | -6.9*(-11.8, -1.8) | <0.1 | Full range | -6.0(-13.6, 2.2) | <0.1 |
| Cholera | 2004-2018 | -21.7*(-27.6, -15.3) | <0.001 | 2004-2018 | -21.5*(-27.8, -14.8) | <0.001 | 2004-2018 | -22.7*(-29.2, -15.6) | <0.001 |
| **2. Vaccine-preventable diseases** | | | | | | | | | |
| Hepatitis B | 2004-2007 | 9.5*(3.2, 16.2) | 0.008 | 2004-2007 | 8.2*(2.4, 14.4) | 0.012 | 2004-2007 | 11.9*(4.6,19.7) | 0.006 |
|  | 2007-2015 | -3.7*(-5.2, -2.1) | 0.001 | 2007-2015 | -4.0*(-5.4, -2.6) | <0.001 | 2007-2016 | -2.9*(-4.3, -1.4) | 0.002 |
|  | 2015-2018 | 2.1(-3.8, 8.3) | 0.435 | 2015-2018 | 2.9(-2.7, 8.8) | 0.264 | 2016-2018 | 2.7(-10.3, 17.6) | 0.656 |
|  | Full range | 0.3(-1.4, 1.9) | 1 | Full range | -0.0(-1.6, 1.5) | 1 | Full range | 0.9(-1.2, 3.1) | <0.1 |
| NT | 2004-2007 | -10.6(-24.4, 5.8) | 0.159 | 2004-2007 | -10.9(-22.2, 2.0) | 0.083 | 2004-2007 | -8.1(-25.7, 13.5) | 0.375 |
|  | 2007-2014 | -21.6*(-25.9, -17.0) | <0.001 | 2007-2014 | -23.0*(-26.4, -19.3) | <0.001 | 2007-2015 | -20.3*(-24.7, -15.7) | <0.001 |
|  | 2014-2018 | -35.2*(-41.7, -27.9) | <0.001 | 2014-2018 | -33.5*(-38.9, -27.5) | <0.001 | 2015-2018 | -43.3*(-54.1, -29.9) | <0.001 |
|  | Full range | -23.6*(-27.0, -20.1) | <0.1 | Full range | -23.8*(-26.5, -20.9) | <0.1 | Full range | -23.6*(-28.1, -19.0) | <0.1 |
| Sl | 2004-2018 | 20.2*(13.5, 27.2) | <0.001 | 2004-2018 | 19.4*(13.0, 26.0) | <0.001 | 2004-2018 | 21.3*(14.2, 28.9) | <0.001 |
| Mumps | 2004-2012 | 7.5*(2.3, 13.0) | 0.011 | 2004-2012 | 7.6*(2.4,13.1) | <0.01 | 2004-2012 | 7.3*(2.1, 12.8) | 0.012 |
|  | 2012-2015 | -27.7(-54.2, 14.2) | 0.138 | 2012-2015 | -28.6(-54.8,12.6) | 0.124 | 2012-2015 | -26.0(-53.2, 17.0) | 0.164 |
|  | 2015-2018 | 17.7(-6.3, 47.9) | 0.135 | 2015-2018 | 17.6(-6.4, 47.7) | 0.137 | 2015-2018 | 17.9(-6.3, 48.3) | 0.133 |
|  | Full range | 0.7(-8.3, 10.6) | 1 | Full range | 0.5(-8.5, 10.3) | 1 | Full range | 1.1(-8.0, 11.1) | 1 |
| Hepatitis A | 2004-2012 | -15.4*(-18.6, -12.1) | <0.001 | 2004-2012 | -16.3*(-19.3, -13.2) | <0.001 | 2004-2012 | -13.7*(-17.3, -10.0) | <0.001 |
|  | 2012-2018 | -7.3*(-12.7, -1.6) | 0.018 | 2012-2018 | -8.8*(-13.8, -3.5) | 0.004 | 2012-2018 | -5.0*(-11.1, 1.4) | 0.110 |
|  | Full range | -12.0*(-14.6, -9.4) | <0.1 | Full range | -13.2*(-15.6, -10.7) | <0.1 | Full range | -10.1*(-13.0, -7.1) | <0.1 |
| Pertussis | 2004-2013 | -8.0*(-14.5, -1.1) | 0.028 | 2004-2013 | -8.1*(-14.4, -1.4) | 0.023 | 2004-2013 | -7.9*(-14.6, -0.7) | 0.035 |
|  | 2013-2018 | 61.0*(34.8, 92.3) | <0.001 | 2013-2018 | 59.9*(34.5, 90.1) | <0.001 | 2013-2018 | 62.3*(35.0, 95.2) | <0.001 |
|  | Full range | 12.3*(4.8, 20.4) | <0.1 | Full range | 12.0*(4.7, 19.8) | <0.1 | Full range | 12.7*(4.9, 21.2) | <0.1 |
| Rubella | 2004-2008 | 59.8*(7.4, 137.7) | 0.025 | 2004-2008 | 60.2*(6.2, 141.8) | 0.029 | 2004-2008 | 59.3*(8.5, 133.8) | 0.022 |
|  | 2008-2018 | -32.9*(-39.2, -26.0) | <0.001 | 2008-2018 | -32.8*(-39.3, -25.7) | <0.001 | 2008-2018 | -33.1*(-39.1, -26.4) | <0.001 |
|  | Full range | -14.0*(-23.6, -3.4) | <0.1 | Full range | -13.9*(-23.7, -2.8) | <0.1 | Full range | -14.3*(-23.4, -4.0) | <0.1 |
| Measles | 2004-2018 | -18.2*(-26.3, -9.2) | 0.001 | 2004-2018 | -18.5*(-26.4, -9.7) | 0.001 | 2004-2018 | -17.9*(-26.2, -8.6) | 0.002 |
| **3. Gastrointestinal or enterovirus borne diseases** | | | | | | | | |  |
| T/P | 2004-2008 | -24.8*(-31.2, -17.7) | <0.001 | 2004-2008 | -25.4*(-31.1, -19.1) | <0.001 | 2004-2008 | -24.0*(-31.4, -15.8) | <0.001 |
|  | 2008-2018 | -4.2*(-6.3, -2.0) | 0.002 | 2008-2018 | -4.7*(-6.6, -2.8) | <0.001 | 2008-2018 | -3.5*(-5.9, -1.1) | 0.010 |
|  | Full range | -10.6*(-12.9, -8.2) | <0.1 | Full range | -11.1*(-13.2, -9.0) | <0.1 | Full range | -9.9*(-12.6, -7.1) | <0.1 |
| OID | 2004-2006 | 34.7*(1.8, 78.2) | 0.040 | 2004-2006 | 36.2*(1.6, 82.5) | 0.04 | 2004-2006 | 33.0*(1.8, 73.7) | 0.040 |
|  | 2006-2009 | -2.7(-26.4, 28.8) | 0.826 | 2006-2009 | -2.5(-27.2, 30.7) | 0.844 | 2006-2009 | -3.0(-25.7, 26.7) | 0.796 |
|  | 2009-2018 | 6.2*(3.5, 8.9) | 0.001 | 2009-2018 | 5.3*(2.6, 8.2) | 0.002 | 2009-2018 | 7.3*(4.7, 10.0) | <0.001 |
|  | Full range | 7.8*(1.4, 14.6) | <0.1 | Full range | 7.5*(0.8, 14.6) | <0.1 | Full range | 8.3*(2.1, 14.8) | <0.1 |
| AHC | 2004-2018 | 6.9(-3.8, 18.7) | 0.196 | 2004-2018 | 5.5(-4.9, 17.1) | 0.285 | 2004-2018 | 8.8(-2.3, 21.1) | 0.114 |
| HFMD | 2008-2010 | 87.3*(3.9, 237.8) | 0.04 | 2008-2010 | 86.8*(5.2, 231.5) | 0.037 | 2008-2010 | 88.5*(1.8, 249.1) | 0.045 |
|  | 2010-2018 | 2.3(-4.0, 9.1) | 0.412 | 2010-2018 | 1.6(-4.6, 8.2) | 0.559 | 2010-2018 | 3.6(-3.2, 10.8) | 0.250 |
|  | Full range | 15.5*(4.2, 28.0) | <0.1 | Full range | 14.8*(3.8, 26.9) | <0.1 | Full range | 16.7*(4.8, 30.0) | <0.1 |
| Dysentery | 2004-2018 | -11.7*(-12.2, -11.3) | <0.001 | 2004-2018 | -12.3*(-12.8, -11.8) | -49.7 | 2004-2018 | -11.0*(-11.4, -10.5) | <0.001 |
| **4. Vector-borne diseases** | | | | | | | | | |
| JE | 2004-2015 | -17.9*(-22.6, -12.9) | <0.001 | 2004-2015 | -18.6*(-23.2, -13.8) | <0.001 | 2004-2015 | -16.9*(-21.8, -11.6) | <0.001 |
|  | 2015-2018 | 22.3(-21.2, 89.7) | 0.332 | 2015-2018 | 19.5(-22.5, 84.1) | 0.381 | 2015-2018 | 25.8(-20.3, 98.4) | 0.289 |
|  | Full range | -10.6*(-18.5, -1.9) | <0.1 | Full range | -11.7*(-19.3, -3.2) | <0.1 | Full range | -9.2*(-17.5, -0.0) | <0.1 |
| Typhus | 2004-2013 | -7.2*(-9.6, -4.8) | <0.001 | 2004-2015 | -9.5*(-11.5, -7.5) | <0.001 | 2004-2012 | -4.3*(-7.2, -1.3) | 0.011 |
|  | 2013-2018 | -14.8*(-20.1, -9.2) | <0.001 | 2015-2018 | -16.0*(-28.7, -1.0) | 0.040 | 2012-2018 | -15.1*(-19.1, -10.9) | <0.001 |
|  | Full range | -10.0*(-12.3, -7.8) | <0.1 | Full range | -10.9*(-13.9, -7.8) | <0.1 | Full range | -9.1(-11.2, -6.9) | <0.1 |
| Malaria | 2004-2007 | 10.9(-14.2,43.2) | 0.373 | 2004-2006 | 33.9(-20.3, 124.9) | 0.225 | 2004-2007 | 24.1(-6.9, 65.4) | 0.119 |
|  | 2007-2011 | -49.2*(-60.7, -34.4) | <0.001 | 2006-2011 | -40.5*(-49.5, -29.9) | <0.001 | 2007-2013 | -58.2*(-63.2, -52.5) | <0.001 |
|  | 2011-2018 | -4.9(-11.2, 1.8) | 0.124 | 2011-2018 | -4.3(-10.7, 2.6) | 0.178 | 2013-2018 | 8.3(-4.8,23.1) | 0.186 |
|  | Full range | -17.9*(-24.3, -11.0) | <0.1 | Full range | -15.3*(-22.1, -7.9) | <0.1 | Full range | -25.8*(-31.4, -19.8) | <0.1 |
| Schistosomiasis | 2004-2015 | 18.2*(11.6, 25.3) | <0.001 | 2004-2015 | 17.0*(10.1, 24.3) | <0.001 | 2004-2015 | 20.1*(13.8, 26.7) | <0.001 |
|  | 2015-2018 | -73.9*(-83.0, -59.9) | <0.001 | 2015-2018 | -71.7*(-82.0, -55.7) | <0.001 | 2015-2018 | -77.6*(-85.0, -66.7) | <0.001 |
|  | Full range | -14.5*(-21.8, -6.4) | <0.1 | Full range | -13.7*(-21.5, -5.2) | <0.1 | Full range | -16.3*(-23.0, -8.9) | <0.1 |
| Dengue | 2004-2018 | 34.9*(13.6, 60.2) | 0.002 | 2004-2018 | 35.1*(14.9, 58.8) | 0.001 | 2004-2018 | 35.1*(12.3, 62.7) | 0.004 |
| Kala-azar | 2004-2018 | -3.8(-8.2, 0.8) | 0.095 | 2004-2018 | -4.0(-8.1, 0.2) | 0.061 | 2004-2018 | -3.6(-8.7, 1.8) | 0.172 |
| **5. Zoonotic infections** | | | | | | | | | |
| Hydatid disease | 2004-2007 | 68.8*(36.6, 108.6) | <0.001 | 2004-2007 | 65.6*(34.7, 103.7) | <0.001 | 2004-2007 | 71.9*(36.3, 116.6) | <0.001 |
|  | 2007-2018 | 5.4*(2.4, 8.5) | 0.002 | 2007-2018 | 5.3*(2.4, 8.3) | 0.002 | 2007-2018 | 5.5*(2.3, 8.9) | 0.003 |
|  | Full range | 16.6*(11.5, 21.9) | <0.1 | Full range | 16.0*(11.1, 21.2) | <0.1 | Full range | 17.2*(11.6, 23.0) | <0.1 |
| Brucellosis | 2004-2014 | 14.6*(11.0, 18.3) | <0.001 | 2004-2014 | 14.3*(10.8, 17.8) | <0.001 | 2004-2014 | 15.6*(11.3, 20.0) | <0.001 |
|  | 2014-2018 | -11.0(-21.9, 1.4) | 0.075 | 2014-2018 | -11.5(-21.9, 0.2) | 0.053 | 2014-2018 | -9.5(-22.3, 5.4) | 0.176 |
|  | Full range | 6.6*(2.6, 10.8) | <0.1 | Full range | 6.2*(2.4, 10.2) | <0.1 | Full range | 7.8*(3.1, 12.8) | <0.1 |
| Hepatitis E | 2004-2012 | 6.8*(4.2, 9.6) | <0.001 | 2004-2012 | 6.5*(3.7, 9.3) | <0.001 | 2004-2012 | 10.0*(7.6, 12.6) | <0.001 |
|  | 2012-2018 | 0.1(-3.8, 4.1) | 0.953 | 2012-2018 | -0.6(-3.1, 2.1) | 0.637 | 2012-2018 | 3.3(-0.3, 7.0) | 0.067 |
|  | Full range | 3.9*(1.9,6.0) | <0.1 | Full range | 2.9*(1.2, 4.6) | <0.1 | Full range | 7.1*(5.2, 9.0) | <0.1 |
| Anthrax | 2004-2013 | -11.4*(-13.3, -9.4) | <0.001 | 2004-2013 | -11.4*(-13.9, -8.9) | <0.001 | 2004-2013 | -11.3*(-13.6, -9.0) | <0.001 |
|  | 2013-2016 | 18.7(-7.1, 51.8) | 0.143 | 2013-2016 | 19.0(-12.6, 62.2) | 0.225 | 2013-2016 | 18.3(-10.6, 56.7) | 0.199 |
|  | 2016-2018 | -4.9(-25.6, 21.7) | 0.646 | 2016-2018 | -4.2(-29.7, 30.5) | 0.751 | 2016-2018 | -6.4(-29.4, 23.9) | 0.592 |
|  | Full range | -4.7* (-9.7, 0.6) | <0.1 | Full range | -4.6* (-10.8, 2.1) | <0.1 | Full range | -5.0*(-10.6, 1.1) | <0.1 |
| Rabies | 2004-2007 | 9.6(-4.5, 25.8) | 0.169 | 2004-2007 | 10.3(-4.4, 27.2) | 0.158 | 2004-2007 | 8.2(-7.9, 27.1) | 0.301 |
|  | 2007-2018 | -17.2*(-18.7, -15.6) | <0.001 | 2007-2018 | -17.0*(-18.5, -15.3) | <0.001 | 2007-2018 | -17.7*(-19.5, -15.9) | <0.001 |
|  | Full range | -12.1*(-14.6, -9.5) | <0.1 | Full range | -11.8*(-14.4, -9.1) | <0.1 | Full range | -12.7*(-15.6, -9.7) | <0.1 |
| Leptospirosis | 2004-2018 | -12.6*(-15.3, -9.9) | <0.001 | 2004-2018 | -12.5*(-14.8, -10.2) | <0.001 | 2004-2018 | -13.4*(-17.6, -8.9) | <0.001 |
| **6. Bacterial infections** | | | | | | | | | |
| Scarlet fever | 2004-2018 | 9.5*(5.9, 13.4) | <0.001 | 2004-2018 | 9.5*(5.8, 13.3) | <0.001 | 2004-2018 | 9.7*(6.0, 13.6) | <0.001 |
| Tuberculosis | 2004-2018 | -3.1*(-4.0, -2.2) | <0.001 | 2004-2018 | -3.2*(-4.1, -2.3) | <0.001 | 2004-2018 | -2.9*(-3.8, -2.0) | <0.001 |
| Meningococcal | 2004-2012 | -29.9*(-34.0, -25.6) | <0.001 | 2004-2011 | -30.9*(-36.2, -25.1) | <0.001 | 2004-2008 | -23.4*(-33.2, -12.2) | 0.002 |
| meningitis | 2012-2018 | -11.5*(-19.3, -3.0) | 0.014 | 2011-2018 | -12.6*(-19.4, -5.3) | 0.004 | 2008-2011 | -40.5*(-61.3, -8.4) | 0.025 |
|  |  |  |  |  |  |  | 2011-2018 | -14.3*(-19.1, -9.2) | <0.001 |
|  | Full range | -22.6*(-26.0, -18.9) | <0.1 | Full range | -22.3* (-26.1, -18.3) | <0.1 | Full range | -23.2*(-29.6, -16.3) | <0.1 |
| Leprosy | 2004-2009 | 14.1*(5.1, 23.9) | 0.005 | 2004-2009 | 15.5*(5.5, 26.4) | 0.005 | 2004-2008 | 15.0*(4.5, 26.5) | 0.009 |
|  | 2009-2018 | -6.2*(-9.3, -3.0) | 0.002 | 2009-2018 | -7.1*(-10.5, -3.6) | 0.001 | 2008-2018 | -3.4*(-5.6, -1.1) | 0.009 |
|  | Full range | 0.6(-2.6, 3.9) | 1 | Full range | 0.4(-3.1, 4.0) | 1 | Full range | 1.6(-1.3, 4.5) | <0.1 |
| **7. Sexually transmitted and blood-borne infections** | | | | | | | | | |
| Hepatitis C | 2004-2007 | 33.1*(29.7, 36.6) | <0.001 | 2004-2007 | 31.8*(28.4, 35.3) | <0.001 | 2004-2007 | 35.3*(31.5, 39.2) | <0.001 |
|  | 2007-2012 | 15.9*(14.0, 17.8) | <0.001 | 2007-2012 | 14.4*(12.5, 16.3) | <0.001 | 2007-2012 | 17.9*(15.8, 20.1) | <0.001 |
|  | 2012-2018 | 0.6(-0.3, 1.5) | 0.161 | 2012-2018 | 0.8(-0.1, 1.7) | 0.063 | 2012-2018 | 0.3(-0.7, 1.2) | 0.529 |
|  | Full range | 12.4*(11.5, 13.2) | <0.1 | Full range | 11.7*(10.9, 12.5) | <0.1 | Full range | 13.3*(12.4, 14.2) | <0.1 |
| Syphilis | 2004-2006 | 35.1*(25.4, 45.6) | <0.001 | 2004-2006 | 34.1*(19.7, 50.3) | <0.001 | 2004-2007 | 33.5*(30.2, 36.8) | <0.001 |
|  | 2006-2010 | 20.9*(16.5, 25.5) | <0.001 | 2006-2010 | 19.7*(13.1, 26.7) | <0.001 | 2007-2011 | 16.3*(13.5, 19.2) | <0.001 |
|  | 2010-2018 | 2.9*(2.1, 3.7) | <0.001 | 2010-2018 | 3.3*(2.0, 4.5) | <0.001 | 2011-2018 | 1.7*(1.0, 2.3) | 0.001 |
|  | Full range | 12.0*(10.6, 13.5) | <0.1 | Full range | 11.8*(9.6, 14.1) | <0.1 | Full range | 12.0*(11.1, 12.9) | <0.1 |
| Gonorrhoea | 2004-2012 | -10.7*(-12.4, -8.9) | <0.001 | 2004-2012 | -10.1*(-11.9, -8.3) | <0.001 | 2004-2013 | -11.7*(-12.8, -10.6) | <0.001 |
|  | 2012-2018 | 7.2*(4.0, 10.6) | <0.001 | 2012-2018 | 8.1*(4.7, 11.5) | <0.001 | 2013-2018 | 6.3*(3.2, 9.6) | 0.001 |
|  | Full range | -3.4*(-4.9, -1.9) | <0.1 | Full range | -2.7*(-4.2, -1.2) | <0.1 | Full range | -5.7*(-6.8, -4.5) | <0.1 |
| HIV/AIDS | 2004-2006 | 49.6*(32.5, 68.9) | <0.001 | 2004-2006 | 45.0*(9.4, 92.3) | 0.015 | 2004-2006 | 67.8*(35.4, 107.8) | <0.001 |
|  | 2006-2018 | 9.9*(9.1, 10.7) | <0.001 | 2006-2018 | 14.3*(12.4, 16.3) | <0.001 | 2006-2018 | 9.3*(7.9, 10.7) | <0.001 |
|  | Full range | 14.8*(13.0, 16.7) | <0.1 | Full range | 18.3*(13.9, 22.8) | <0.1 | Full range | 16.2*(12.9, 19.6) | <0.1 |

Note: Joinpoint regression cannot progress records with dependent variable=0, so the following several diseases did not have the results of Joinpoint regression (Plague, Hepatitis D, Diphtheria, Poliomyelitis, Filariasis, H7N9, H5N1, H1N1, and SARS). The results from HFMD were based on the entire period from 2008 to 2018.

**Table 5. Incidence (per 100 000) of 44 infectious diseases stratified by age groups (years)**

| **Diseases** | **0-** | **1-** | **2-** | **3-** | **4-** | **5-** | **6-** | **7-** | **8-** | **9-** | **10-** | **15-** | **20-** | **25-** | **30-** | **35-** | **40-** | **45-** | **50-** | **55-** | **60-** | **65-** | **70-** | **75-** | **80-** | **85-** | **Total** |
| --- | --- | --- | --- | --- | --- | --- | --- | --- | --- | --- | --- | --- | --- | --- | --- | --- | --- | --- | --- | --- | --- | --- | --- | --- | --- | --- | --- |
| HF | 0.02 | 0.02 | 0.03 | 0.03 | 0.05 | 0.07 | 0.09 | 0.07 | 0.09 | 0.12 | 0.24 | 0.45 | 0.55 | 0.90 | 1.16 | 1.22 | 1.41 | 1.45 | 1.75 | 1.45 | 1.53 | 1.31 | 0.99 | 0.70 | 0.49 | 0.35 | 0.95 |
| Cholera | 0.00 | 0.00 | 0.00 | 0.00 | 0.00 | 0.00 | 0.00 | 0.00 | 0.00 | 0.00 | 0.00 | 0.01 | 0.02 | 0.02 | 0.01 | 0.01 | 0.01 | 0.01 | 0.01 | 0.01 | 0.01 | 0.02 | 0.02 | 0.02 | 0.02 | 0.01 | 0.01 |
| Plague | 0.00 | 0.00 | 0.00 | 0.00 | 0.00 | 0.00 | 0.00 | 0.00 | 0.00 | 0.00 | 0.00 | 0.00 | 0.00 | 0.00 | 0.00 | 0.00 | 0.00 | 0.00 | 0.00 | 0.00 | 0.00 | 0.00 | 0.00 | 0.00 | 0.00 | 0.00 | 0.00 |
| Hepatitis D | 0.00 | 0.00 | 0.00 | 0.00 | 0.00 | 0.00 | 0.00 | 0.00 | 0.00 | 0.00 | 0.00 | 0.00 | 0.00 | 0.01 | 0.01 | 0.01 | 0.01 | 0.01 | 0.01 | 0.01 | 0.01 | 0.01 | 0.01 | 0.01 | 0.01 | 0.01 | 0.01 |
| Hepatitis B | 9.44 | 3.53 | 4.57 | 6.45 | 7.40 | 8.27 | 8.96 | 7.90 | 8.00 | 9.61 | 16.90 | 56.87 | 93.55 | 120.37 | 113.09 | 93.56 | 93.58 | 88.86 | 100.30 | 85.01 | 99.36 | 90.31 | 77.01 | 61.57 | 50.16 | 42.90 | 77.31 |
| NT | 7.35 | 0.00 | 0.00 | 0.00 | 0.00 | 0.00 | 0.00 | 0.00 | 0.00 | 0.00 | 0.00 | 0.00 | 0.00 | 0.00 | 0.00 | 0.00 | 0.00 | 0.00 | 0.00 | 0.00 | 0.00 | 0.00 | 0.00 | 0.00 | 0.00 | 0.00 | 0.09 |
| SI | 48.21 | 64.78 | 59.97 | 73.96 | 66.03 | 58.16 | 59.39 | 48.44 | 34.00 | 30.15 | 21.77 | 9.99 | 5.56 | 8.97 | 7.97 | 5.70 | 5.12 | 5.83 | 7.26 | 6.52 | 9.49 | 9.54 | 8.23 | 7.92 | 9.49 | 11.99 | 13.69 |
| Mumps | 5.96 | 17.74 | 41.97 | 91.71 | 138.90 | 171.60 | 190.92 | 165.71 | 140.77 | 140.78 | 83.99 | 17.06 | 4.88 | 5.02 | 4.67 | 2.84 | 1.67 | 1.19 | 1.16 | 0.95 | 0.99 | 0.81 | 0.66 | 0.55 | 0.52 | 0.60 | 21.24 |
| Hepatitis A | 1.01 | 2.12 | 4.98 | 6.77 | 7.40 | 7.34 | 7.39 | 5.87 | 4.91 | 4.86 | 3.89 | 2.48 | 2.22 | 2.89 | 3.35 | 2.92 | 2.83 | 2.44 | 3.03 | 2.56 | 3.05 | 3.10 | 3.22 | 2.92 | 2.88 | 2.79 | 3.14 |
| Diphtheria | 0.00 | 0.00 | 0.00 | 0.00 | 0.00 | 0.00 | 0.00 | 0.00 | 0.00 | 0.00 | 0.00 | 0.00 | 0.00 | 0.00 | 0.00 | 0.00 | 0.00 | 0.00 | 0.00 | 0.00 | 0.00 | 0.00 | 0.00 | 0.00 | 0.00 | 0.00 | 0.00 |
| Pertussis | 16.79 | 4.33 | 2.03 | 1.82 | 1.54 | 1.27 | 0.98 | 0.62 | 0.38 | 0.31 | 0.10 | 0.01 | 0.00 | 0.01 | 0.01 | 0.01 | 0.00 | 0.00 | 0.01 | 0.01 | 0.01 | 0.00 | 0.00 | 0.00 | 0.00 | 0.00 | 0.37 |
| Poliomyelitis | 0.00 | 0.00 | 0.00 | 0.00 | 0.00 | 0.00 | 0.00 | 0.00 | 0.00 | 0.00 | 0.00 | 0.00 | 0.00 | 0.00 | 0.00 | 0.00 | 0.00 | 0.00 | 0.00 | 0.00 | 0.00 | 0.00 | 0.00 | 0.00 | 0.00 | 0.00 | 0.00 |
| Rubella | 10.47 | 6.05 | 5.13 | 5.96 | 7.94 | 9.43 | 10.80 | 9.84 | 8.82 | 9.92 | 10.75 | 7.04 | 2.74 | 1.70 | 0.98 | 0.45 | 0.20 | 0.11 | 0.08 | 0.06 | 0.07 | 0.06 | 0.06 | 0.06 | 0.05 | 0.06 | 2.72 |
| Measles | 88.61 | 29.43 | 20.44 | 15.21 | 13.40 | 12.24 | 11.81 | 9.26 | 7.20 | 6.25 | 3.71 | 2.27 | 2.65 | 3.58 | 3.05 | 1.75 | 0.95 | 0.47 | 0.24 | 0.08 | 0.04 | 0.03 | 0.02 | 0.01 | 0.01 | 0.02 | 3.97 |
| T/P | 2.61 | 2.49 | 2.07 | 2.15 | 2.06 | 1.98 | 2.00 | 1.70 | 1.40 | 1.45 | 1.52 | 1.59 | 1.30 | 1.58 | 1.60 | 1.27 | 1.15 | 0.97 | 1.15 | 0.92 | 1.07 | 1.06 | 1.14 | 1.11 | 1.03 | 1.04 | 1.36 |
| OID | 1340.50 | 864.07 | 270.82 | 130.72 | 81.33 | 61.16 | 50.36 | 35.75 | 28.47 | 28.93 | 22.16 | 22.98 | 28.09 | 38.79 | 32.73 | 24.42 | 25.09 | 27.72 | 36.05 | 33.61 | 44.32 | 46.39 | 50.97 | 55.46 | 66.68 | 80.74 | 63.15 |
| AHC | 3.93 | 5.04 | 6.94 | 9.50 | 9.23 | 8.57 | 8.20 | 6.21 | 5.04 | 5.31 | 6.13 | 3.96 | 2.56 | 3.82 | 4.09 | 3.07 | 2.50 | 2.27 | 2.55 | 2.37 | 2.99 | 2.70 | 2.37 | 1.92 | 1.73 | 1.61 | 3.58 |
| HFMD | 814.85 | 2490.10 | 1891.82 | 1520.33 | 863.76 | 437.21 | 217.40 | 89.52 | 50.14 | 39.63 | 15.81 | 1.63 | 1.08 | 1.78 | 1.37 | 0.47 | 0.17 | 0.09 | 0.08 | 0.06 | 0.07 | 0.06 | 0.05 | 0.04 | 0.04 | 0.07 | 102.15 |
| Dysentery | 199.30 | 115.63 | 87.37 | 57.17 | 43.85 | 36.51 | 30.92 | 22.16 | 17.28 | 16.43 | 12.62 | 12.07 | 12.40 | 14.05 | 12.97 | 10.49 | 10.46 | 10.28 | 13.93 | 12.93 | 16.74 | 17.87 | 21.35 | 20.63 | 22.48 | 24.54 | 19.06 |
| JE | 0.59 | 0.85 | 1.69 | 2.02 | 1.97 | 1.74 | 1.57 | 1.05 | 0.76 | 0.67 | 0.35 | 0.07 | 0.04 | 0.03 | 0.03 | 0.03 | 0.04 | 0.04 | 0.07 | 0.06 | 0.10 | 0.10 | 0.11 | 0.09 | 0.05 | 0.03 | 0.21 |
| Typhus | 0.09 | 0.18 | 0.29 | 0.33 | 0.35 | 0.34 | 0.37 | 0.29 | 0.25 | 0.27 | 0.20 | 0.13 | 0.12 | 0.15 | 0.16 | 0.15 | 0.15 | 0.16 | 0.18 | 0.14 | 0.17 | 0.15 | 0.14 | 0.13 | 0.11 | 0.08 | 0.17 |
| Malaria | 0.30 | 0.41 | 0.78 | 0.90 | 0.86 | 0.95 | 1.09 | 0.89 | 0.84 | 0.93 | 1.47 | 1.31 | 1.08 | 1.43 | 1.67 | 1.56 | 1.54 | 1.06 | 1.25 | 1.07 | 1.29 | 1.30 | 1.52 | 1.18 | 1.30 | 1.01 | 1.28 |
| Schistosomiasis | 0.00 | 0.00 | 0.00 | 0.01 | 0.01 | 0.02 | 0.03 | 0.03 | 0.04 | 0.06 | 0.07 | 0.07 | 0.09 | 0.18 | 0.25 | 0.33 | 0.52 | 0.75 | 0.96 | 0.80 | 1.16 | 0.80 | 0.56 | 0.38 | 0.22 | 0.13 | 0.39 |
| Filariasis | 0.00 | 0.00 | 0.00 | 0.00 | 0.00 | 0.00 | 0.00 | 0.00 | 0.00 | 0.00 | 0.00 | 0.00 | 0.00 | 0.00 | 0.00 | 0.00 | 0.00 | 0.00 | 0.00 | 0.00 | 0.00 | 0.00 | 0.00 | 0.00 | 0.00 | 0.00 | 0.00 |
| Dengue | 0.07 | 0.08 | 0.11 | 0.11 | 0.12 | 0.14 | 0.15 | 0.13 | 0.13 | 0.15 | 0.16 | 0.21 | 0.37 | 0.56 | 0.51 | 0.37 | 0.40 | 0.39 | 0.41 | 0.38 | 0.46 | 0.43 | 0.39 | 0.46 | 0.52 | 0.57 | 0.36 |
| Kala-azar | 0.40 | 0.32 | 0.14 | 0.09 | 0.07 | 0.05 | 0.05 | 0.03 | 0.03 | 0.03 | 0.02 | 0.01 | 0.01 | 0.01 | 0.01 | 0.01 | 0.01 | 0.01 | 0.01 | 0.01 | 0.01 | 0.01 | 0.00 | 0.00 | 0.00 | 0.00 | 0.02 |
| H5N1 | 0.00 | 0.00 | 0.00 | 0.00 | 0.00 | 0.00 | 0.00 | 0.00 | 0.00 | 0.00 | 0.00 | 0.00 | 0.00 | 0.00 | 0.00 | 0.00 | 0.00 | 0.00 | 0.00 | 0.00 | 0.00 | 0.00 | 0.00 | 0.00 | 0.00 | 0.00 | 0.00 |
| H7N9 | 0.00 | 0.00 | 0.00 | 0.00 | 0.00 | 0.00 | 0.00 | 0.00 | 0.00 | 0.00 | 0.00 | 0.00 | 0.00 | 0.00 | 0.00 | 0.01 | 0.00 | 0.01 | 0.01 | 0.02 | 0.02 | 0.02 | 0.02 | 0.03 | 0.03 | 0.01 | 0.01 |
| Hydatid disease | 0.00 | 0.00 | 0.01 | 0.03 | 0.05 | 0.06 | 0.10 | 0.09 | 0.08 | 0.11 | 0.10 | 0.10 | 0.14 | 0.25 | 0.31 | 0.30 | 0.30 | 0.28 | 0.29 | 0.25 | 0.34 | 0.37 | 0.36 | 0.27 | 0.18 | 0.15 | 0.23 |
| Brucellosis | 0.07 | 0.22 | 0.36 | 0.43 | 0.43 | 0.43 | 0.43 | 0.35 | 0.31 | 0.36 | 0.43 | 0.63 | 1.16 | 2.34 | 2.99 | 3.16 | 3.99 | 4.61 | 5.43 | 4.60 | 4.94 | 3.50 | 2.04 | 1.19 | 0.67 | 0.39 | 2.61 |
| Hepatitis E | 0.16 | 0.06 | 0.07 | 0.07 | 0.06 | 0.08 | 0.07 | 0.05 | 0.05 | 0.06 | 0.07 | 0.22 | 0.53 | 1.08 | 1.40 | 1.54 | 2.06 | 2.64 | 3.61 | 3.55 | 4.53 | 4.46 | 4.31 | 3.89 | 3.64 | 3.30 | 1.78 |
| Anthrax | 0.00 | 0.00 | 0.01 | 0.01 | 0.01 | 0.01 | 0.01 | 0.01 | 0.01 | 0.01 | 0.01 | 0.01 | 0.02 | 0.03 | 0.04 | 0.04 | 0.04 | 0.03 | 0.03 | 0.03 | 0.03 | 0.02 | 0.02 | 0.01 | 0.01 | 0.01 | 0.03 |
| Rabies | 0.00 | 0.04 | 0.13 | 0.17 | 0.20 | 0.19 | 0.21 | 0.17 | 0.14 | 0.16 | 0.14 | 0.05 | 0.03 | 0.05 | 0.07 | 0.09 | 0.12 | 0.13 | 0.21 | 0.23 | 0.28 | 0.29 | 0.30 | 0.24 | 0.17 | 0.15 | 0.13 |
| H1N1 | 0.74 | 0.80 | 1.03 | 1.43 | 1.44 | 1.71 | 2.16 | 2.34 | 2.28 | 2.91 | 3.38 | 2.91 | 0.98 | 0.68 | 0.33 | 0.22 | 0.17 | 0.14 | 0.18 | 0.17 | 0.13 | 0.09 | 0.11 | 0.11 | 0.08 | 0.07 | 0.87 |
| Leptospirosis | 0.00 | 0.00 | 0.00 | 0.00 | 0.00 | 0.01 | 0.01 | 0.02 | 0.01 | 0.02 | 0.04 | 0.04 | 0.03 | 0.04 | 0.05 | 0.05 | 0.06 | 0.05 | 0.08 | 0.07 | 0.08 | 0.06 | 0.04 | 0.02 | 0.02 | 0.01 | 0.05 |
| SARS | 0.00 | 0.00 | 0.00 | 0.00 | 0.00 | 0.00 | 0.00 | 0.00 | 0.00 | 0.00 | 0.00 | 0.00 | 0.00 | 0.00 | 0.00 | 0.00 | 0.00 | 0.00 | 0.00 | 0.00 | 0.00 | 0.00 | 0.00 | 0.00 | 0.00 | 0.00 | 0.00 |
| Scarlet fever | 1.69 | 4.42 | 8.76 | 22.91 | 43.08 | 54.07 | 54.61 | 34.74 | 19.22 | 13.01 | 3.79 | 0.59 | 0.20 | 0.14 | 0.08 | 0.04 | 0.02 | 0.02 | 0.01 | 0.01 | 0.01 | 0.01 | 0.01 | 0.01 | 0.01 | 0.01 | 3.26 |
| Tuberculosis | 4.61 | 2.83 | 3.01 | 3.04 | 3.38 | 3.61 | 4.19 | 3.85 | 3.55 | 3.98 | 8.08 | 56.35 | 81.07 | 79.05 | 68.99 | 60.52 | 65.95 | 70.05 | 94.92 | 98.55 | 142.65 | 168.34 | 197.19 | 187.54 | 166.21 | 137.03 | 73.69 |
| Meningococcal meningitis | 0.36 | 0.21 | 0.19 | 0.16 | 0.16 | 0.15 | 0.16 | 0.15 | 0.13 | 0.14 | 0.17 | 0.11 | 0.04 | 0.02 | 0.02 | 0.02 | 0.02 | 0.01 | 0.02 | 0.01 | 0.01 | 0.01 | 0.01 | 0.01 | 0.00 | 0.00 | 0.05 |
| Leprosy | 0.00 | 0.00 | 0.00 | 0.00 | 0.00 | 0.00 | 0.00 | 0.00 | 0.00 | 0.01 | 0.01 | 0.01 | 0.02 | 0.03 | 0.03 | 0.03 | 0.03 | 0.03 | 0.04 | 0.03 | 0.04 | 0.04 | 0.04 | 0.03 | 0.02 | 0.01 | 0.03 |
| Hepatitis C | 6.90 | 0.70 | 0.53 | 0.47 | 0.38 | 0.36 | 0.37 | 0.27 | 0.25 | 0.25 | 0.39 | 1.56 | 4.44 | 8.59 | 11.53 | 12.83 | 15.21 | 16.74 | 19.15 | 17.29 | 23.43 | 24.33 | 24.91 | 25.23 | 25.27 | 26.08 | 11.33 |
| Syphilis | 44.77 | 1.44 | 0.77 | 0.58 | 0.44 | 0.36 | 0.33 | 0.27 | 0.19 | 0.20 | 0.28 | 7.39 | 27.21 | 41.41 | 37.60 | 28.32 | 26.43 | 25.91 | 27.58 | 23.55 | 32.78 | 38.55 | 42.49 | 46.96 | 56.85 | 85.73 | 24.83 |
| Gonorrhoea | 4.08 | 0.31 | 0.47 | 0.64 | 0.70 | 0.75 | 0.77 | 0.56 | 0.40 | 0.33 | 0.17 | 5.81 | 18.32 | 25.59 | 21.65 | 13.93 | 10.69 | 7.32 | 7.43 | 5.48 | 5.09 | 3.47 | 2.49 | 1.70 | 1.26 | 1.29 | 9.64 |
| HIV/AIDS | 0.17 | 0.48 | 0.78 | 0.65 | 0.60 | 0.55 | 0.50 | 0.36 | 0.29 | 0.28 | 0.21 | 2.13 | 7.61 | 12.76 | 12.32 | 9.22 | 7.95 | 6.91 | 6.80 | 5.66 | 7.62 | 7.59 | 6.54 | 5.22 | 3.73 | 2.62 | 6.43 |

Note: HF = haemorrhagic fever. NT = neonatal tetanus. SI = seasonal influenza. T/P = typhoid/paratyphoid. OID = infectious diarrhoeal diseases other than cholera, bacterial and amoebic dysentery, typhoid, and paratyphoid. AHC = acute haemorrhagic conjunctivitis. HFMD = hand, foot, and mouth disease. JE = japanese encephalitis. SARS=severe acute respiratory syndrome.

**Table 6. Incidence (per 100 000) of 44 infectious diseases stratified by gender**

| **Diseases** | **Male**  **(n=10 306 546 523)** | **Female**  **(n=9 798 954 249)** | **Total**  **(n=20 105 500 772)** | **Sex ratio**  **(male/female)** |
| --- | --- | --- | --- | --- |
| **1.** **Quarantinable diseases** | | | | |
| HF | 1.38 | 0.49 | 0.95 | 2.8 |
| Cholera | 0.01 | 0.01 | 0.01 | 1.1 |
| Plague | 0.00 | 0.00 | 0.00 | 4.0 |
| **2.** **Vaccine-preventable diseases** | | | | |
| Hepatitis D | 0.01 | 0.00 | 0.01 | 1.5 |
| Hepatitis B | 96.52 | 57.12 | 77.31 | 1.7 |
| NT | 0.12 | 0.06 | 0.09 | 2.0 |
| SI | 14.89 | 12.42 | 13.69 | 1.2 |
| Mumps | 25.78 | 16.46 | 21.24 | 1.6 |
| Hepatitis A | 3.91 | 2.33 | 3.14 | 1.7 |
| Diphtheria | 0.00 | 0.00 | 0.00 | 0.5 |
| Pertussis | 0.38 | 0.35 | 0.37 | 1.1 |
| Poliomyelitis | 0.00 | 0.00 | 0.00 | 1.8 |
| Rubella | 3.02 | 2.42 | 2.72 | 1.2 |
| Measles | 4.55 | 3.35 | 3.97 | 1.4 |
| **3. Gastrointestinal or enterovirus borne diseases** | | | | |
| T/P | 1.44 | 1.27 | 1.36 | 1.1 |
| OID | 71.25 | 54.63 | 63.15 | 1.3 |
| AHC | 4.03 | 3.12 | 3.58 | 1.3 |
| HFMD | 121.67 | 81.62 | 102.15 | 1.5 |
| Dysentery | 21.40 | 16.59 | 19.06 | 1.3 |
| **4. Vector-borne diseases** | | | | |
| JE | 0.25 | 0.18 | 0.21 | 1.4 |
| Typhus | 0.16 | 0.17 | 0.17 | 1.0 |
| Malaria | 1.71 | 0.82 | 1.28 | 2.1 |
| Schistosomiasis | 0.47 | 0.31 | 0.39 | 1.5 |
| Filariasis | 0.00 | 0.00 | 0.00 | 1.6 |
| Dengue | 0.35 | 0.36 | 0.36 | 1.0 |
| Kala-azar | 0.03 | 0.02 | 0.02 | 1.5 |
| **5.** **Zoonotic infections** | | | | |
| H5N1 | 0.00 | 0.00 | 0.00 | 1.0 |
| H7N9 | 0.01 | 0.00 | 0.01 | 2.2 |
| Hydatid disease | 0.21 | 0.24 | 0.23 | 0.9 |
| Brucellosis | 3.76 | 1.40 | 2.61 | 2.7 |
| Hepatitis E | 2.47 | 1.05 | 1.78 | 2.3 |
| Anthrax | 0.04 | 0.01 | 0.03 | 2.7 |
| Rabies | 0.18 | 0.08 | 0.13 | 2.2 |
| H1N1 | 0.97 | 0.77 | 0.87 | 1.3 |
| Leptospirosis | 0.06 | 0.03 | 0.05 | 2.1 |
| SARS | 0.00 | 0.00 | 0.00 | 0.4 |
| **6.** **Bacterial** **infections** | | | | |
| Scarlet fever | 3.90 | 2.58 | 3.26 | 1.5 |
| Tuberculosis | 99.77 | 46.26 | 73.69 | 2.2 |
| Meningococcal meningitis | 0.07 | 0.04 | 0.05 | 1.6 |
| Leprosy | 0.03 | 0.02 | 0.03 | 2.3 |
| **7.** **Sexually transmitted and blood-borne infections** | | | | |
| Hepatitis C | 12.57 | 10.03 | 11.33 | 1.3 |
| Syphilis | 23.48 | 26.24 | 24.83 | 0.9 |
| Gonorrhoea | 15.21 | 3.79 | 9.64 | 4.0 |
| HIV/AIDS | 9.40 | 3.31 | 6.43 | 2.8 |
| **Total** | 545.46 | 349.95 | 450.17 | 1.6 |

Note: We classified 44 notifiable infectious diseases into seven categories. Quarantinable diseases (HF, cholera, and plague); vaccine-preventable diseases (hepatitis D, hepatitis B, hepatitis A, NT, SI, mumps, diphtheria, pertussis, poliomyelitis, rubella, and measles); gastrointestinal or enterovirus borne diseases (T/P, OID, AHC, HFMD, and dysentery); vector-borne diseases (JE, typhus, malaria, schistosomiasis, filariasis, dengue, and Kala-azar); zoonotic diseases (H5N1, H7N9, hydatid disease, brucellosis, hepatitis E, anthrax, rabies, H1N1, leptospirosis, and SARS); bacterial diseases (scarlet fever, tuberculosis, meningococcal meningitis, and leprosy); sexually transmitted and blood-borne diseases (hepatitis C, syphilis, gonorrhoea, and HIV/AIDS). HF = haemorrhagic fever. NT = neonatal tetanus. SI = seasonal influenza. T/P = typhoid/paratyphoid. OID = infectious diarrhoeal diseases other than cholera, bacterial and amoebic dysentery, typhoid, and paratyphoid. AHC = acute haemorrhagic conjunctivitis. HFMD = hand, foot, and mouth disease. JE = japanese encephalitis. SARS=severe acute respiratory syndrome.

**Table 7. Incidence (per 100 000) of 44 infectious diseases stratified by gender and transmission routes**

| **Year** | **Population (n)** | **Quarantinable diseases** | **Vaccine-**  **preventable diseases** | **Gastrointestinal or enterovirus borne diseases** | **Vector-borne diseases** | **Zoonotic infections** | **Bacterial infections** | **Sexually transmitted and blood-borne infections** |
| --- | --- | --- | --- | --- | --- | --- | --- | --- |
| **Male** | | | | | | | | |
| 2004 | 670088764 | 2.82 | 134.72 | 84.87 | 4.92 | 3.73 | 102.79 | 38.74 |
| 2005 | 669828077 | 2.44 | 149.08 | 92.01 | 5.16 | 4.37 | 133.53 | 38.67 |
| 2006 | 660861944 | 1.75 | 159.69 | 105.82 | 7.25 | 4.93 | 120.99 | 41.77 |
| 2007 | 677279999 | 1.25 | 162.36 | 105.28 | 5.29 | 5.18 | 122.90 | 44.48 |
| 2008 | 680480000 | 1.02 | 170.71 | 139.83 | 3.27 | 5.63 | 122.63 | 48.38 |
| 2009 | 681168553 | 1.00 | 171.27 | 189.67 | 2.33 | 19.04 | 112.01 | 53.00 |
| 2010 | 684246080 | 1.08 | 142.55 | 274.56 | 1.63 | 7.19 | 102.84 | 56.88 |
| 2011 | 687479997 | 1.18 | 156.84 | 244.57 | 1.21 | 8.48 | 102.70 | 61.26 |
| 2012 | 690679957 | 1.45 | 159.09 | 293.15 | 1.10 | 7.63 | 100.00 | 66.32 |
| 2013 | 693950000 | 1.35 | 132.21 | 263.95 | 1.67 | 8.73 | 93.07 | 67.08 |
| 2014 | 695577461 | 1.22 | 125.73 | 328.14 | 4.31 | 9.19 | 93.26 | 69.29 |
| 2015 | 697969507 | 1.10 | 122.91 | 263.47 | 3.78 | 8.99 | 91.37 | 73.06 |
| 2016 | 702175237 | 0.94 | 128.55 | 302.85 | 1.09 | 8.02 | 87.31 | 76.42 |
| 2017 | 705306784 | 1.18 | 149.84 | 277.44 | 1.09 | 7.24 | 88.43 | 83.68 |
| 2018 | 709454163 | 1.23 | 173.15 | 308.74 | 0.96 | 6.89 | 86.46 | 86.46 |
| **Female** | | | | | | | | |
| 2004 | 629791236 | 1.02 | 77.02 | 63.82 | 2.53 | 1.30 | 48.00 | 18.09 |
| 2005 | 630051796 | 0.88 | 87.11 | 68.01 | 2.68 | 1.57 | 62.29 | 20.87 |
| 2006 | 646697966 | 0.57 | 91.72 | 77.12 | 4.26 | 1.72 | 55.29 | 24.59 |
| 2007 | 637196401 | 0.44 | 98.14 | 77.57 | 3.38 | 2.04 | 57.54 | 29.53 |
| 2008 | 640810000 | 0.35 | 104.94 | 96.60 | 2.04 | 2.29 | 56.83 | 34.19 |
| 2009 | 646851446 | 0.31 | 105.11 | 126.99 | 1.43 | 12.29 | 52.11 | 39.35 |
| 2010 | 650493923 | 0.36 | 87.32 | 184.42 | 0.91 | 3.22 | 47.55 | 44.51 |
| 2011 | 653429999 | 0.41 | 98.84 | 162.05 | 0.71 | 3.67 | 47.70 | 48.83 |
| 2012 | 656670039 | 0.51 | 101.06 | 195.15 | 0.66 | 3.12 | 46.89 | 52.66 |
| 2013 | 660090000 | 0.53 | 86.77 | 189.14 | 1.06 | 4.13 | 44.45 | 53.00 |
| 2014 | 659591139 | 0.47 | 84.66 | 238.45 | 4.06 | 3.89 | 44.79 | 53.76 |
| 2015 | 664497179 | 0.40 | 80.51 | 192.80 | 2.61 | 3.99 | 44.39 | 54.81 |
| 2016 | 668609163 | 0.34 | 86.47 | 224.74 | 0.47 | 3.76 | 42.30 | 54.68 |
| 2017 | 674531172 | 0.44 | 102.57 | 209.36 | 0.64 | 3.50 | 42.44 | 58.01 |
| 2018 | 679642790 | 0.48 | 123.95 | 233.28 | 0.57 | 3.37 | 42.55 | 59.30 |

Note: We classified 44 notifiable infectious diseases into seven categories. Quarantinable diseases (HF, cholera, and plague); vaccine-preventable diseases (hepatitis D, hepatitis B, hepatitis A, NT, SI, mumps, diphtheria, pertussis, poliomyelitis, rubella, and measles); gastrointestinal or enterovirus borne diseases (T/P, OID, AHC, HFMD, and dysentery); vector-borne diseases (JE, typhus, malaria, schistosomiasis, filariasis, dengue, and Kala-azar); zoonotic diseases (H5N1, H7N9, hydatid disease, brucellosis, hepatitis E, anthrax, rabies, H1N1, leptospirosis, and SARS); bacterial diseases (scarlet fever, tuberculosis, meningococcal meningitis, and leprosy); sexually transmitted and blood-borne diseases (hepatitis C, syphilis, gonorrhoea, and HIV/AIDS).

**Table 8. Incidence (per 100 000) of 44 infectious diseases stratified by gender and transmission routes**

| **Gender** | **Population(n)** | **Quarantinable diseases** | **Vaccine-**  **preventable diseases** | **Gastrointestinal or enterovirus borne diseases** | **Vector-borne diseases** | **Zoonotic infections** | **Bacterial infections** | **Sexually transmitted and blood-borne infections** | **Total** |
| --- | --- | --- | --- | --- | --- | --- | --- | --- | --- |
| Male | 10306546523 | 1.39 | 149.17 | 219.79 | 2.98 | 7.70 | 103.77 | 60.66 | 545.46 |
| Female | 9798954249 | 0.50 | 94.51 | 157.23 | 1.85 | 3.60 | 48.90 | 43.36 | 349.95 |
| Total | 20105500772 | 0.96 | 122.53 | 189.30 | 2.43 | 5.70 | 77.03 | 52.23 | 450.17 |

Note: We classified 44 notifiable infectious diseases into seven categories. Quarantinable diseases (HF, cholera, and plague); vaccine-preventable diseases (hepatitis D, hepatitis B, hepatitis A, NT, SI, mumps, diphtheria, pertussis, poliomyelitis, rubella, and measles); gastrointestinal or enterovirus borne diseases (T/P, OID, AHC, HFMD, and dysentery); vector-borne diseases (JE, typhus, malaria, schistosomiasis, filariasis, dengue, and Kala-azar); zoonotic diseases (H5N1, H7N9, hydatid disease, brucellosis, hepatitis E, anthrax, rabies, H1N1, leptospirosis, and SARS); bacterial diseases (scarlet fever, tuberculosis, meningococcal meningitis, and leprosy); sexually transmitted and blood-borne diseases (hepatitis C, syphilis, gonorrhoea, and HIV/AIDS).

**References**

1. Kim, H. J., Fay, M. P., Feuer, E. J. & Midthune, D. N. Permutation tests for joinpoint regression with applications to cancer rates. *Stat. Med.* **19**, 335-351 (2000).


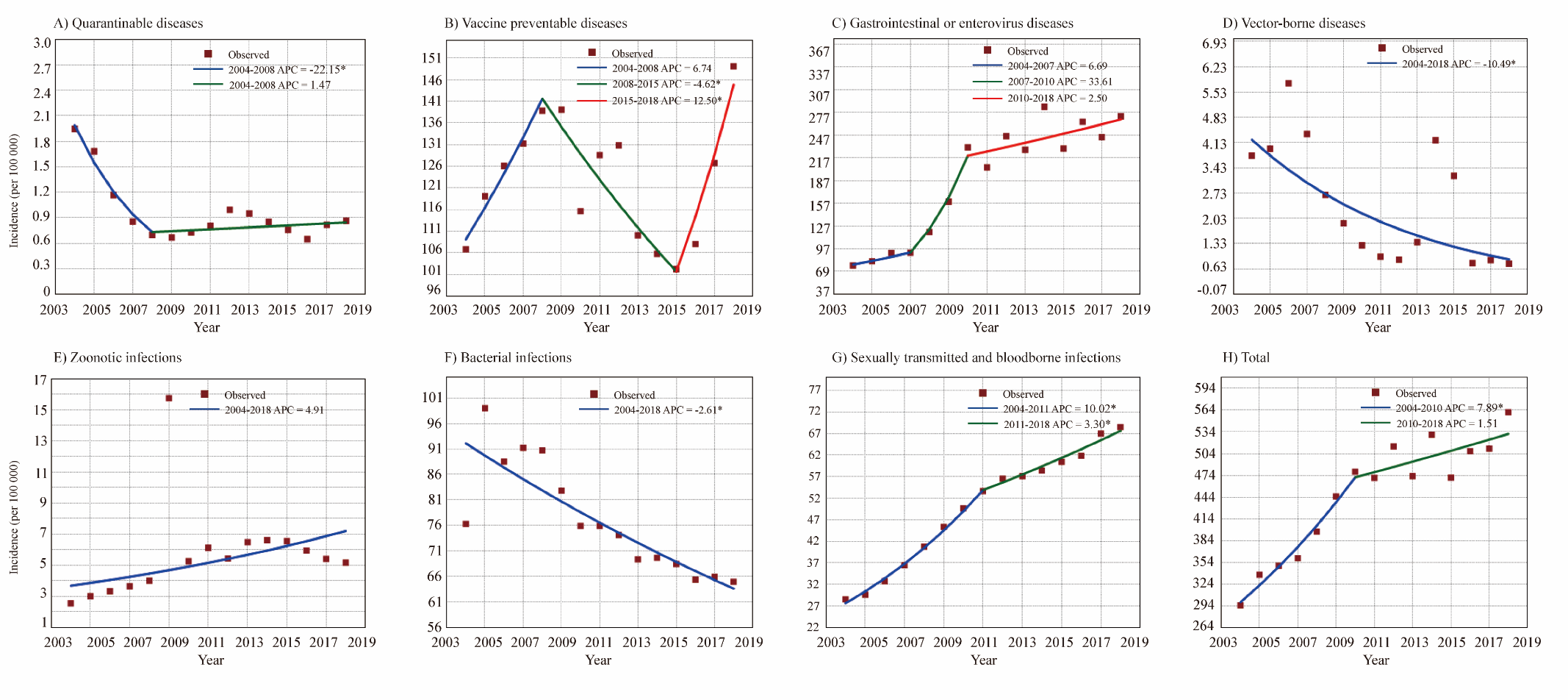


**Fig S1. The annual percentage changes (APC) and turning point in the trend from 2004 to 2018 for total and seven categories of infectious diseases using the joinpoint regression models**

Note: * indicated that APC was statistically significant during the study period. The red squares represent the annual observed incidence. If there is only one blue fitted trend line, it meant that there were no joinpoints years in the past 15 years. However, if there were two fitted trend lines with green and blue color, it meant that there was a joinpoints year during the 15 years using the Joinpoint regression model. If there are three fitted trend lines with green, blue and red color, it meant that there was two joinpoints years in the 15 years using the Joinpoint regression model. * indicated a statistically significant trend.

**Fig S2. Ranks of mortality or number of deaths for 44 notifiable infectious diseases during the past 15-years from 2004 to 2018.**


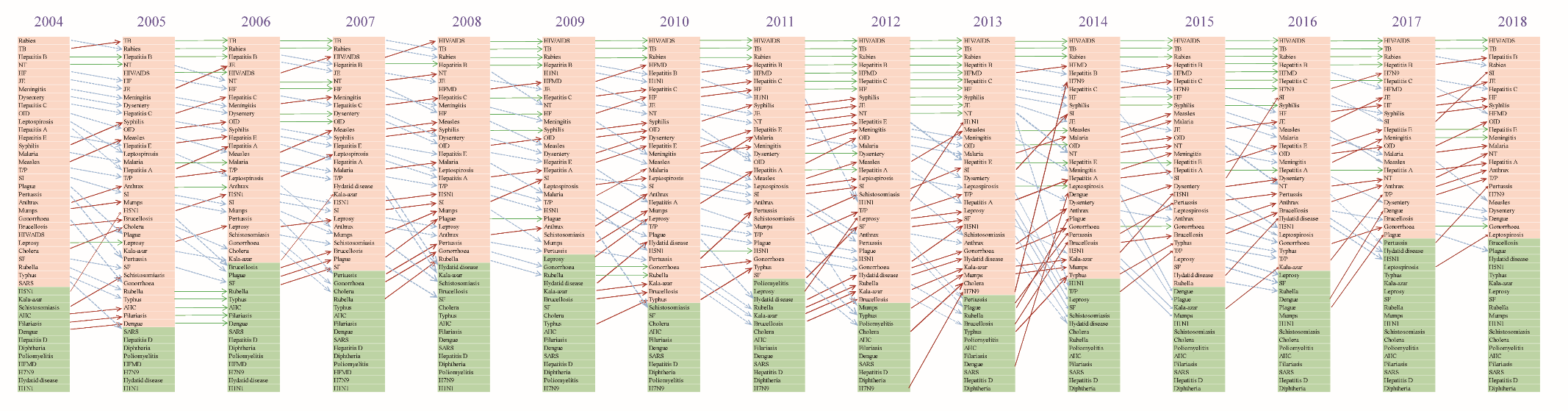


Note: Ranking is based on the incidence of each infectious disease. Abbreviations were showed as follows: TB, tuberculosis; OID, infectious diarrhoeal diseases other than cholera, bacterial dysentery, amoebic dysentery, typhoid, and paratyphoid; HFMD, Hand Foot Mouth Disease; SI, seasonal influenza; SF, scarlet fever; AHC, acute hemorrhagic conjunctivitis; T/P, typhoid and paratyphoid; JE, Japanese encephalitis; AIDS, acquired immune deficiency syndrome; HF, hemorrhagic fever; meningitis, meningococcal meningitis; NT, neonatal tetanus; H5N1, avian influenza H5N1; H7N9, avian influenza H7N9; H1N1, influenza A H1N1; SARS, severe acute respiratory syndrome. Green means no cases of infectious disease.

**Fig S3. The trends of incidence of each infectious disease in both genders and its joinpoint(s) during the past 15-years from 2004 to 2018**


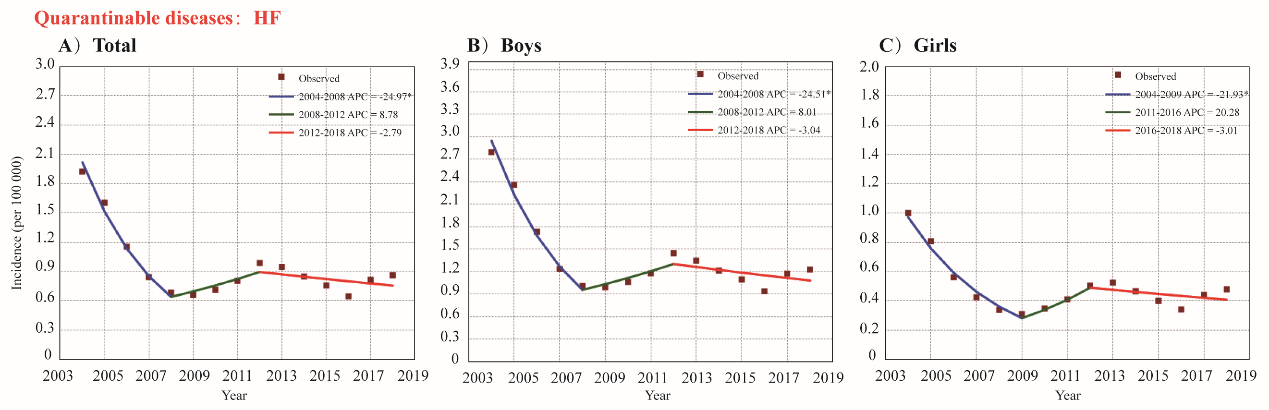


**Fig S3-1. The trends of incidence of Haemorrhagic Fever in both genders and its joinpoint(s) during the past 15-years from 2004 to 2018 (* represented the statistical significant trends. Legends were the same to Fig S1)**

**
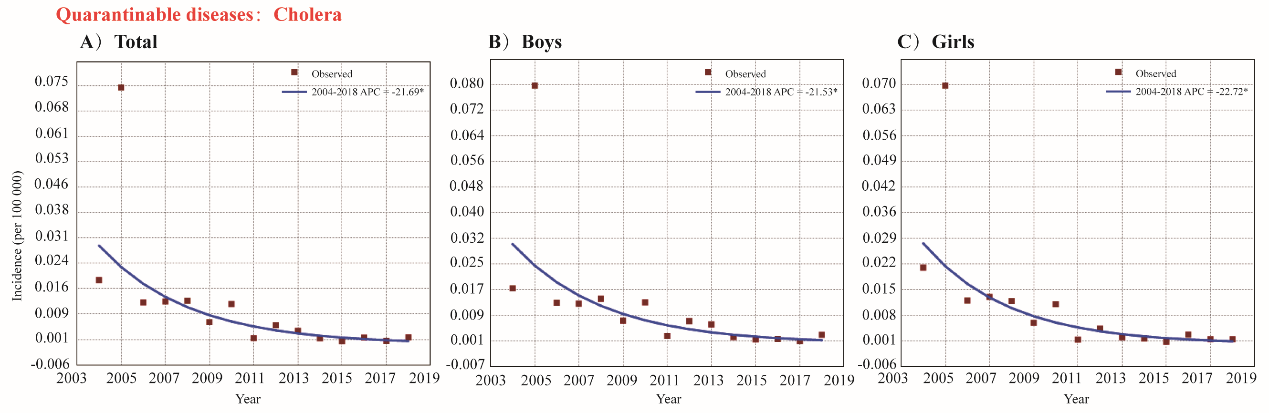
**

**Fig S3-2. The trends of incidence of Cholera in both genders and its joinpoint(s) during the past 15-years from 2004 to 2018 (* represented the statistical significant trends. Legends were the same to Fig S1)**

**
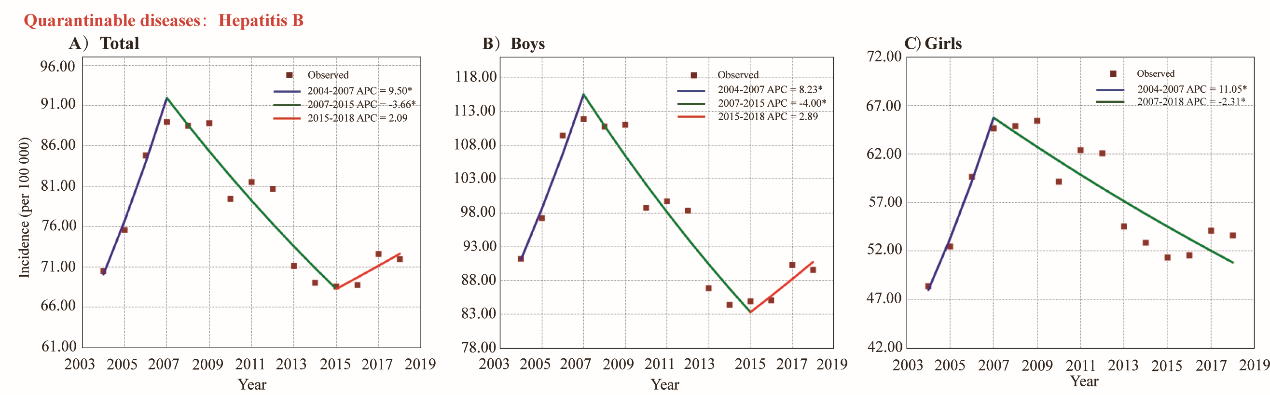
**

**Fig S3-3. The trends of incidence of Hepatitis B in both genders and its joinpoint(s) during the past 15-years from 2004 to 2018 (* represented the statistical significant trends. Legends were the same to Fig S1)**

**
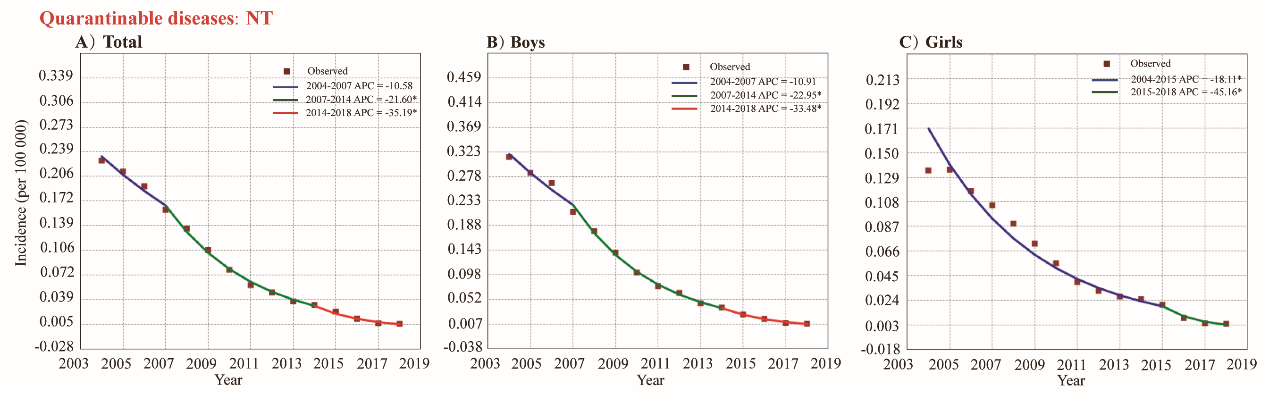
**

**Fig S3-4. The trends of incidence of NT in both genders and its joinpoint(s) during the past 15-years from 2004 to 2018 (* represented the statistical significant trends. Legends were the same to Fig S1)**

**
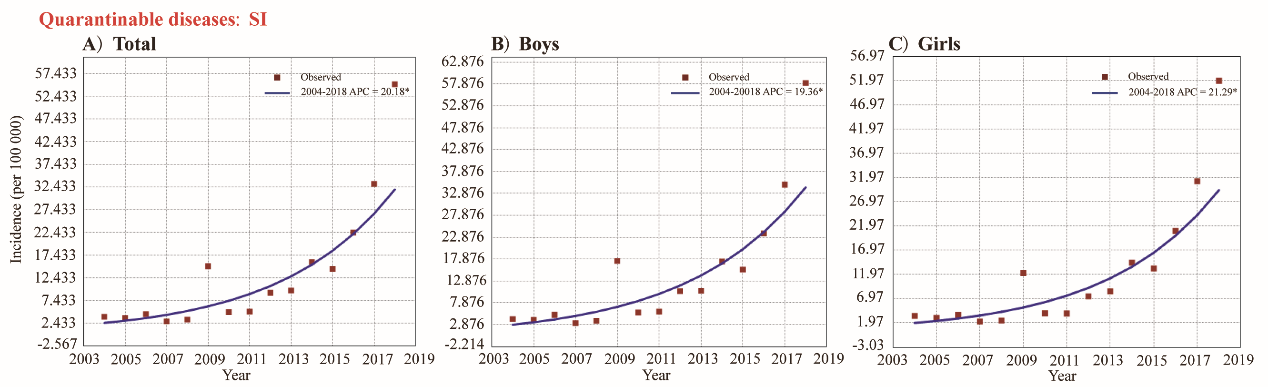
**

**Fig S3-5. The trends of incidence of SI in both genders and its joinpoint(s) during the past 15-years from 2004 to 2018 (* represented the statistical significant trends. Legends were the same to Fig S1)**

**
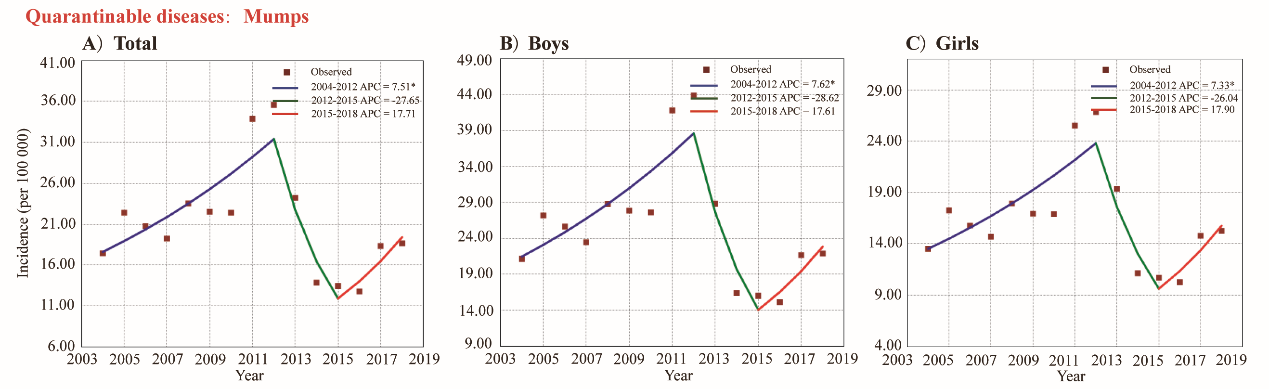
**

**Fig S3-6. The trends of incidence of Mumps in both genders and its joinpoint(s) during the past 15-years from 2004 to 2018 (* represented the statistical significant trends. Legends were the same to Fig S1)**

**
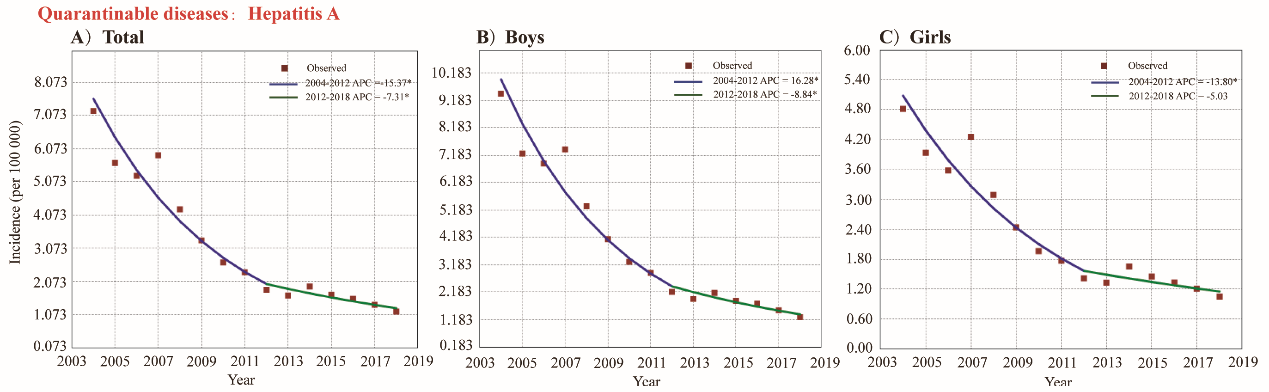
**

**Fig S3-7. The trends of incidence of Hepatitis A in both genders and its joinpoint(s) during the past 15-years from 2004 to 2018 (* represented the statistical significant trends. Legends were the same to Fig S1)**

**
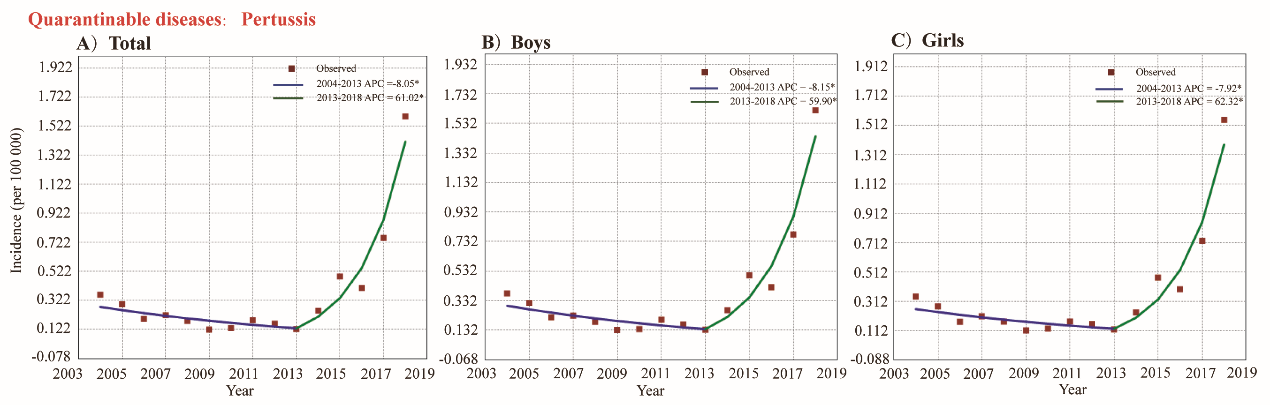
**

**Fig S3-8. The trends of incidence of Pertussis in both genders and its joinpoint(s) during the past 15-years from 2004 to 2018 (* represented the statistical significant trends. Legends were the same to Fig S1)**

**
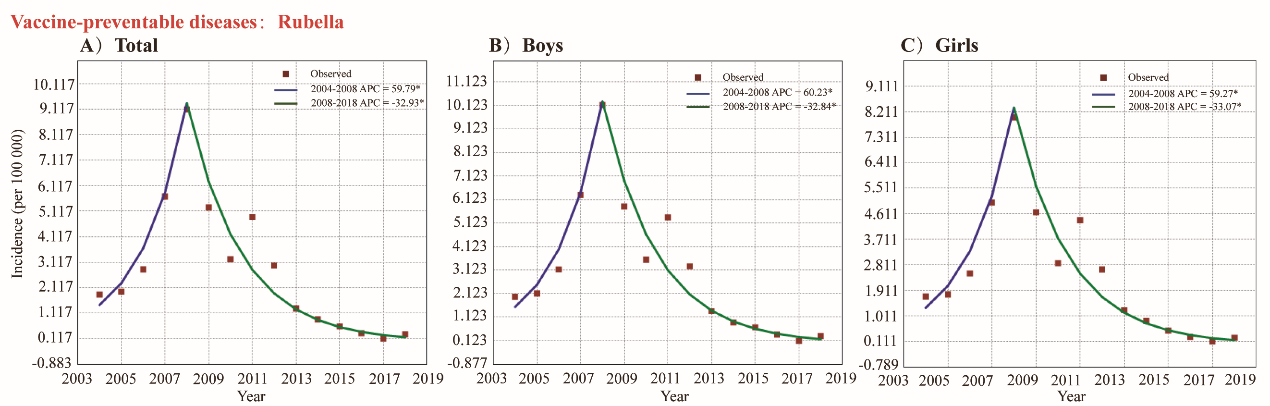
**

**Fig S3-9. The trends of incidence of Rubella in both genders and its joinpoint(s) during the past 15-years from 2004 to 2018 (* represented the statistical significant trends. Legends were the same to Fig S1)**

**
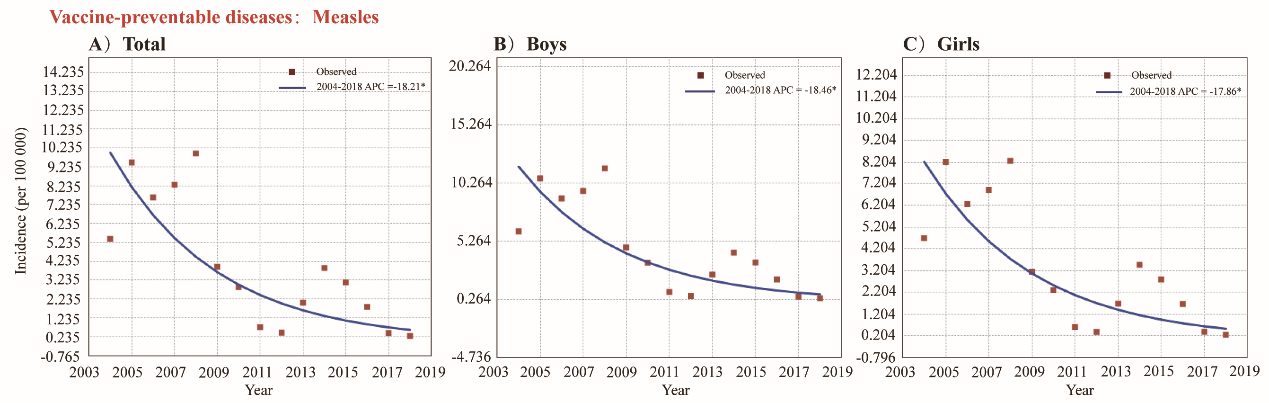
**

**Fig S3-10. The trends of incidence of Measles in both genders and its joinpoint(s) during the past 15-years from 2004 to 2018 (* represented the statistical significant trends. Legends were the same to Fig S1)**

**
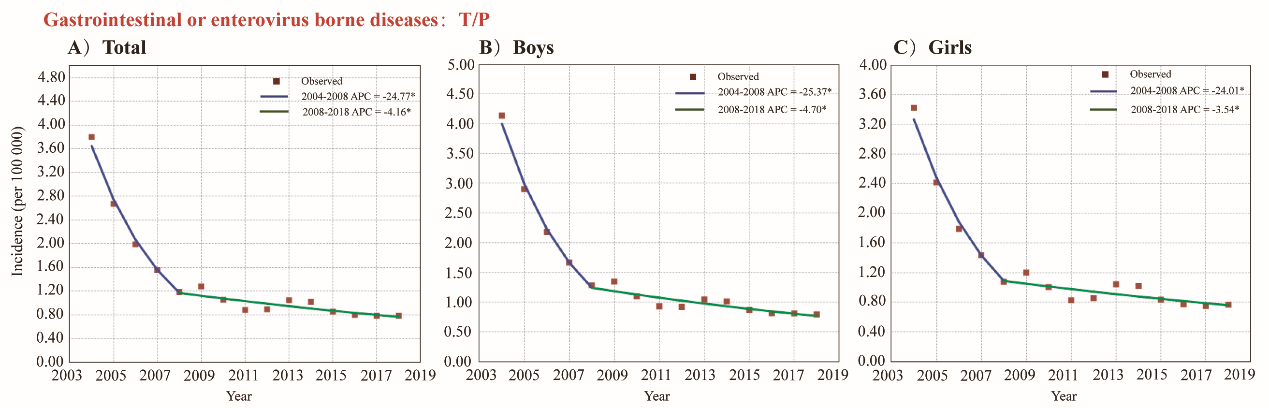
**

**Fig S3-11. The trends of incidence of T/P in both genders and its joinpoint(s) during the past 15-years from 2004 to 2018 (* represented the statistical significant trends. Legends were the same to Fig S1)**

**
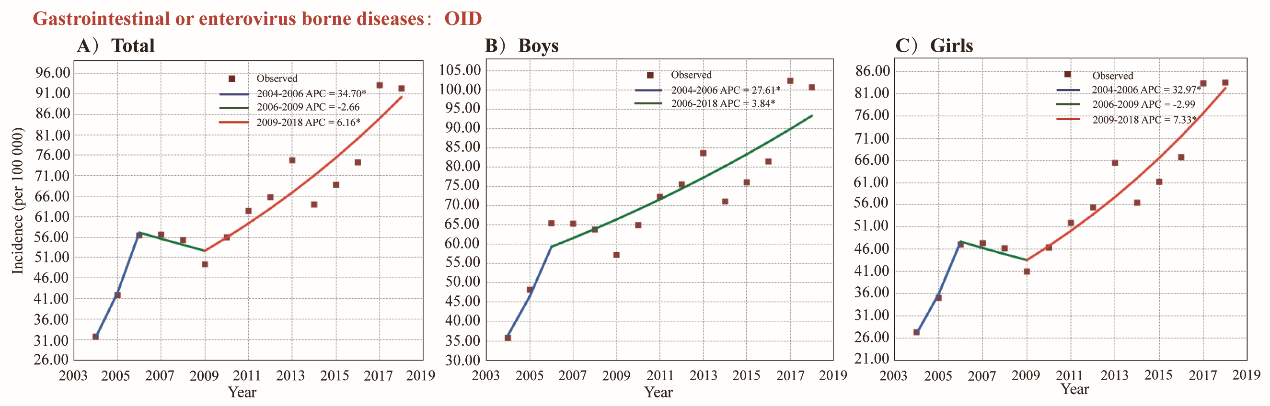
**

**Fig S3-12. The trends of incidence of OID in both genders and its joinpoint(s) during the past 15-years from 2004 to 2018 (* represented the statistical significant trends. Legends were the same to Fig S1)**

**
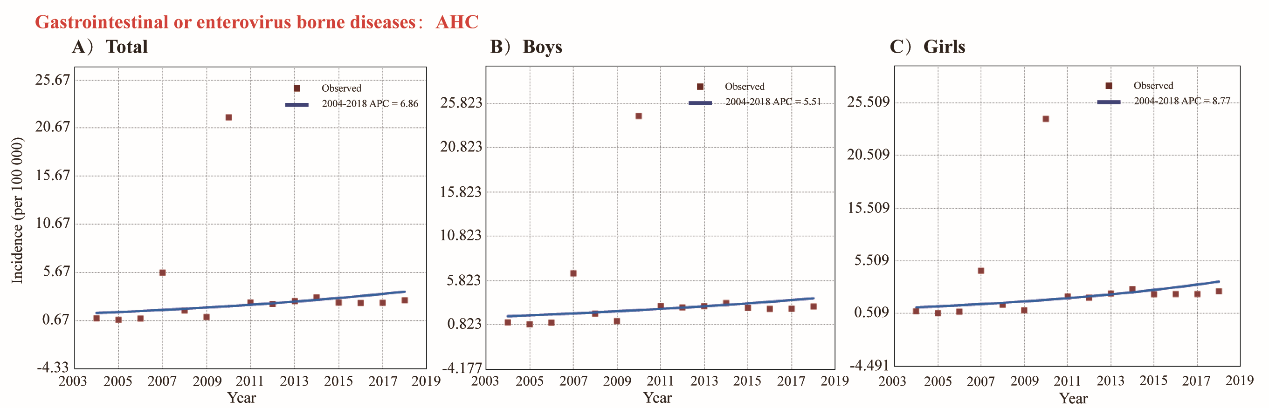
**

**Fig S3-13. The trends of incidence of AHC in both genders and its joinpoint(s) during the past 15-years from 2004 to 2018 (* represented the statistical significant trends. Legends were the same to Fig S1)**

**
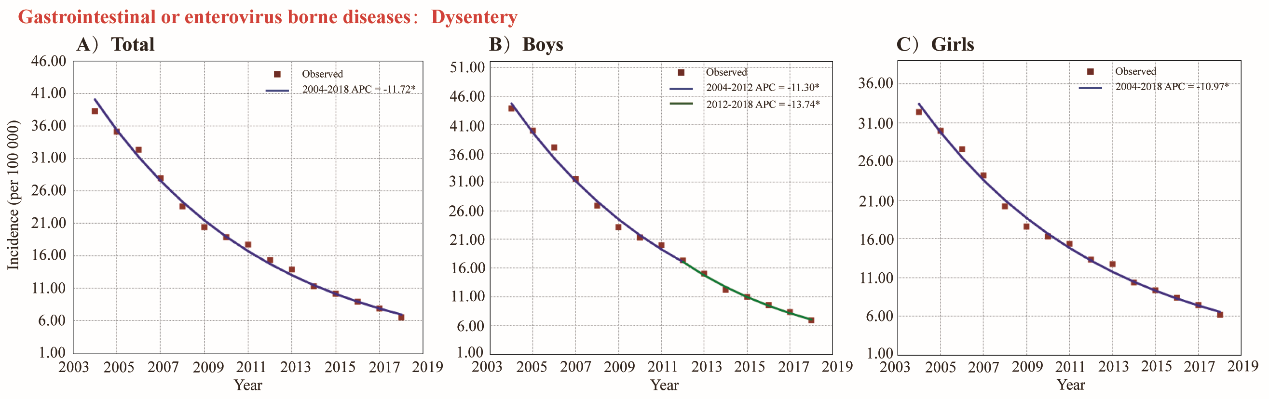
**

**Fig S3-14. The trends of incidence of Dysentery in both genders and its joinpoint(s) during the past 15-years from 2004 to 2018 (* represented the statistical significant trends. Legends were the same to Fig S1)**

**
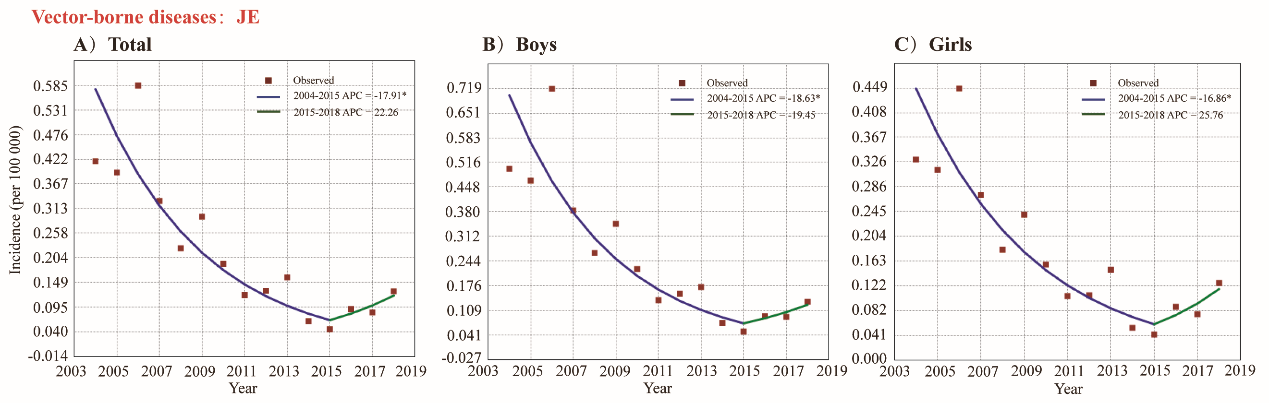
**

**Fig S3-15. The trends of incidence of JE in both genders and its joinpoint(s) during the past 15-years from 2004 to 2018 (* represented the statistical significant trends. Legends were the same to Fig S1)**

**
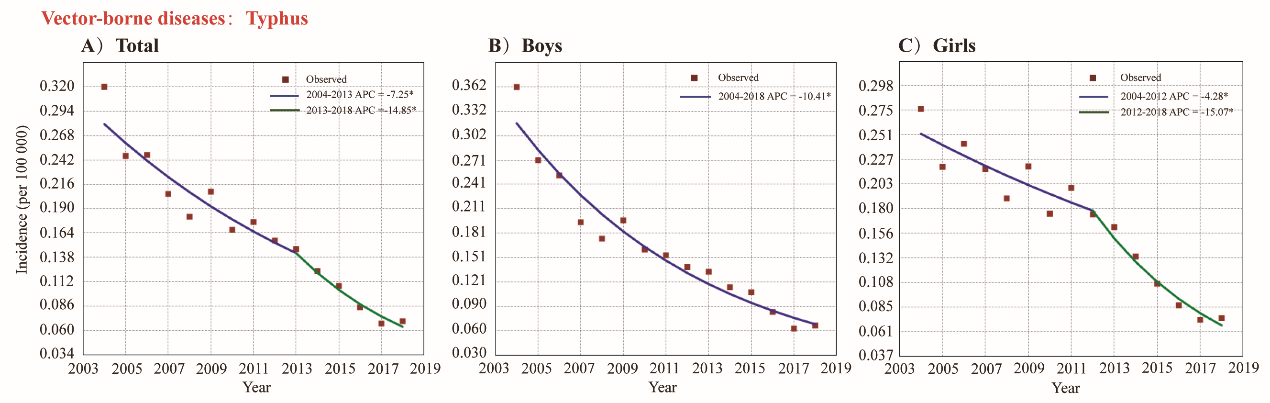
**

**Fig S3-16. The trends of incidence of Typhus in both genders and its joinpoint(s) during the past 15-years from 2004 to 2018 (* represented the statistical significant trends. Legends were the same to Fig S1)**

**
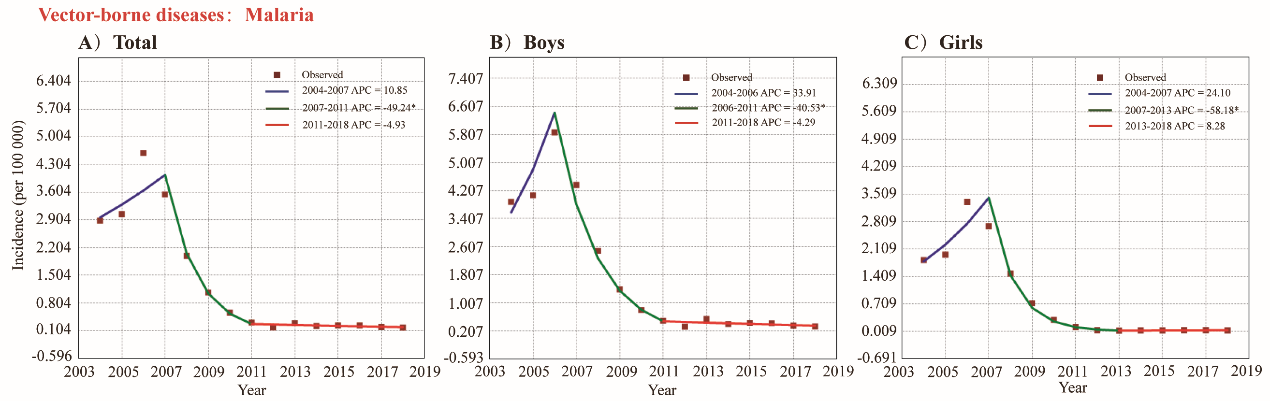
**

**Fig S3-17. The trends of incidence of Malaria in both genders and its joinpoint(s) during the past 15-years from 2004 to 2018 (* represented the statistical significant trends. Legends were the same to Fig S1)**

**
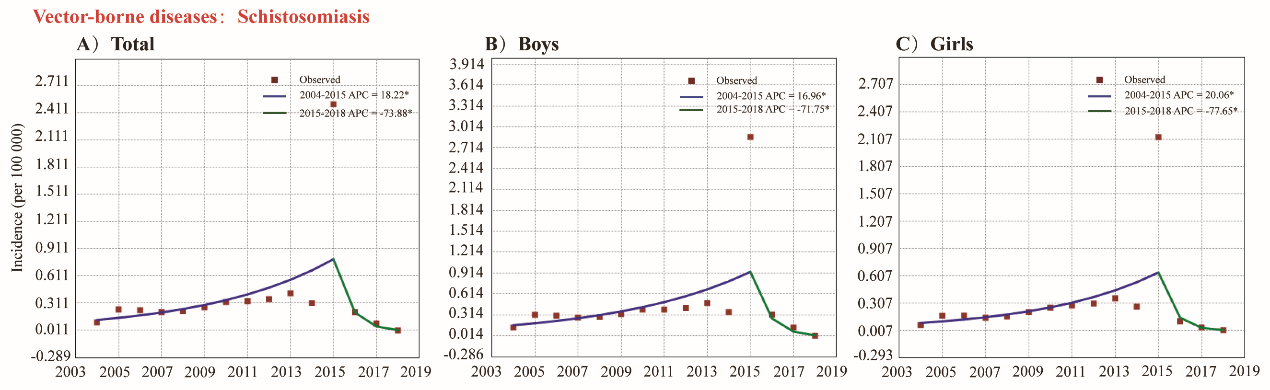
**

**Fig S3-18. The trends of incidence of Schistosomiasis in both genders and its joinpoint(s) during the past 15-years from 2004 to 2018 (* represented the statistical significant trends. Legends were the same to Fig S1)**

**
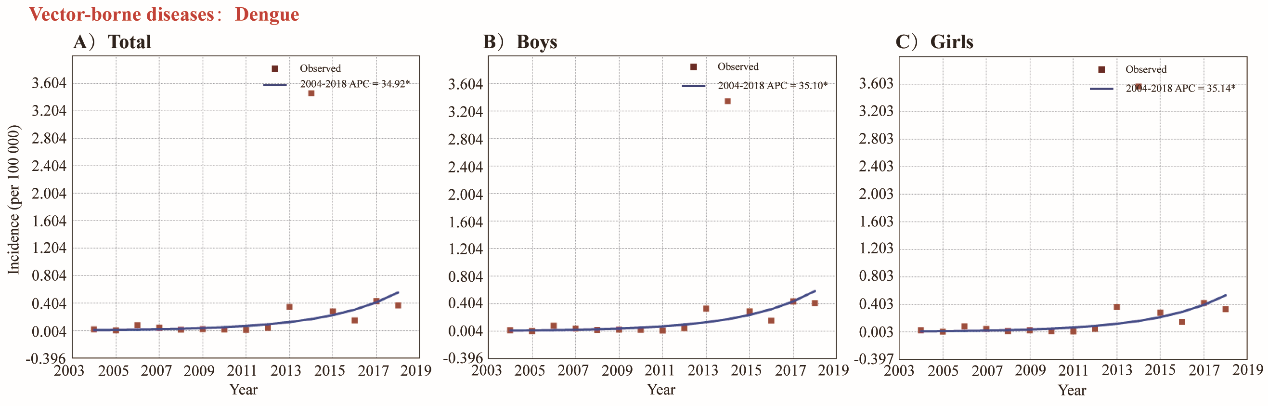
**

**Fig S3-19. The trends of incidence of Dengue in both genders and its joinpoint(s) during the past 15-years from 2004 to 2018 (* represented the statistical significant trends. Legends were the same to Fig S1)**

**
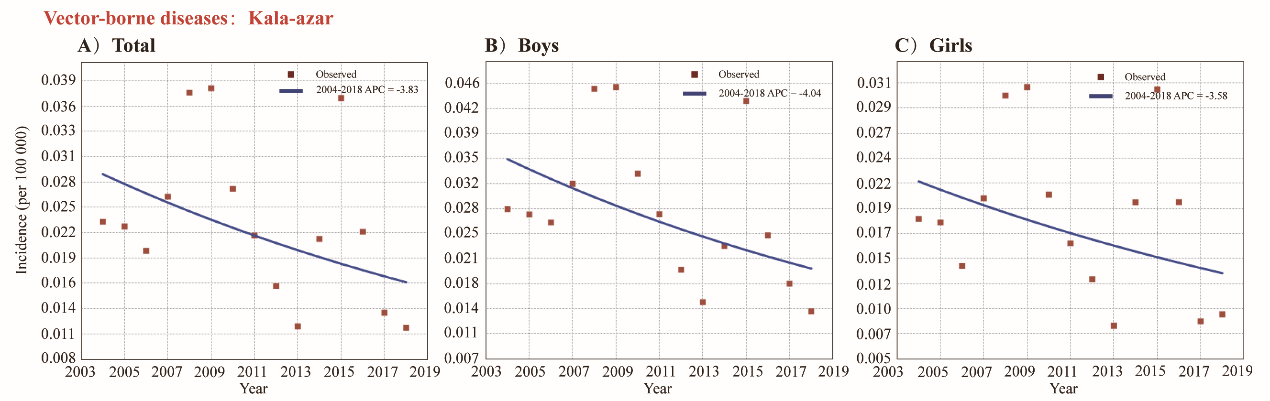
**

**Fig S3-20. The trends of incidence of Kala-azar in both genders and its joinpoint(s) during the past 15-years from 2004 to 2018 (* represented the statistical significant trends. Legends were the same to Fig S1)**

**
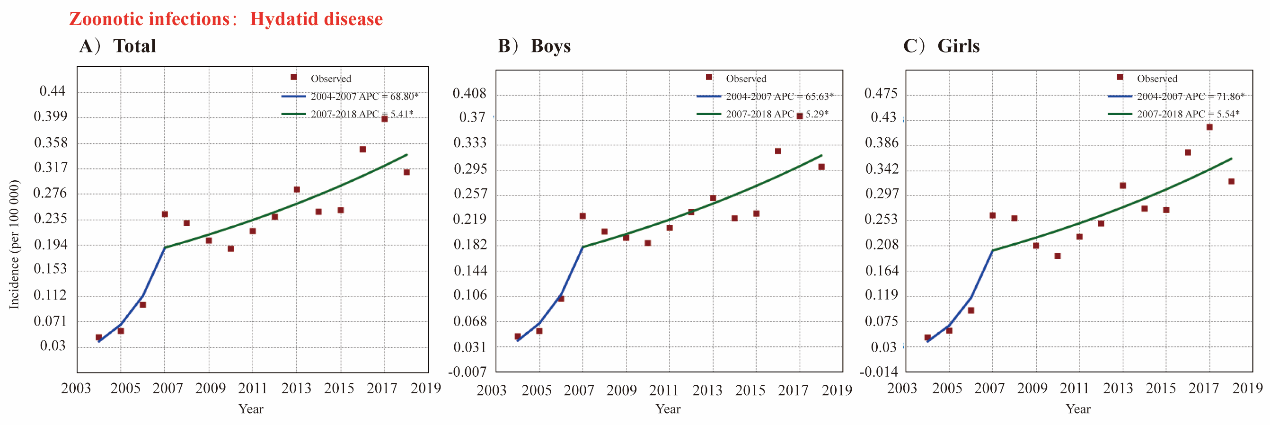
**

**Fig S3-21. The trends of incidence of Hydatid disease in both genders and its joinpoint(s) during the past 15-years from 2004 to 2018 (* represented the statistical significant trends. Legends were the same to Fig S1)**

**
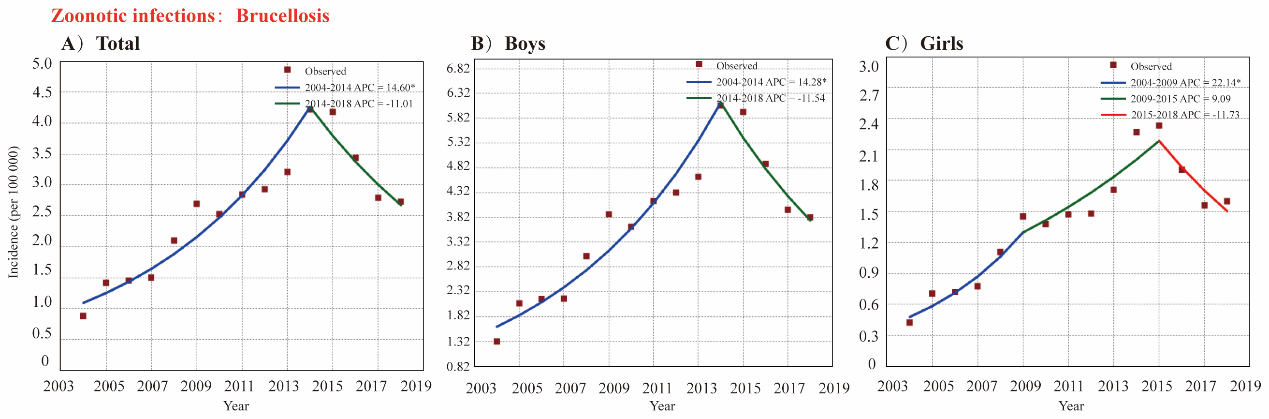
**

**Fig S3-22. The trends of incidence of Brucellosis in both genders and its joinpoint(s) during the past 15-years from 2004 to 2018 (* represented the statistical significant trends. Legends were the same to Fig S1)**


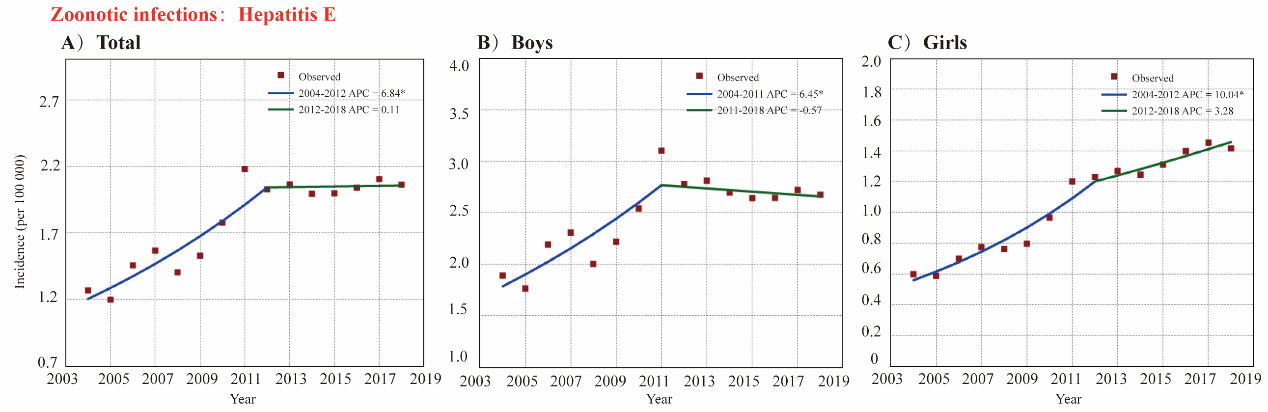


**Fig S3-23. The trends of incidence of Hepatitis E in both genders and its joinpoint(s) during the past 15-years from 2004 to 2018 (* represented the statistical significant trends. Legends were the same to Fig S1)**


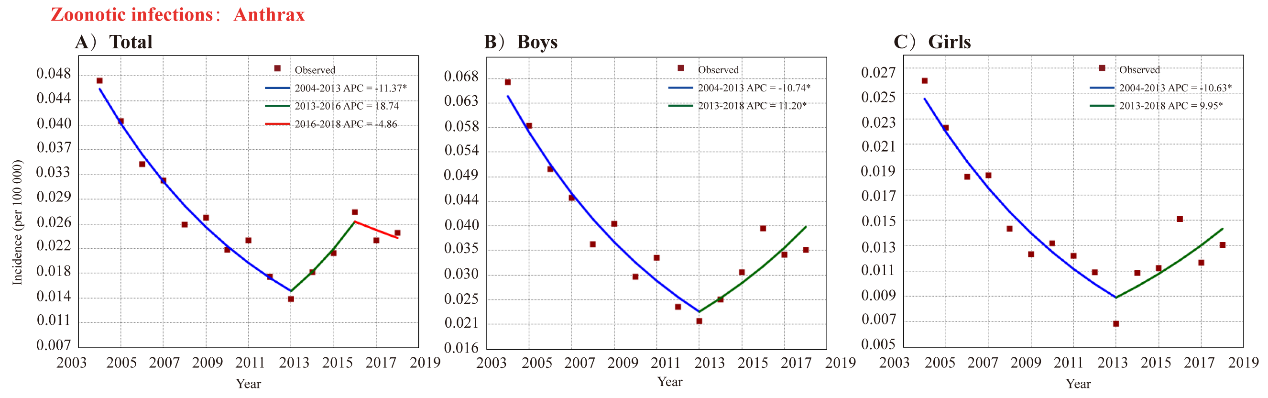


**Fig S3-24. The trends of incidence of Anthrax in both genders and its joinpoint(s) during the past 15-years from 2004 to 2018 (* represented the statistical significant trends. Legends were the same to Fig S1)**


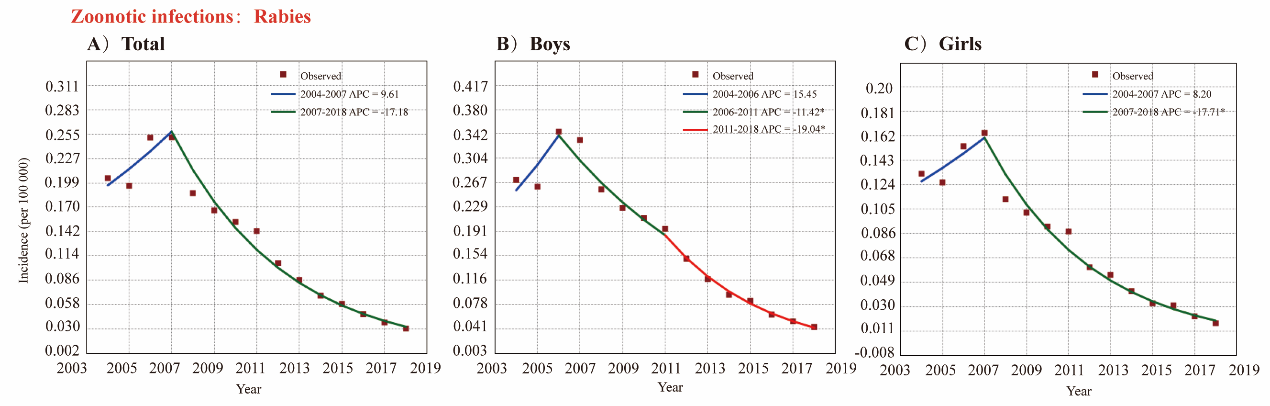


**Fig S3-25. The trends of incidence of Rabies in both genders and its joinpoint(s) during the past 15-years from 2004 to 2018 (* represented the statistical significant trends. Legends were the same to Fig S1)**


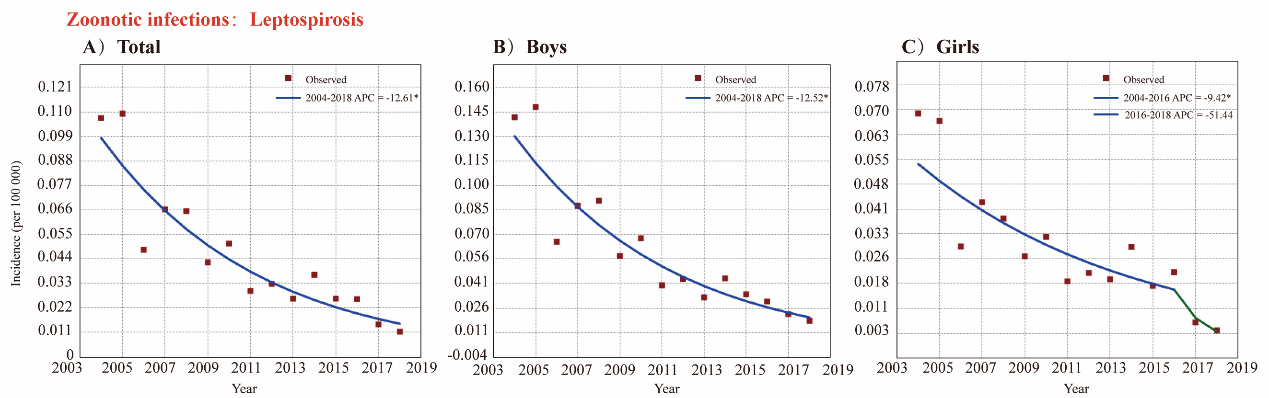


**Fig S3-26. The trends of incidence of Leptospirosis in both genders and its joinpoint(s) during the past 15-years from 2004 to 2018 (* represented the statistical significant trends. Legends were the same to Fig S1)**

**
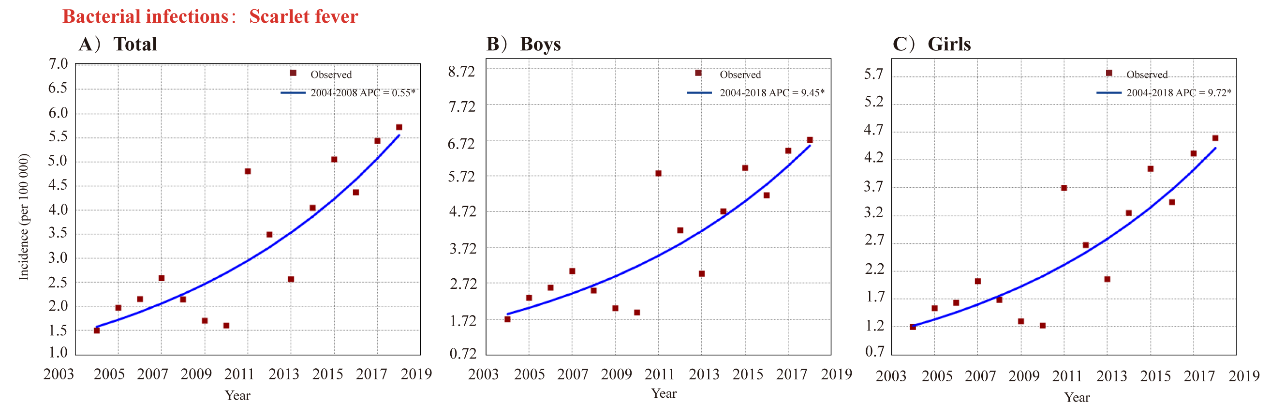
**

**Fig S3-27. The trends of incidence of Scarlet fever in both genders and its joinpoint(s) during the past 15-years from 2004 to 2018 (* represented the statistical significant trends. Legends were the same to Fig S1)**

**
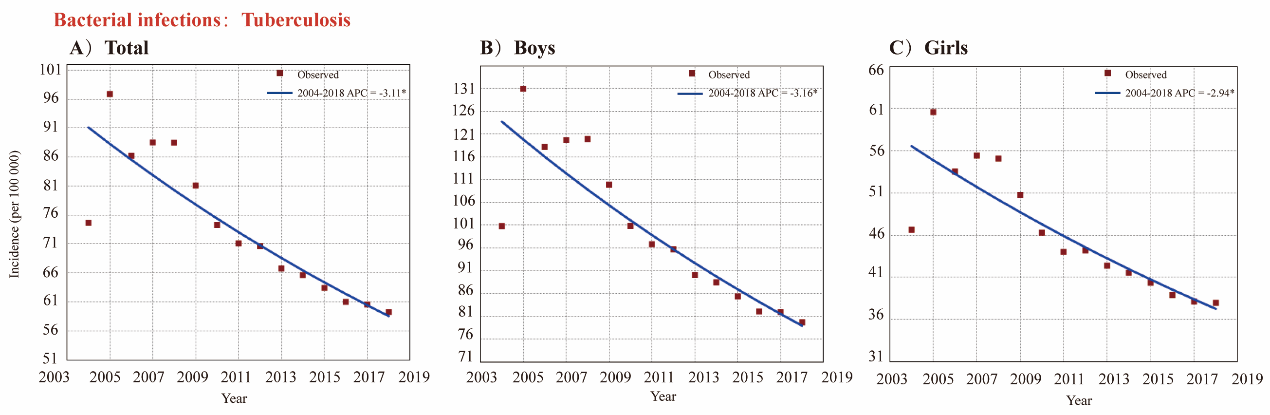
**

**Fig S3-28. The trends of incidence of Tuberculosis in both genders and its joinpoint(s) during the past 15-years from 2004 to 2018 (* represented the statistical significant trends. Legends were the same to Fig S1)**

**
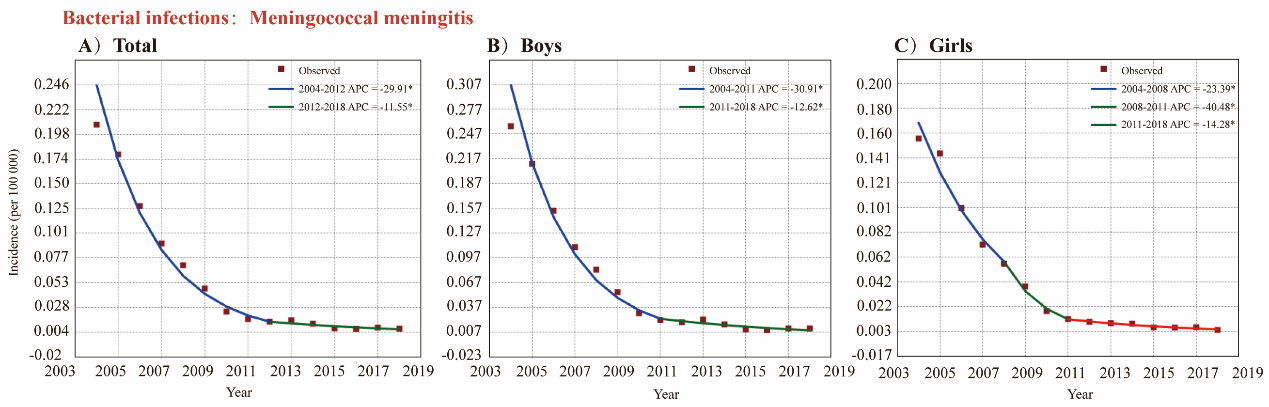
**

**Fig S3-29. The trends of incidence of Meningococcal meningitis in both genders and its joinpoint(s) during the past 15-years from 2004 to 2018 (* represented the statistical significant trends. Legends were the same to Fig S1)**

**
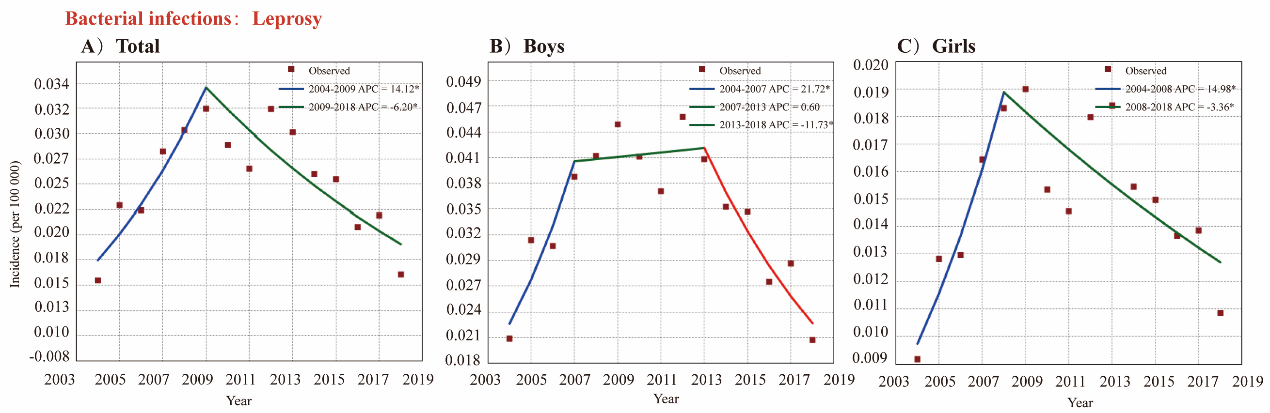
**

**Fig S3-30. The trends of incidence of Leprosy in both genders and its joinpoint(s) during the past 15-years from 2004 to 2018 (* represented the statistical significant trends. Legends were the same to Fig S1)**


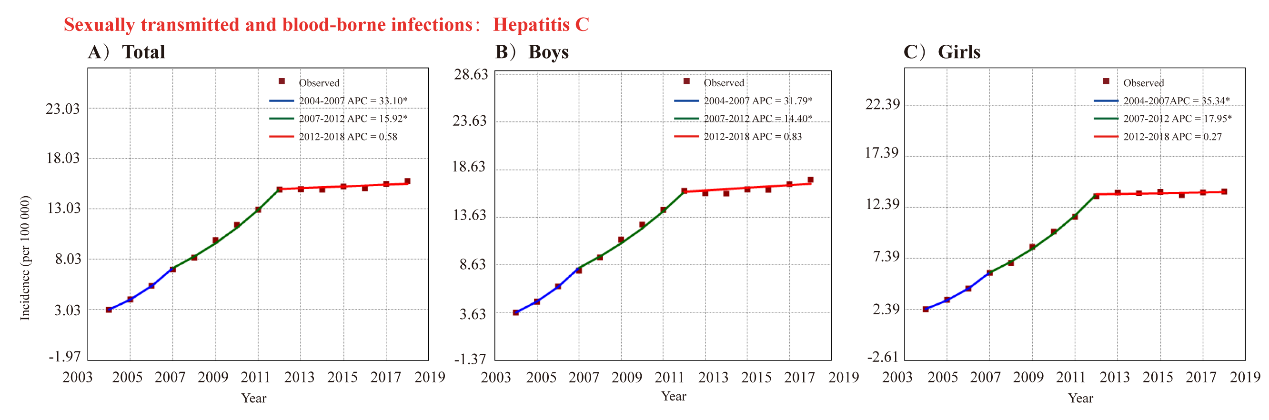


**Fig S3-31. The trends of incidence of Hepatitis C in both genders and its joinpoint(s) during the past 15-years from 2004 to 2018 (* represented the statistical significant trends. Legends were the same to Fig S1)**


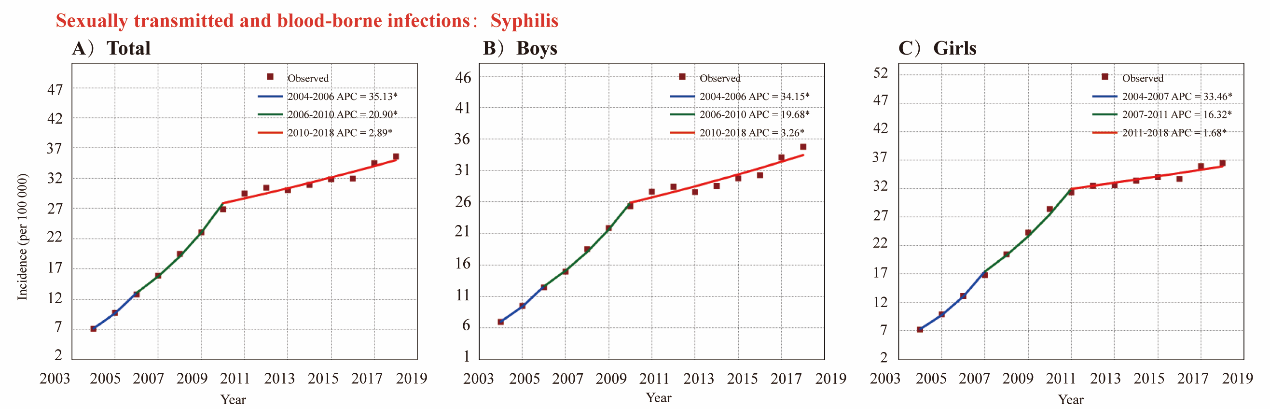


**Fig S3-32. The trends of incidence of Syphilis in both genders and its joinpoint(s) during the past 15-years from 2004 to 2018 (* represented the statistical significant trends. Legends were the same to Fig S1)**


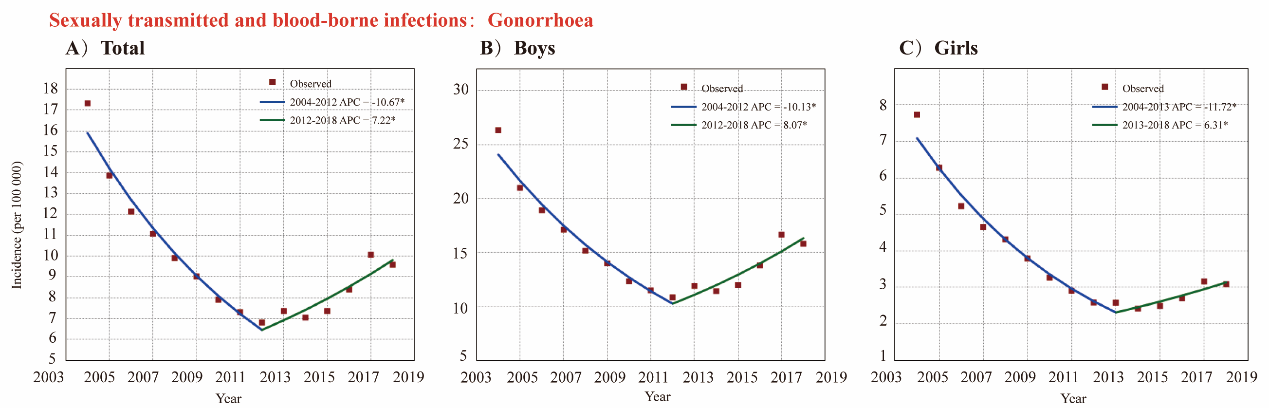


**Fig S3-33. The trends of incidence of Gonorrhoea in both genders and its joinpoint(s) during the past 15-years from 2004 to 2018 (* represented the statistical significant trends. Legends were the same to Fig S1)**


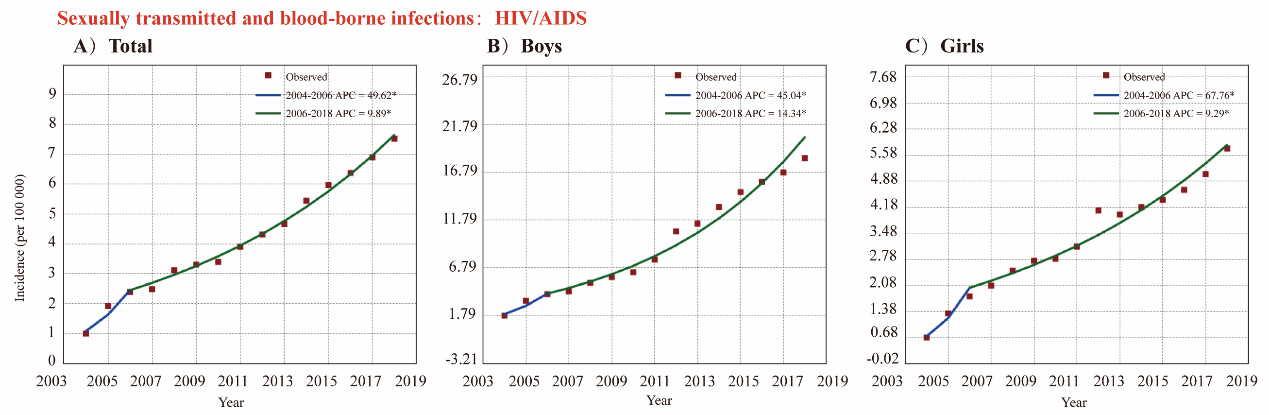


**Fig S3-34. The trends of incidence of HIV/AIDS in both genders and its joinpoint(s) during the past 15-years from 2004 to 2018 (* represented the statistical significant trends. Legends were the same to Fig S1)**

**Fig S4. The comparison of incidence of 44 current notifiable infectious diseases in boys or males and girls or females from 2004 to 2018 (using the chi-square test)**

**
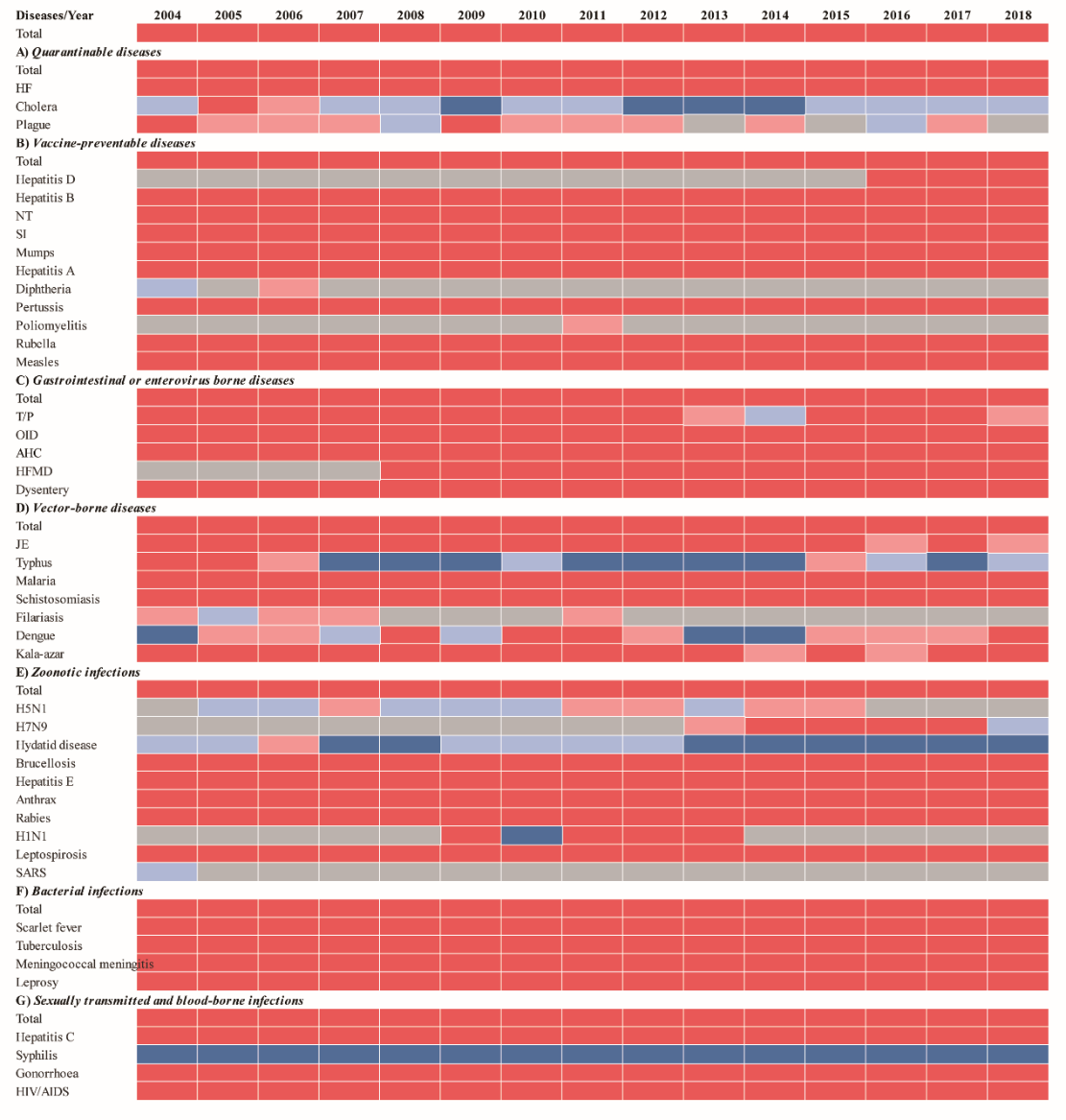
**

Note: The dark red boxes indicated that there were statistical significant difference in the incidence rate between male and female, the incidence rate of male was higher than that of female, and the P value was less than 0.05; the dark blue boxes indicated that there was a statistical difference between the two genders, and the incidence rate of female was higher than that of male, and the P value is less than 0.05; The light red boxes indicated that there was no statistical difference between the genders, and the incidence of male was higher than that of female, and the P value was equal to or greater than 0.05; the light blue boxes indicated that there was no statistical difference between the genders, and the incidence of female was higher than that of male, P-value equal to or greater than 0.05; grey boxes indicated cases without the disease during the survey year.

**Fig S5. Trends in age incidence rates for each infectious disease in females and males, from 2004 to 2018**

**
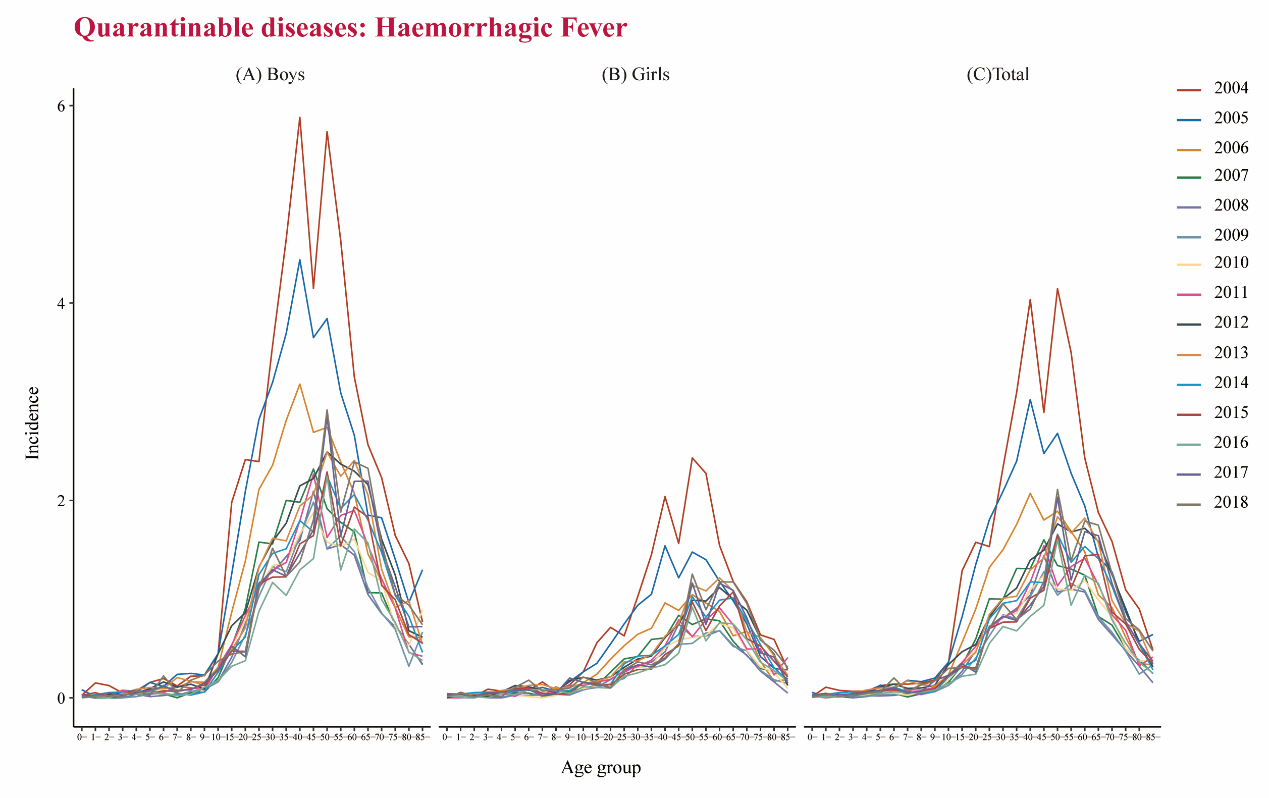
**

**Fig S5-1. Trends in age incidence rates for Haemorrhagic Fever, females and males, 2004-2018**

**
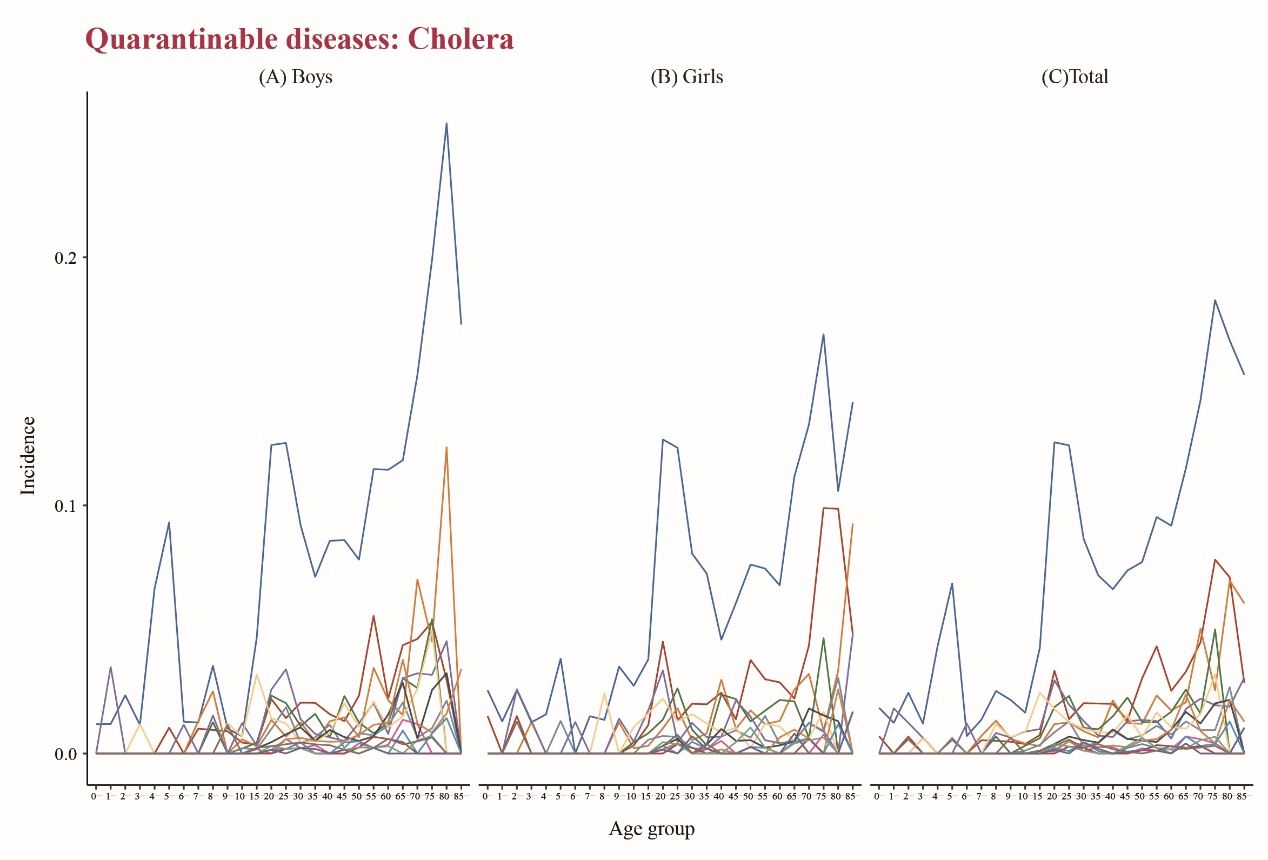
**

**Fig S5-2. Trends in age incidence rates for Cholera, females and males, 2004-2018 (Legends were the same to Fig S5-1)**


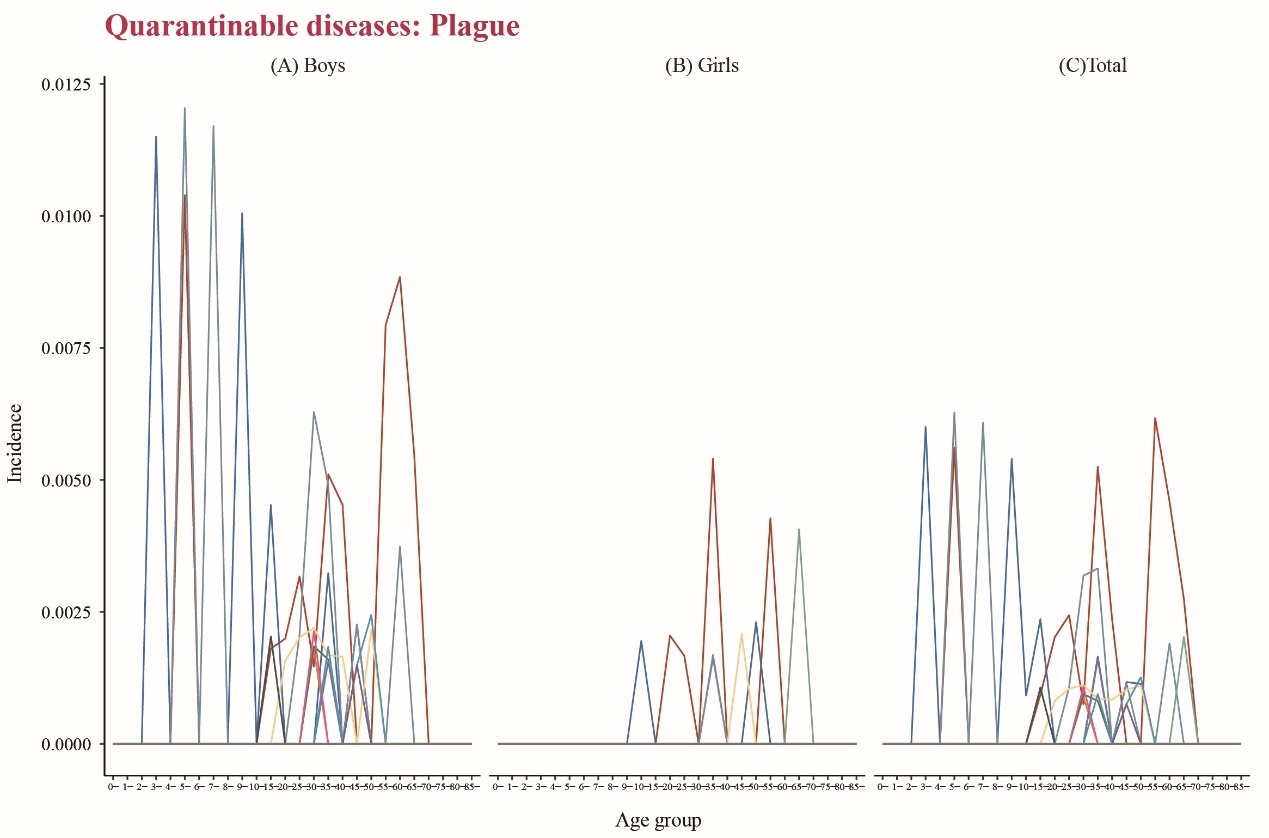


**Fig S5-3. Trends in age incidence rates for Plague, females and males, 2004-2018 (Legends were the same to Fig S5-1)**

**
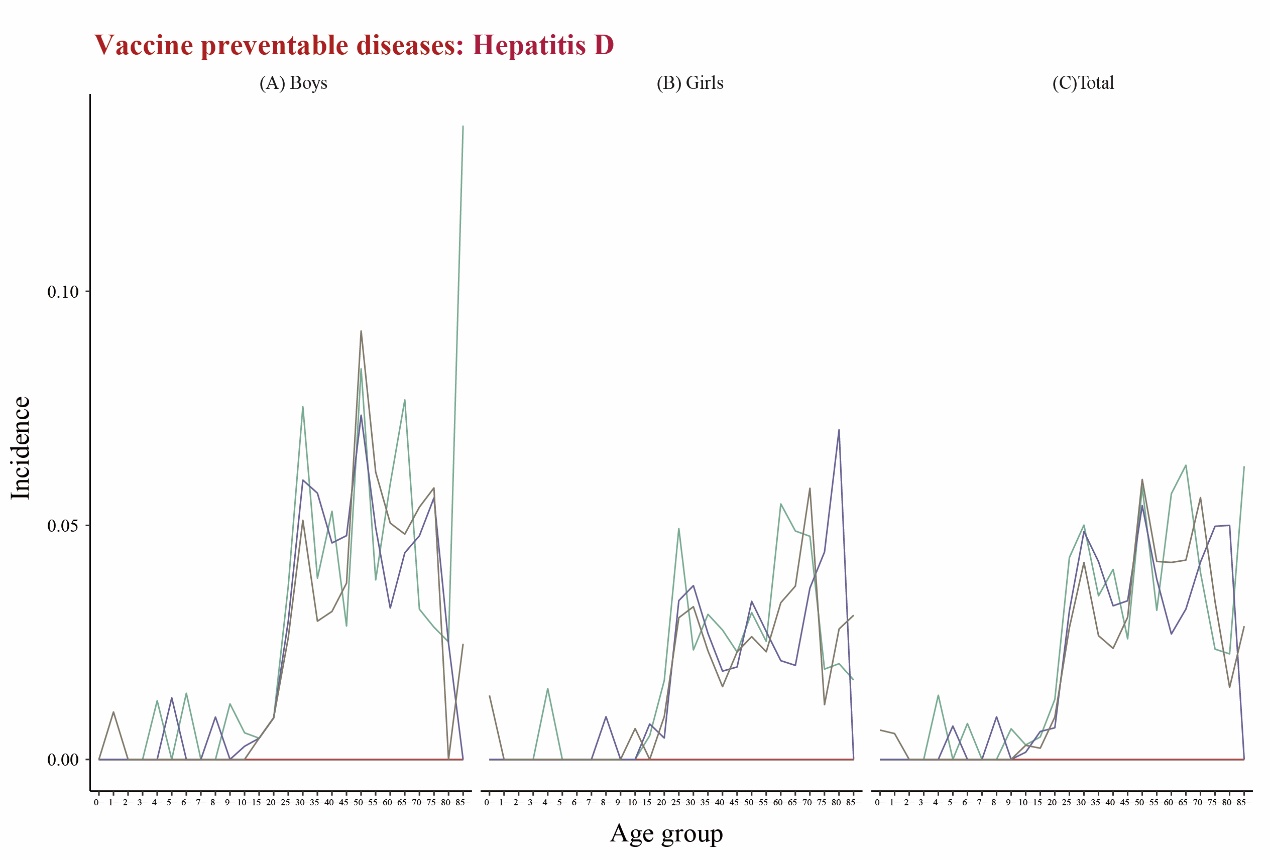
Fig S5-4. Trends in age incidence rates for Hepatitis D, females and males, 2004-2018 (Legends were the same to Fig S5-1)**


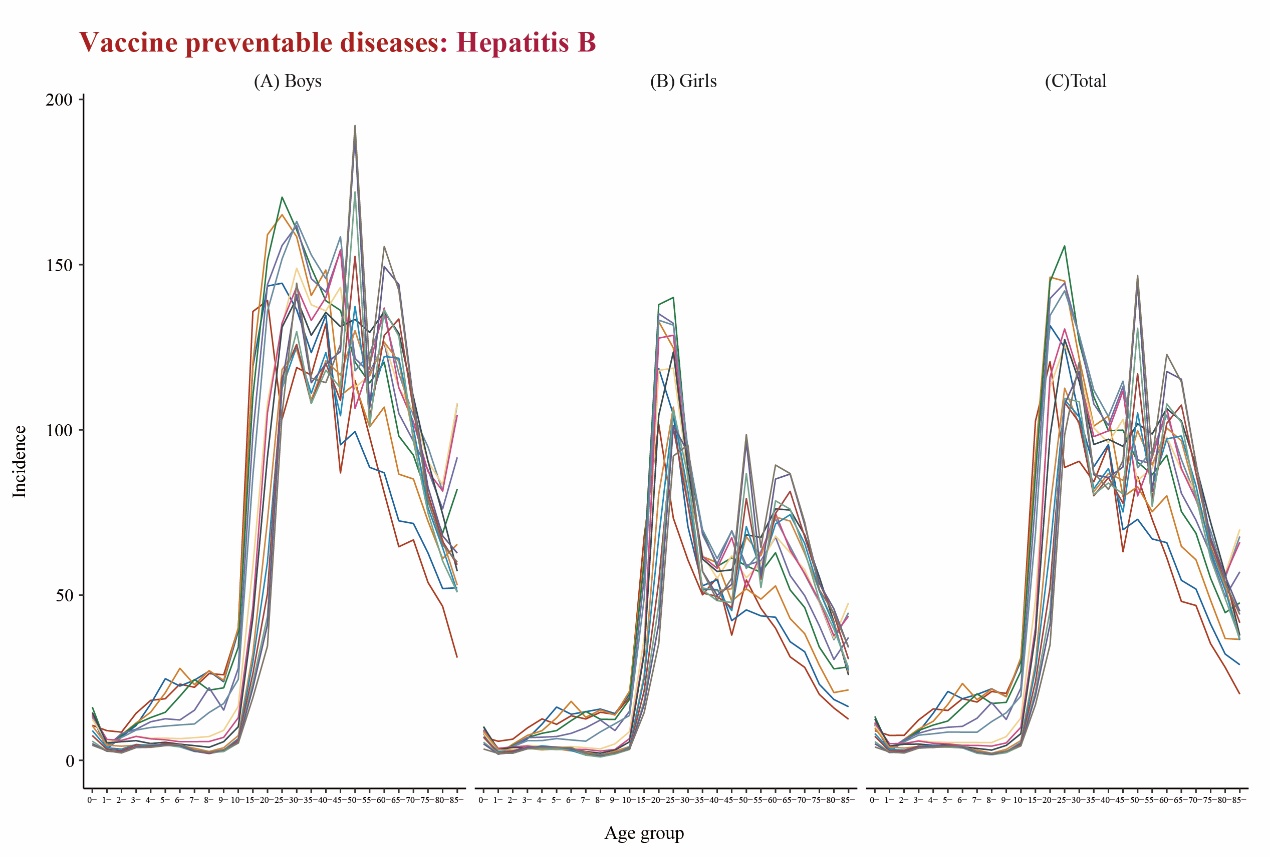


**Fig S5-5. Trends in age incidence rates for Hepatitis B, females and males, 2004-2018 (Legends were the same to Fig S5-1)**


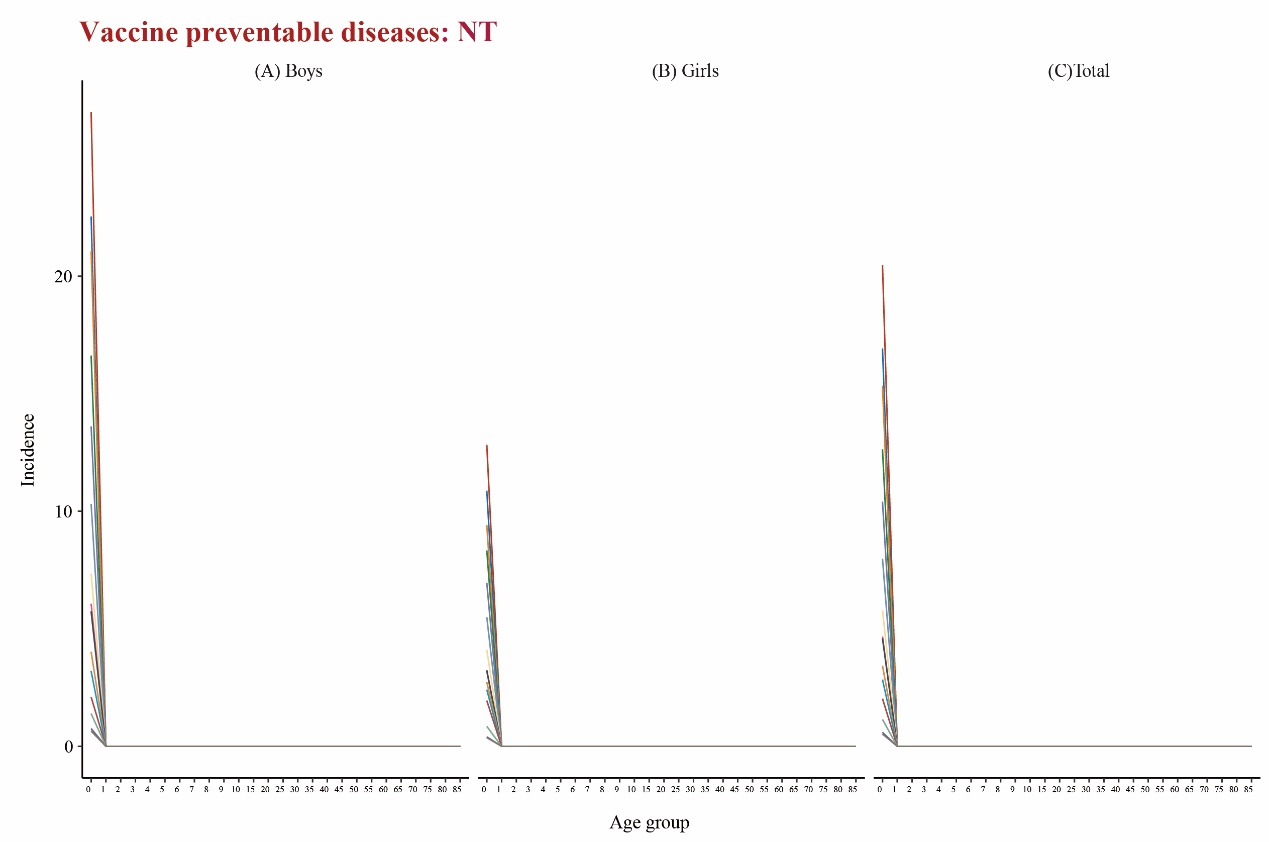


**Fig S5-6. Trends in age incidence rates for NT, females and males, 2004-2018 (Legends were the same to Fig S5-1)**


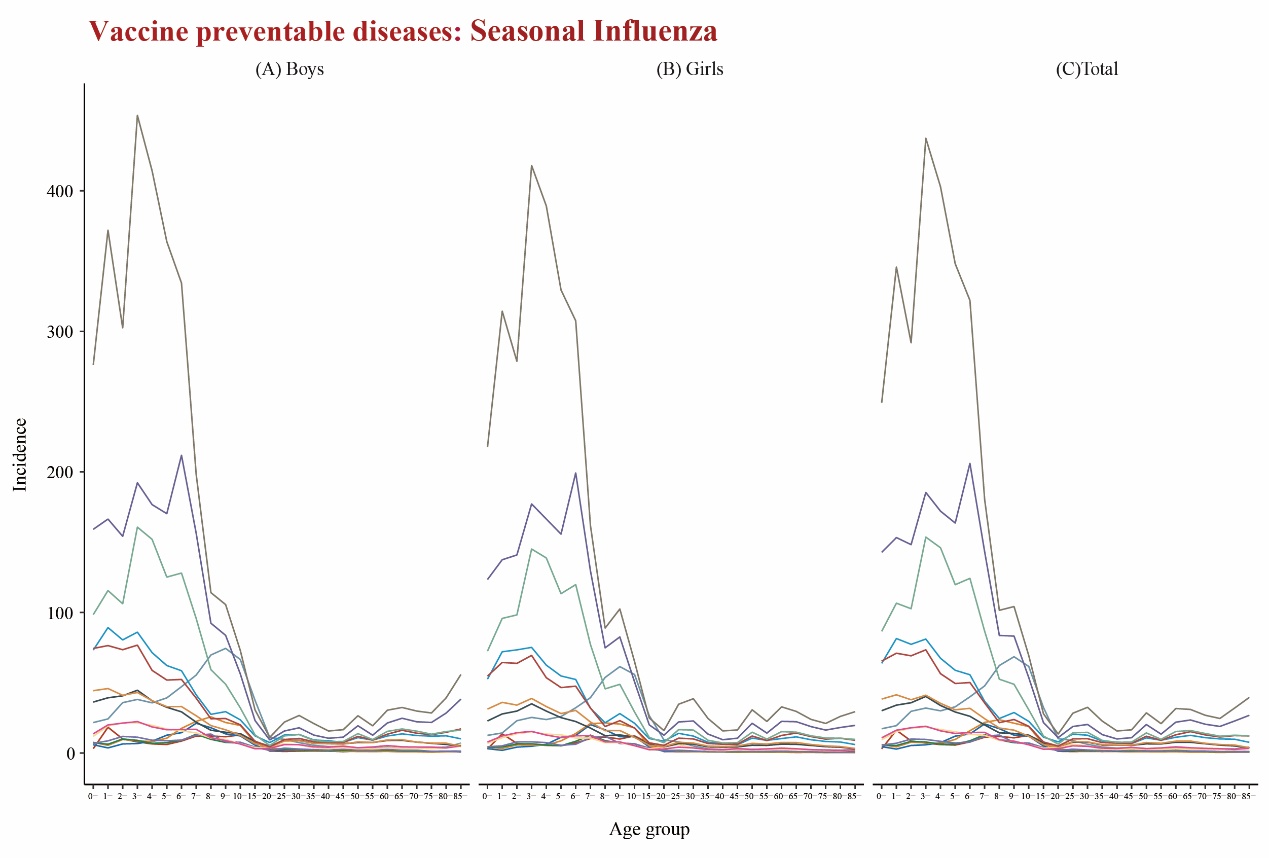


**Fig S5-7. Trends in age incidence rates for Seasonal Influenza, females and males, 2004-2018 (Legends were the same to Fig S5-1)**


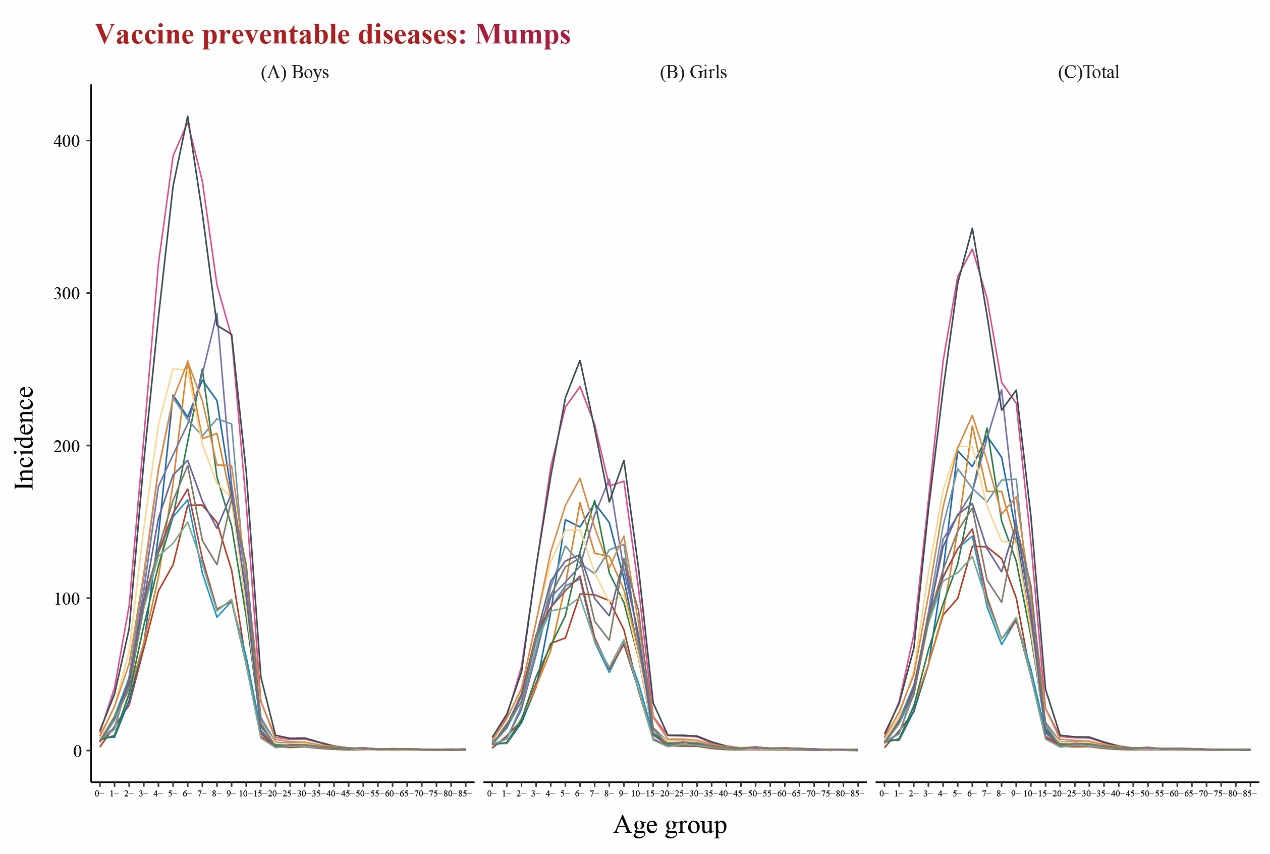


**Fig S5-8. Trends in age incidence rates for Mumps, females and males, 2004-2018 (Legends were the same to Fig S5-1)**


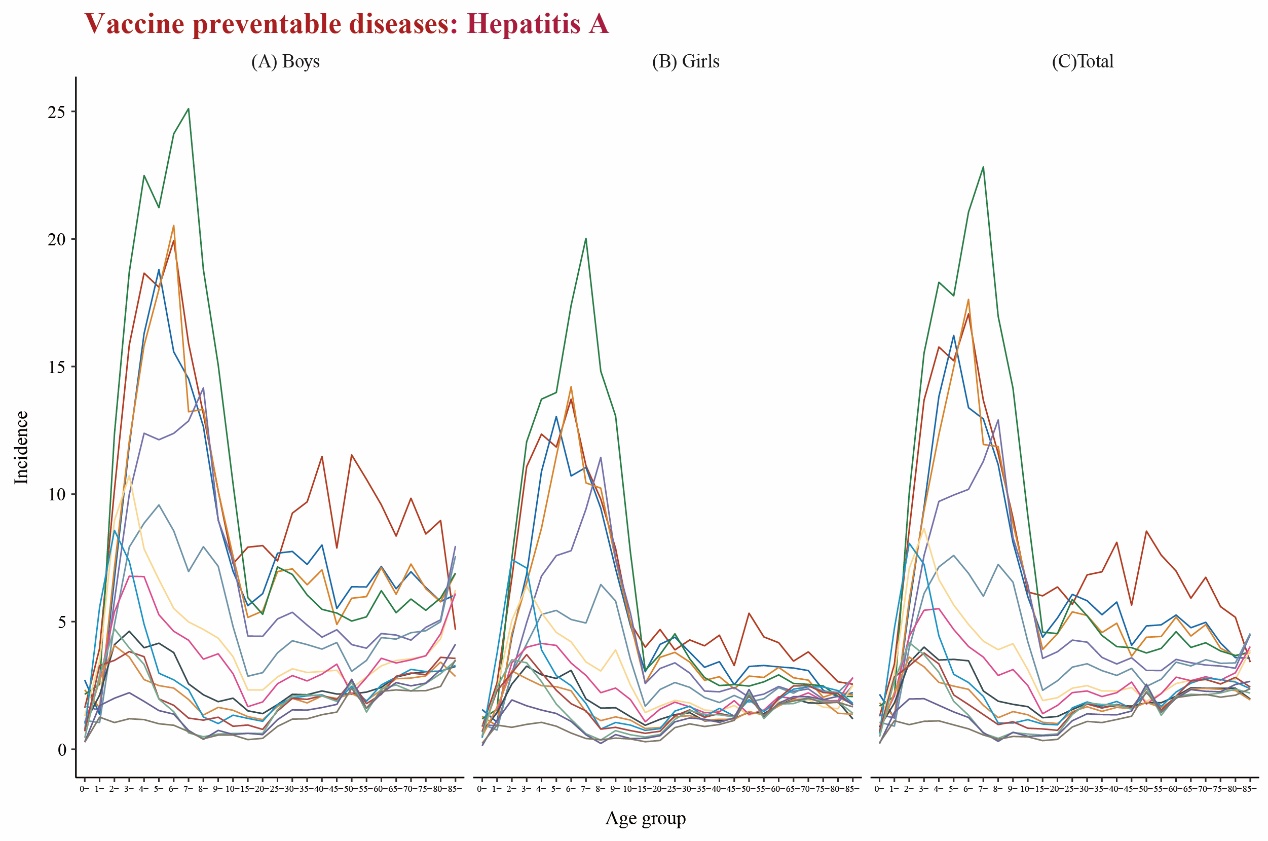


**Fig S5-9. Trends in age incidence rates for Hepatitis A, females and males, 2004-2018 (Legends were the same to Fig S5-1)**


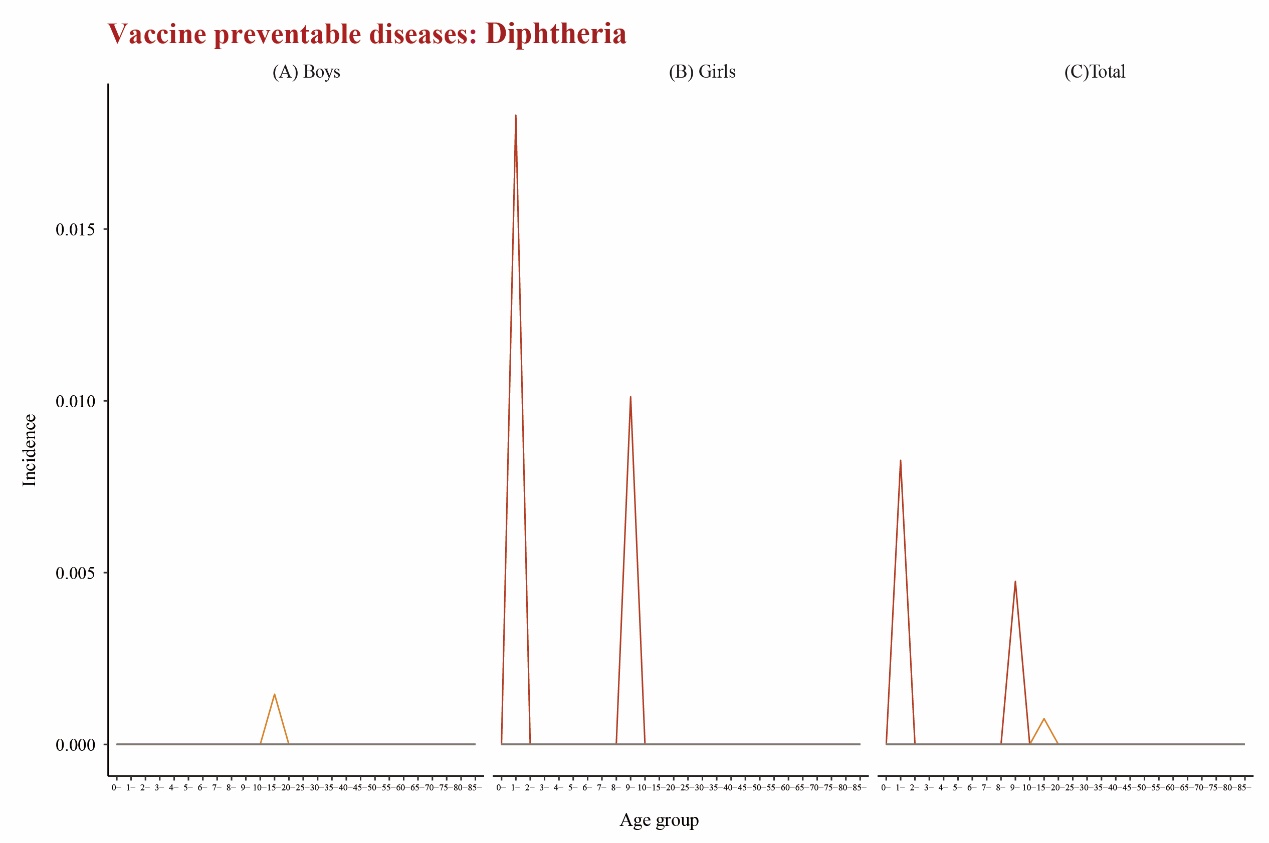


**Fig S5-10. Trends in age incidence rates for Diphtheria, females and males, 2004-2018 (Legends were the same to Fig S5-1)**


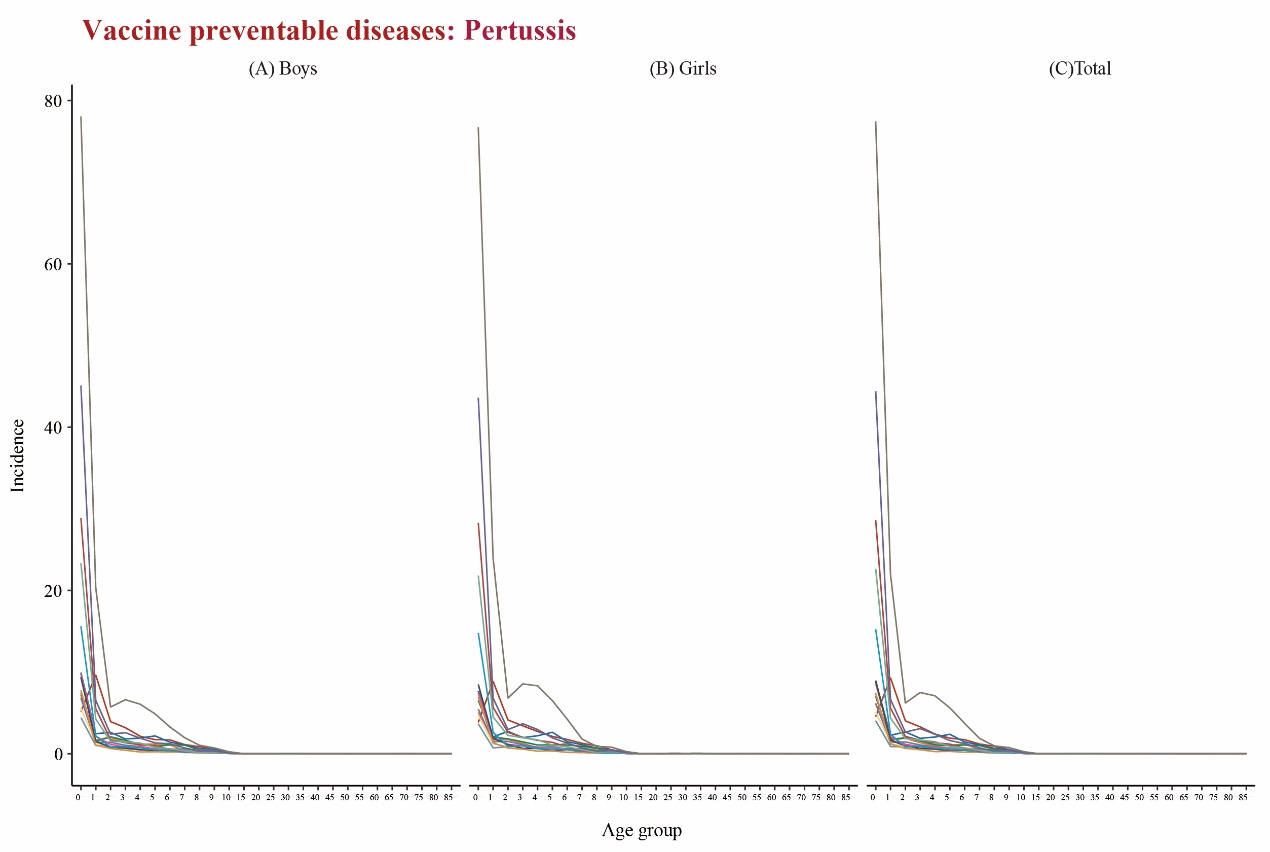


**Fig S5-11. Trends in age incidence rates for Pertussis, females and males, 2004-2018 (Legends were the same to Fig S5-1)**


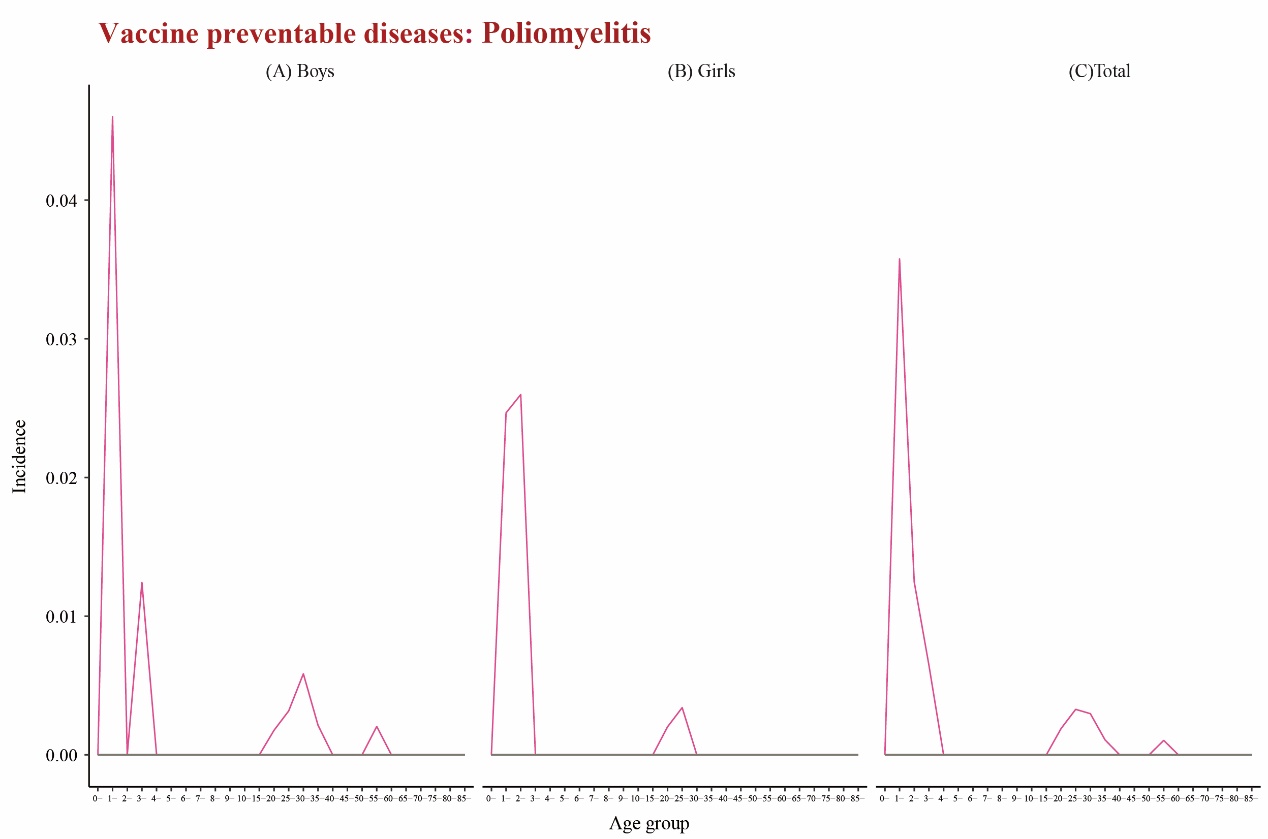


**Fig S5-12. Trends in age incidence rates for Poliomyelitis, females and males, 2004-2018 (Legends were the same to Fig S5-1)**


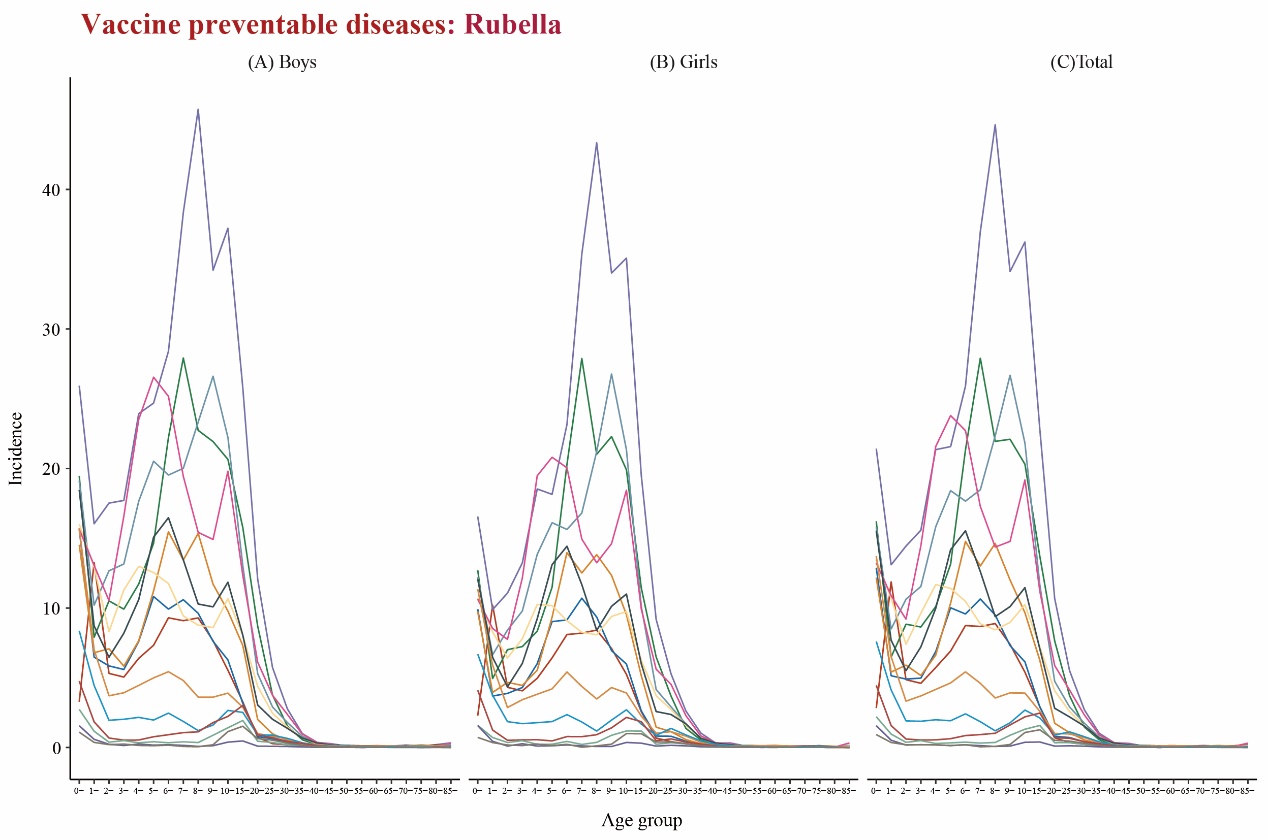


**Fig S5-13. Trends in age incidence rates for Rubella, females and males, 2004-2018 (Legends were the same to Fig S5-1)**


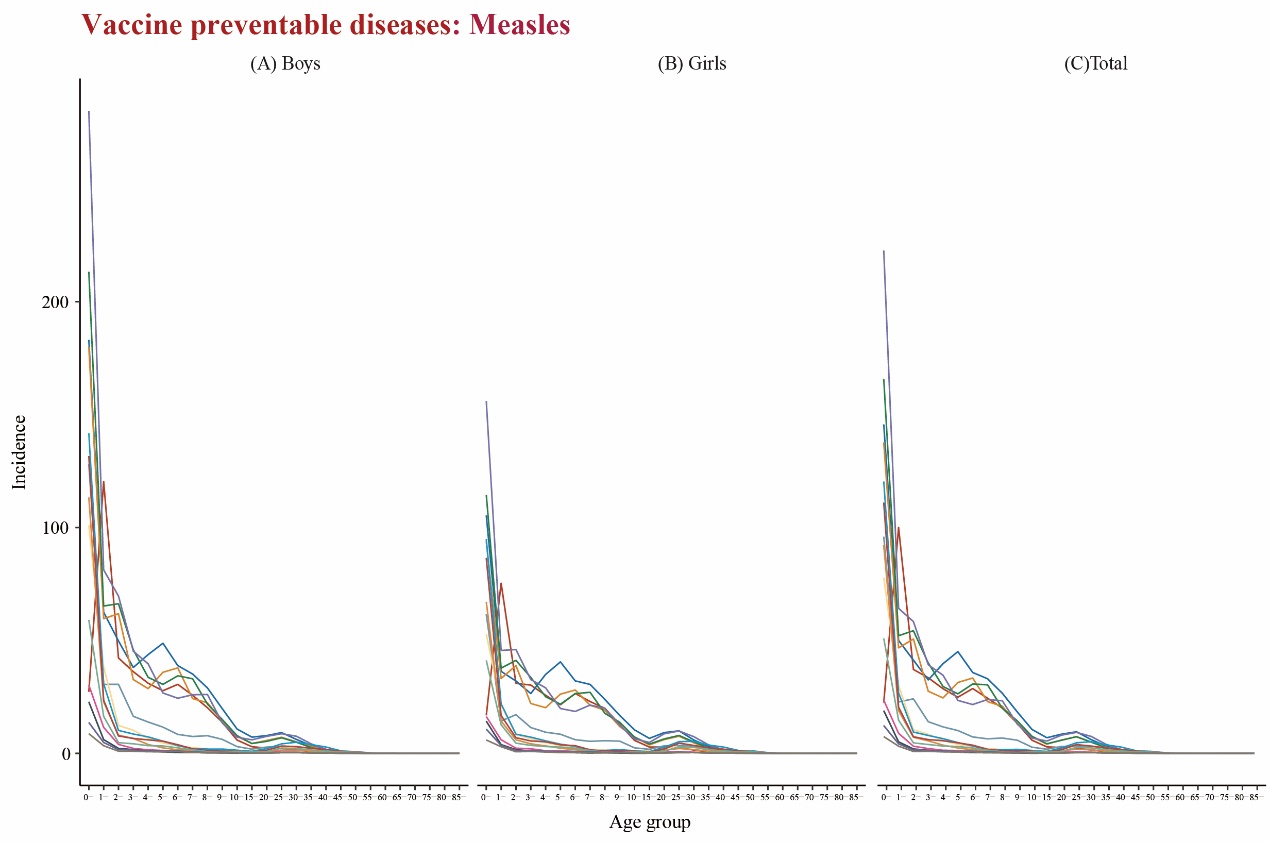


**Fig S5-14. Trends in age incidence rates for Measles, females and males, 2004-2018 (Legends were the same to Fig S5-1)**


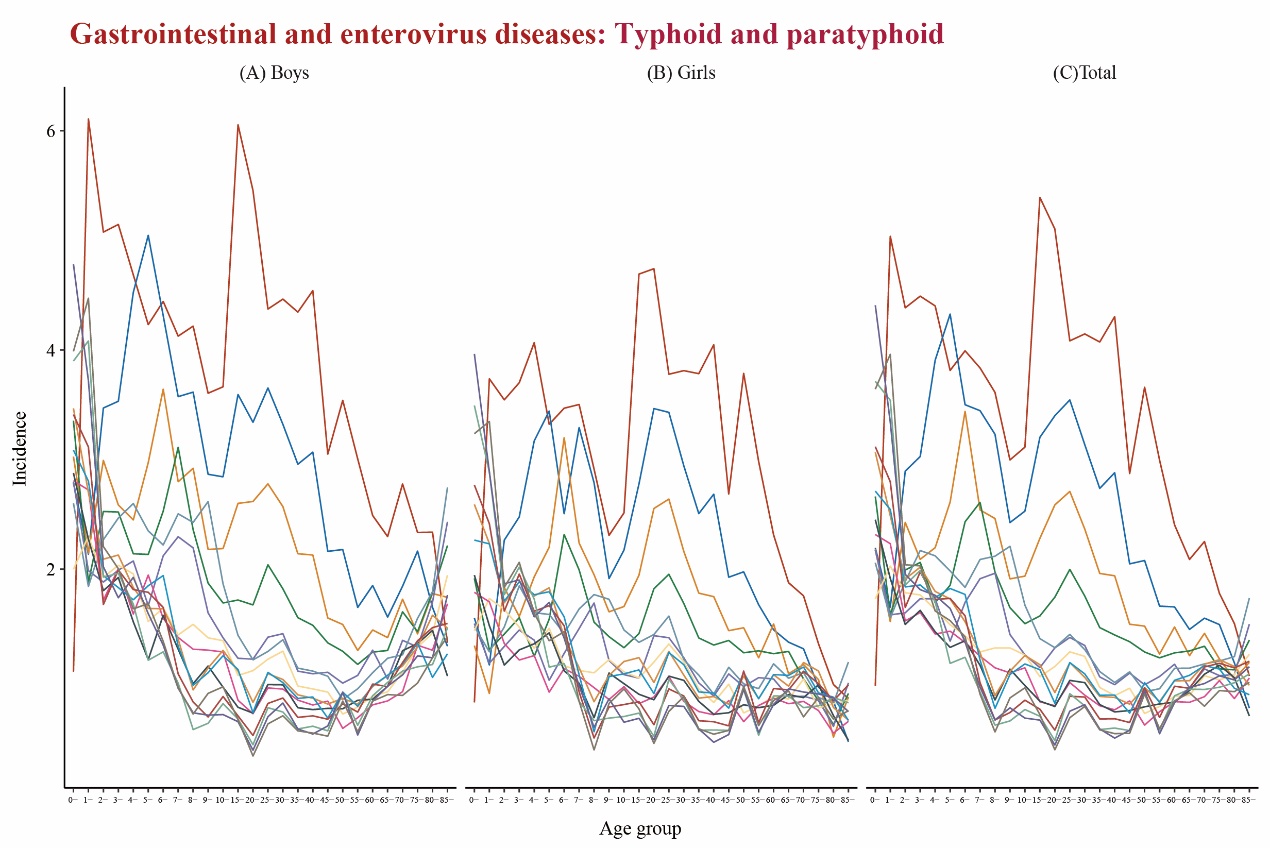


**Fig S5-15. Trends in age incidence rates for Typhoid and paratyphoid, females and males, 2004-2018 (Legends were the same to Fig S5-1)**


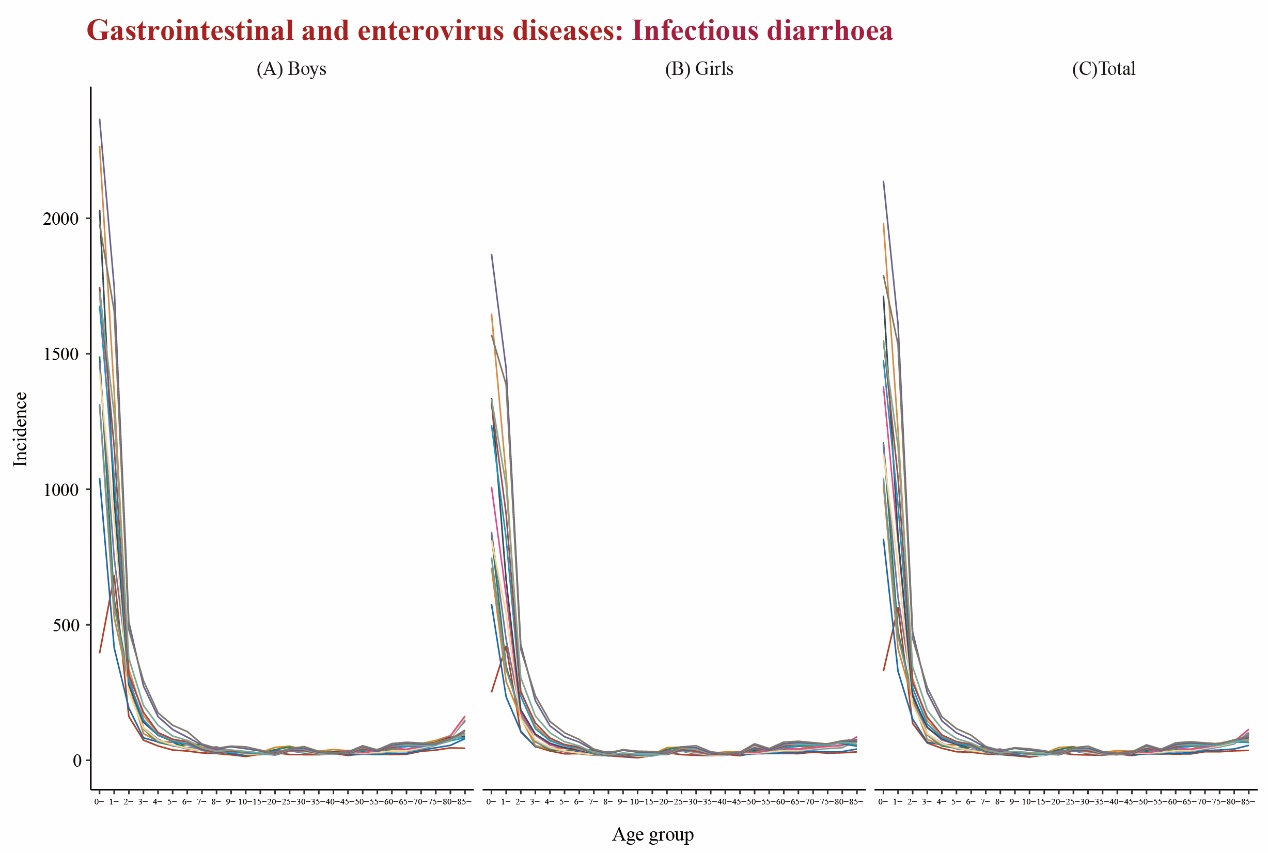


**Fig S5-16. Trends in age incidence rates for Infectious diarrhoea, females and males, 2004-2018 (Legends were the same to Fig S5-1)**


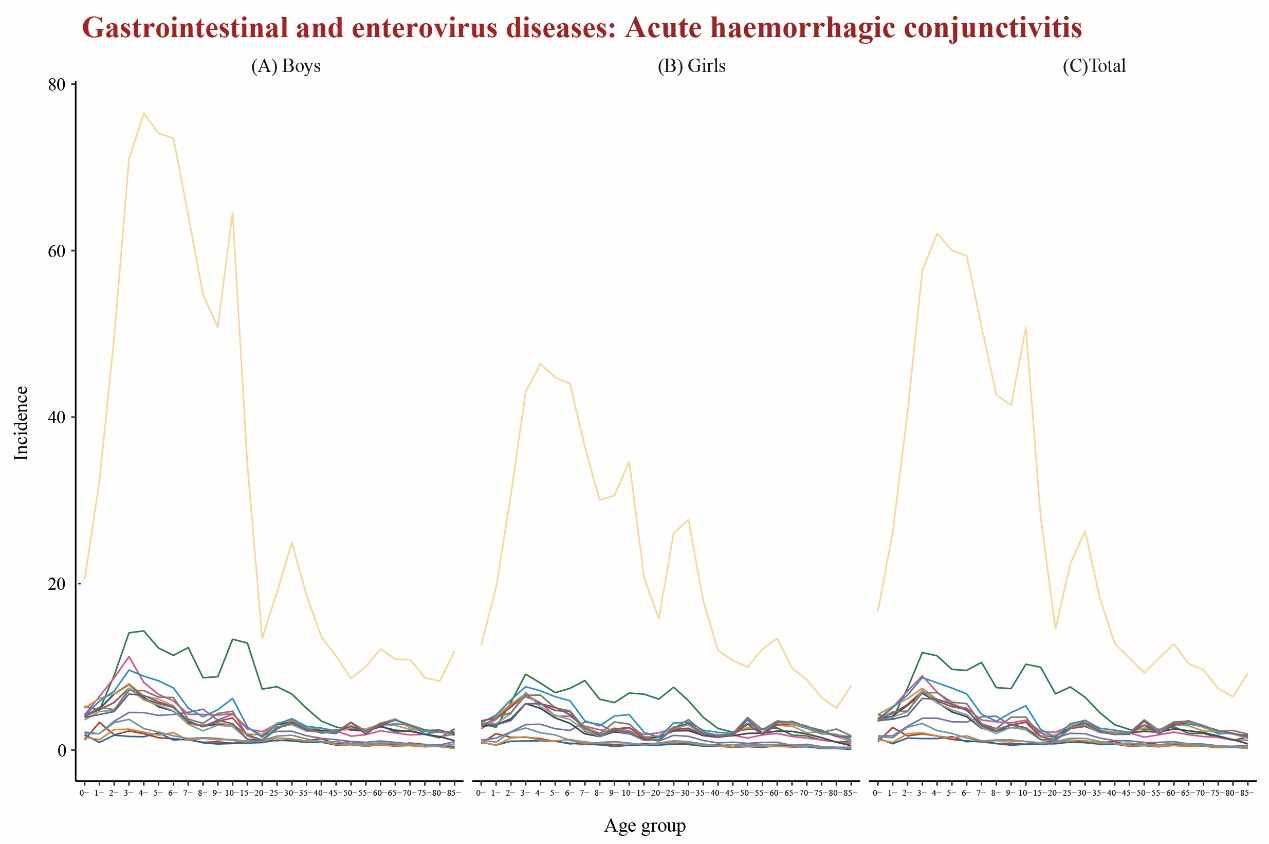


**Fig S5-17. Trends in age incidence rates for Acute haemorrhagic conjunctivitis, females and males, 2004-2018 (Legends were the same to Fig S5-1)**


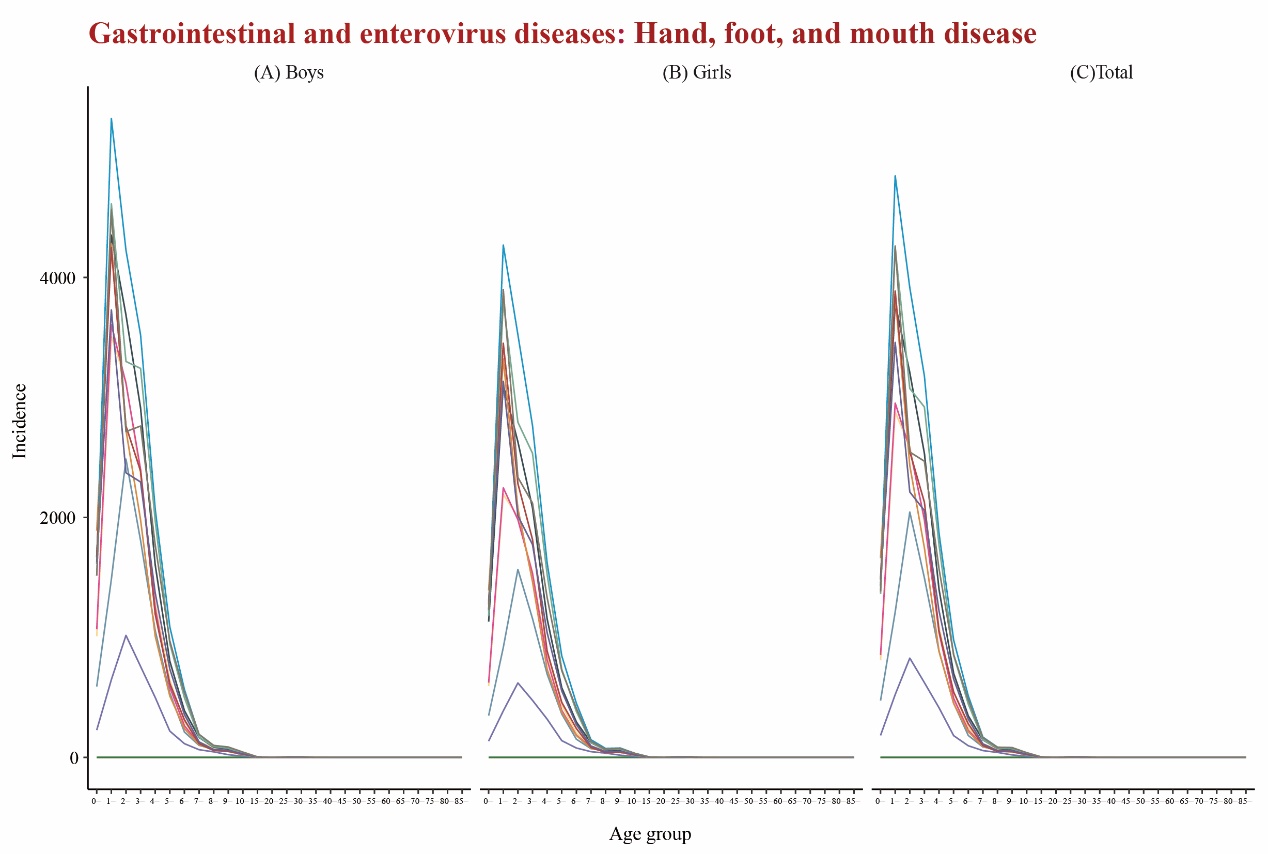


**Fig S5-18. Trends in age incidence rates for Hand, foot, and mouth disease, females and males, 2004-2018 (Legends were the same to Fig S5-1)**


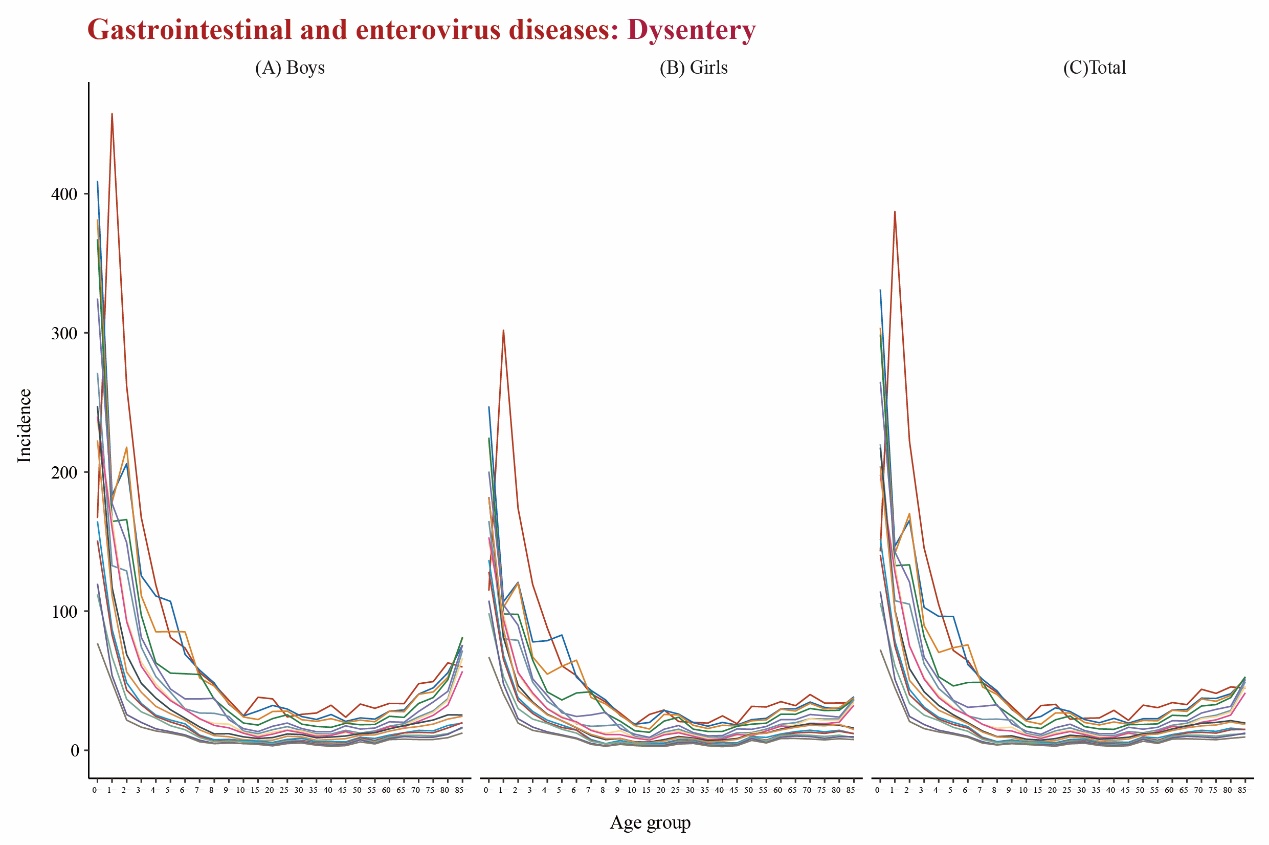


**Fig S5-19. Trends in age incidence rates for Dysentery, females and males, 2004-2018 (Legends were the same to Fig S5-1)**


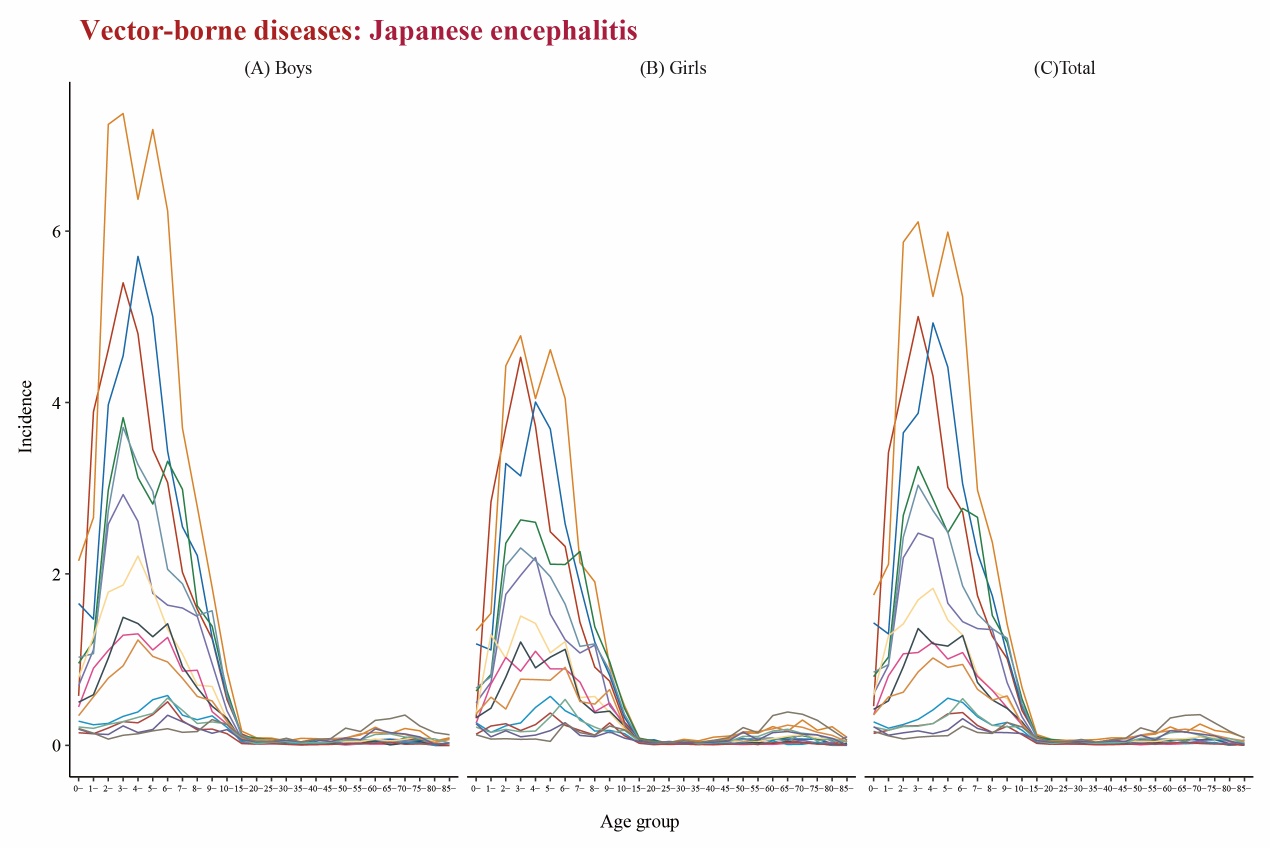


**Fig S5-20. Trends in age incidence rates for Japanese encephalitis, females and males, 2004-2018 (Legends were the same to Fig S5-1)**


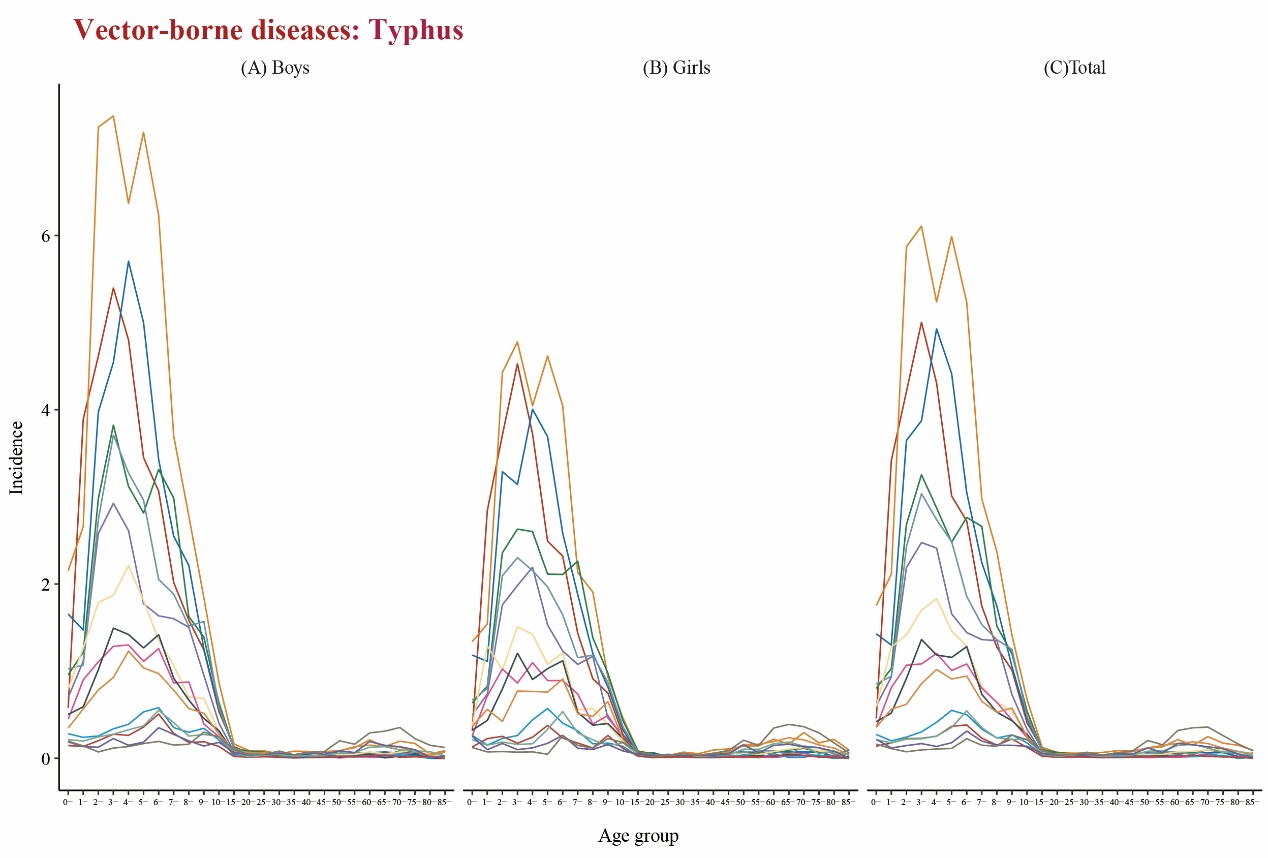


**Fig S5-21. Trends in age incidence rates for Typhus, females and males, 2004-2018 (Legends were the same to Fig S5-1)**


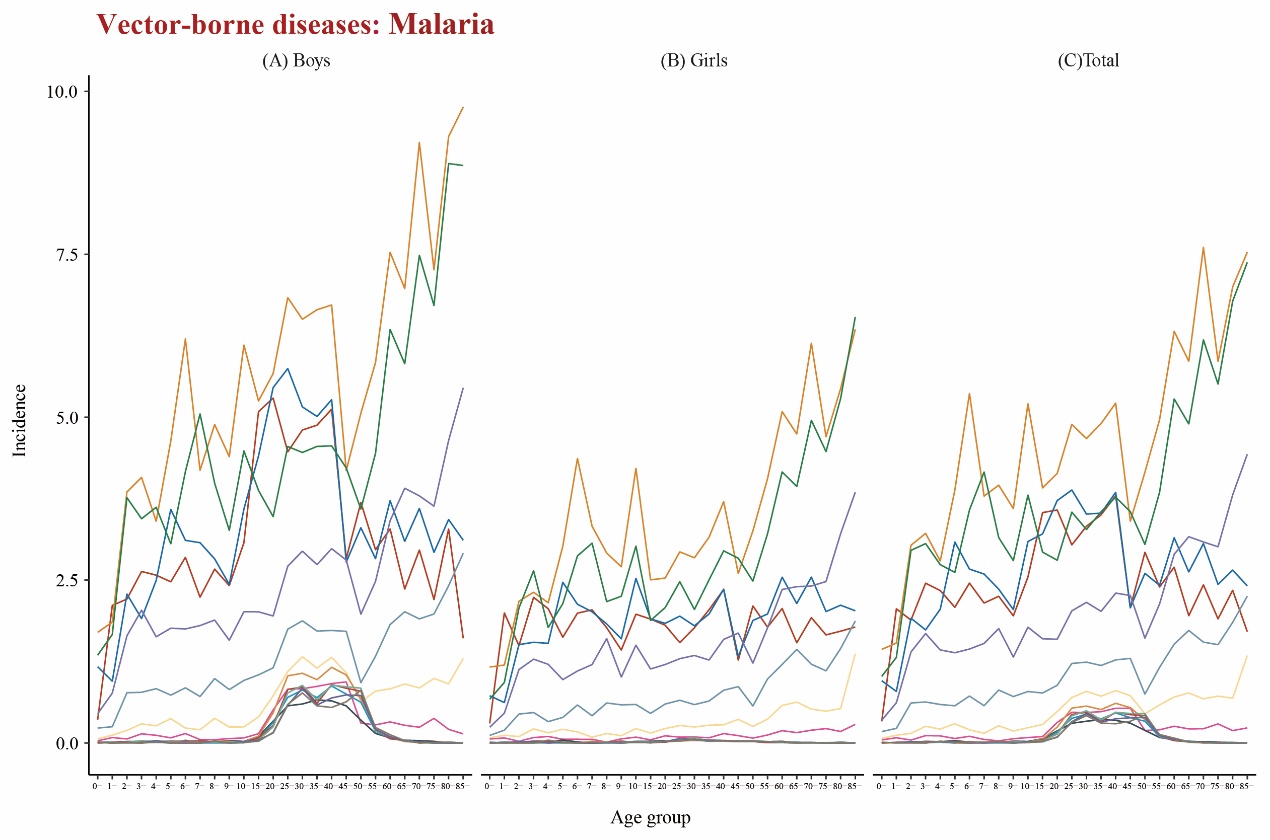


**Fig S5-22. Trends in age incidence rates for Malaria, females and males, 2004-2018 (Legends were the same to Fig S5-1)**


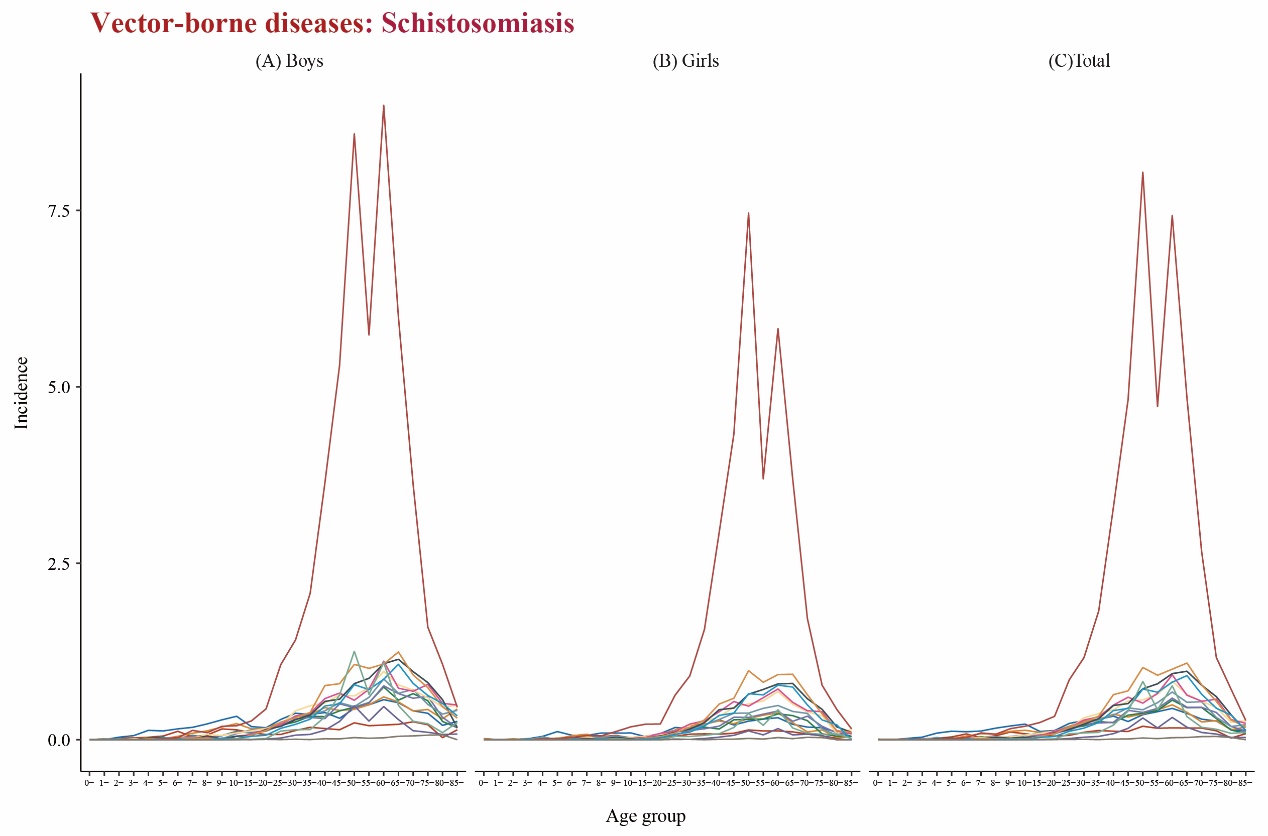


**Fig S5-23. Trends in age incidence rates for Schistosomiasis, females and males, 2004-2018 (Legends were the same to Fig S5-1)**


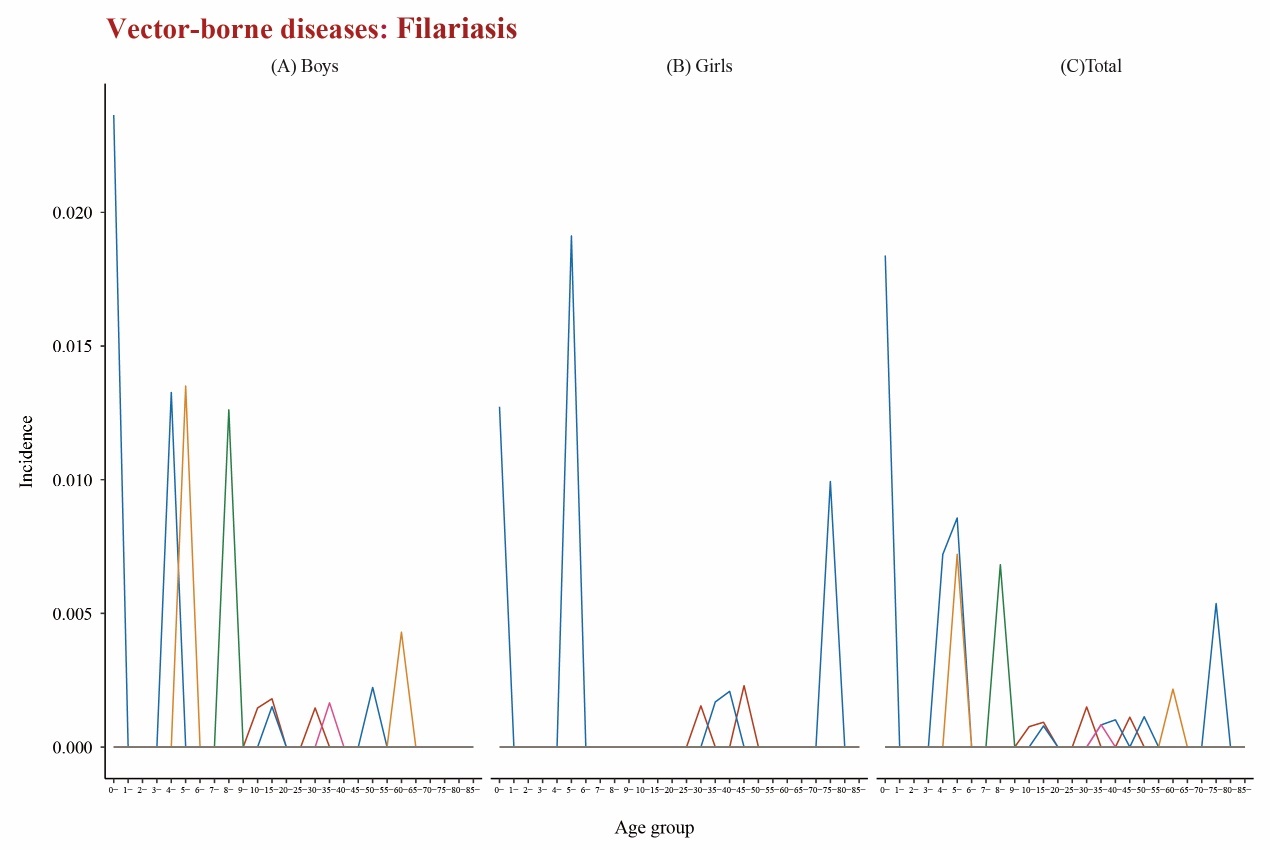


**Fig S5-24. Trends in age incidence rates for Filariasis, females and males, 2004-2018 (Legends were the same to Fig S5-1)**


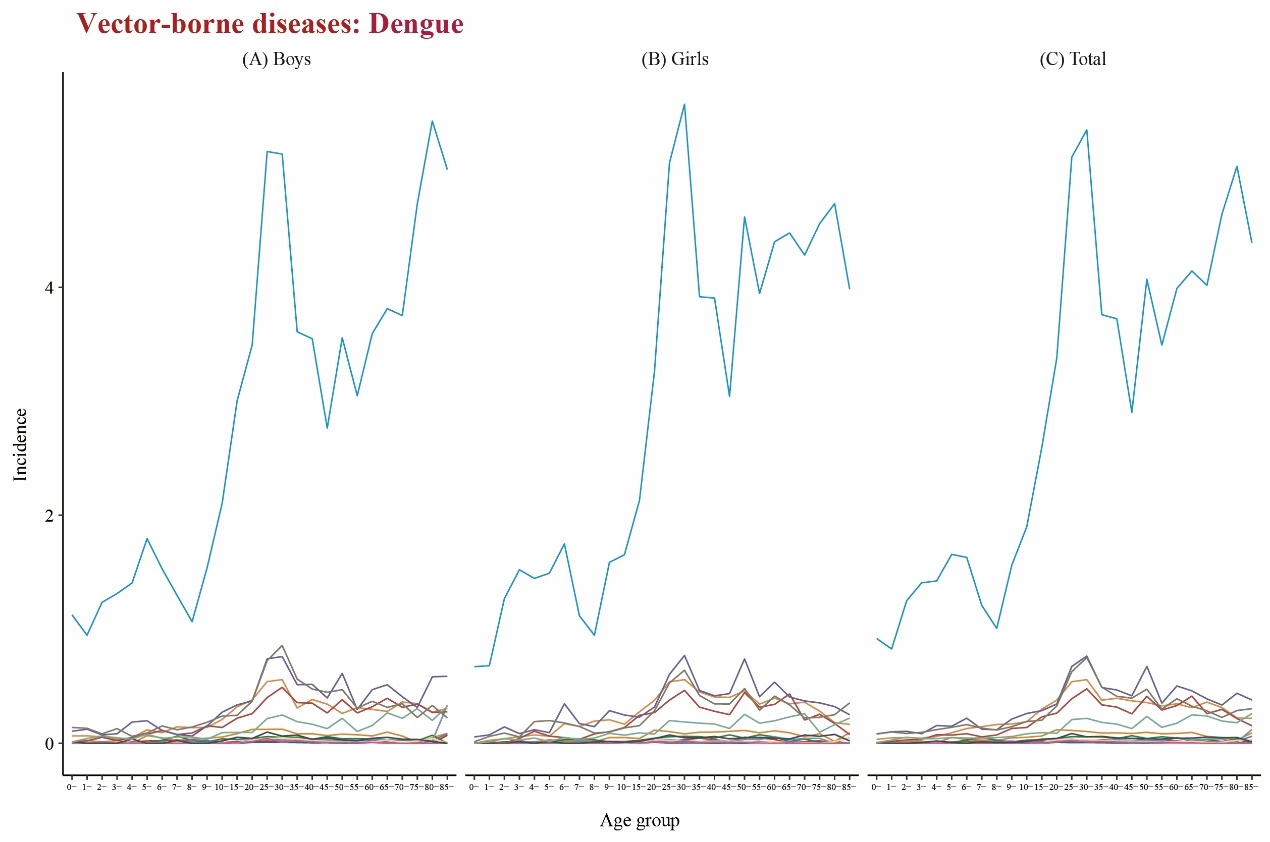


**Fig S5-25. Trends in age incidence rates for Dengue, females and males, 2004-2018 (Legends were the same to Fig S5-1)**


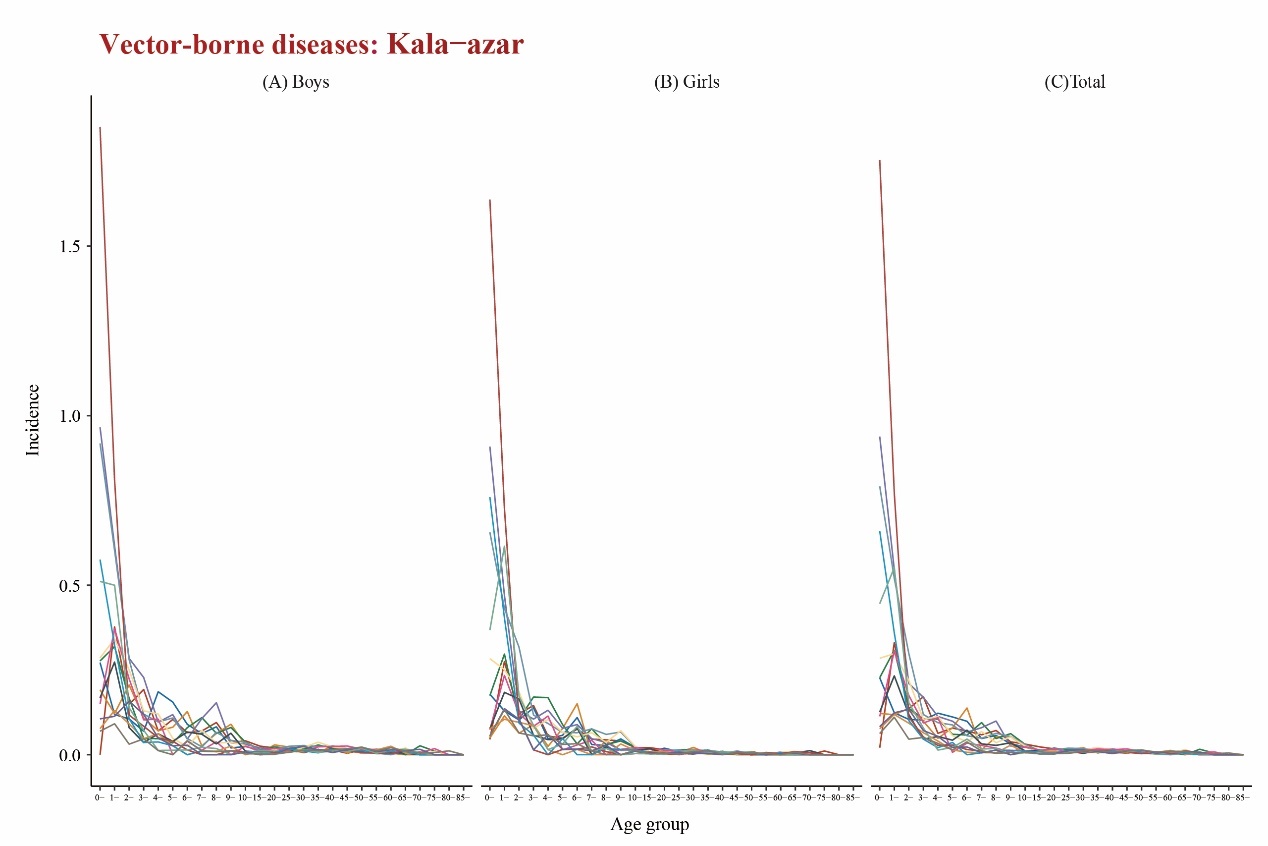


**Fig S5-26. Trends in age incidence rates for Kala−azar, females and males, 2004-2018 (Legends were the same to Fig S5-1)**


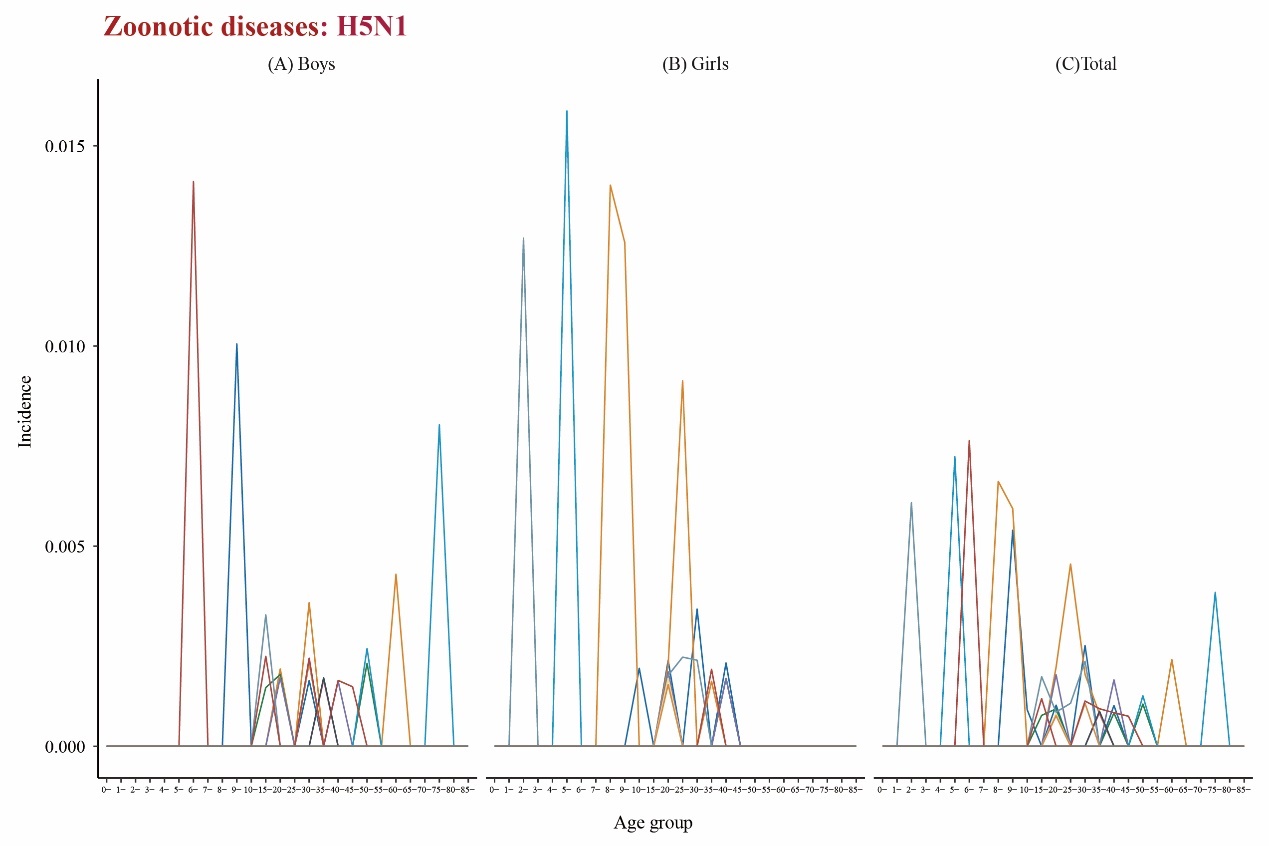


**Fig S5-27. Trends in age incidence rates for H5N1, females and males, 2004-2018 (Legends were the same to Fig S5-1)**


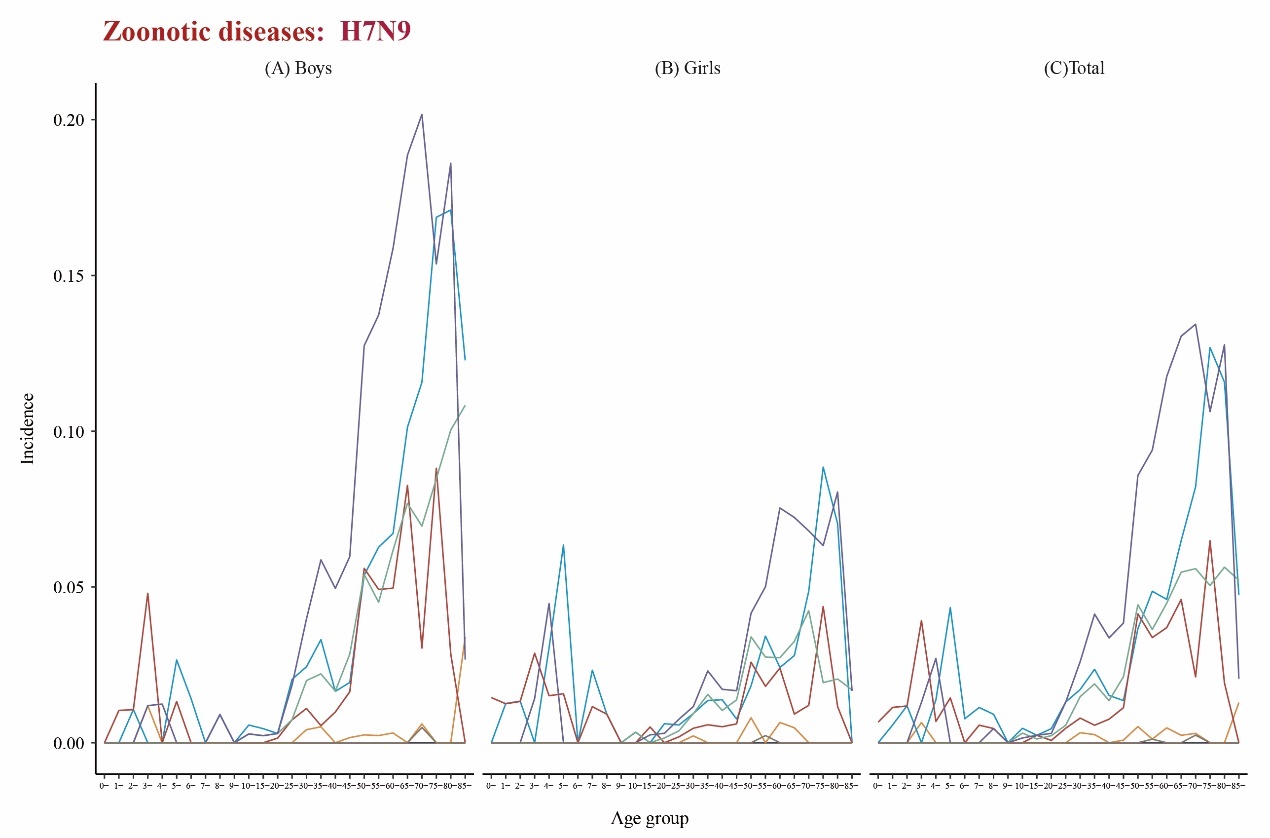


**Fig S5-28. Trends in age incidence rates for H7N9, females and males, 2004-2018 (Legends were the same to Fig S5-1)**


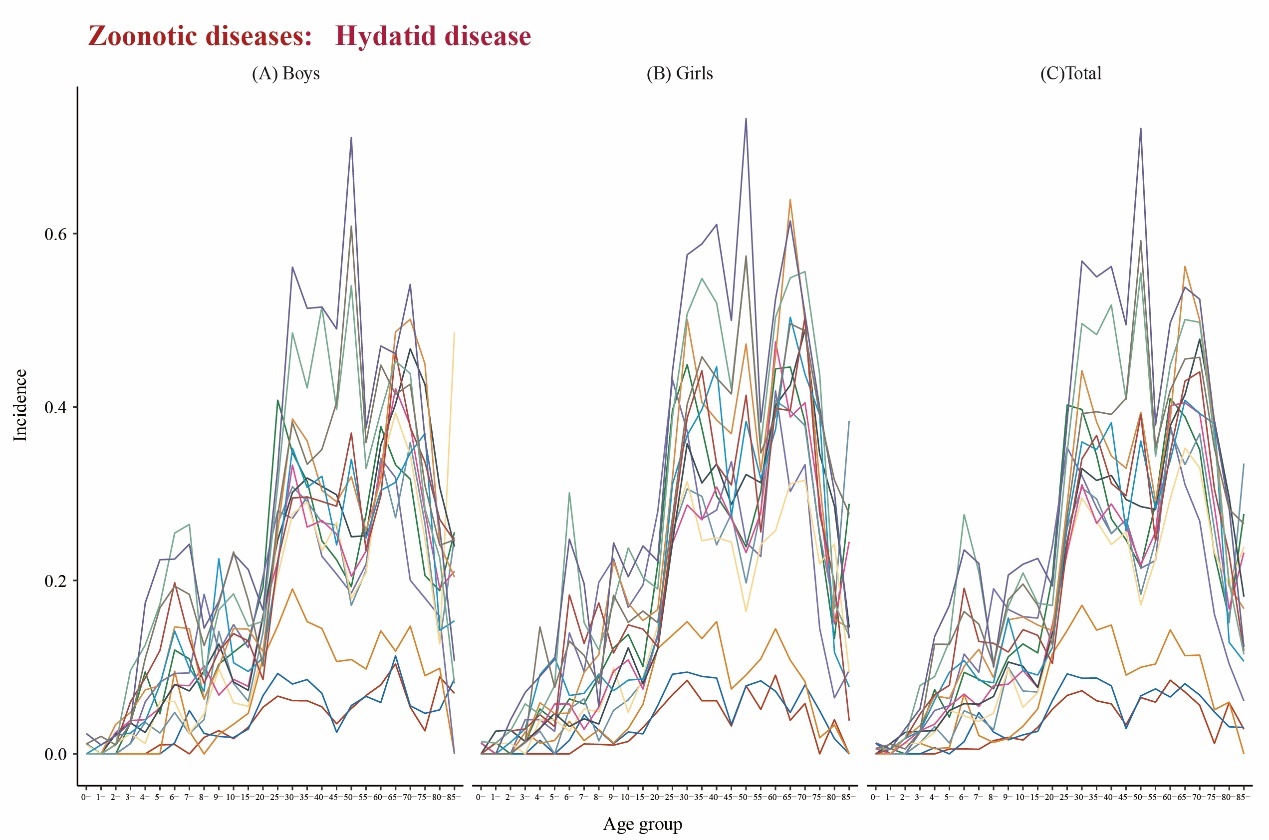


**Fig S5-29. Trends in age incidence rates for Hydatid disease, females and males, 2004-2018 (Legends were the same to Fig S5-1)**


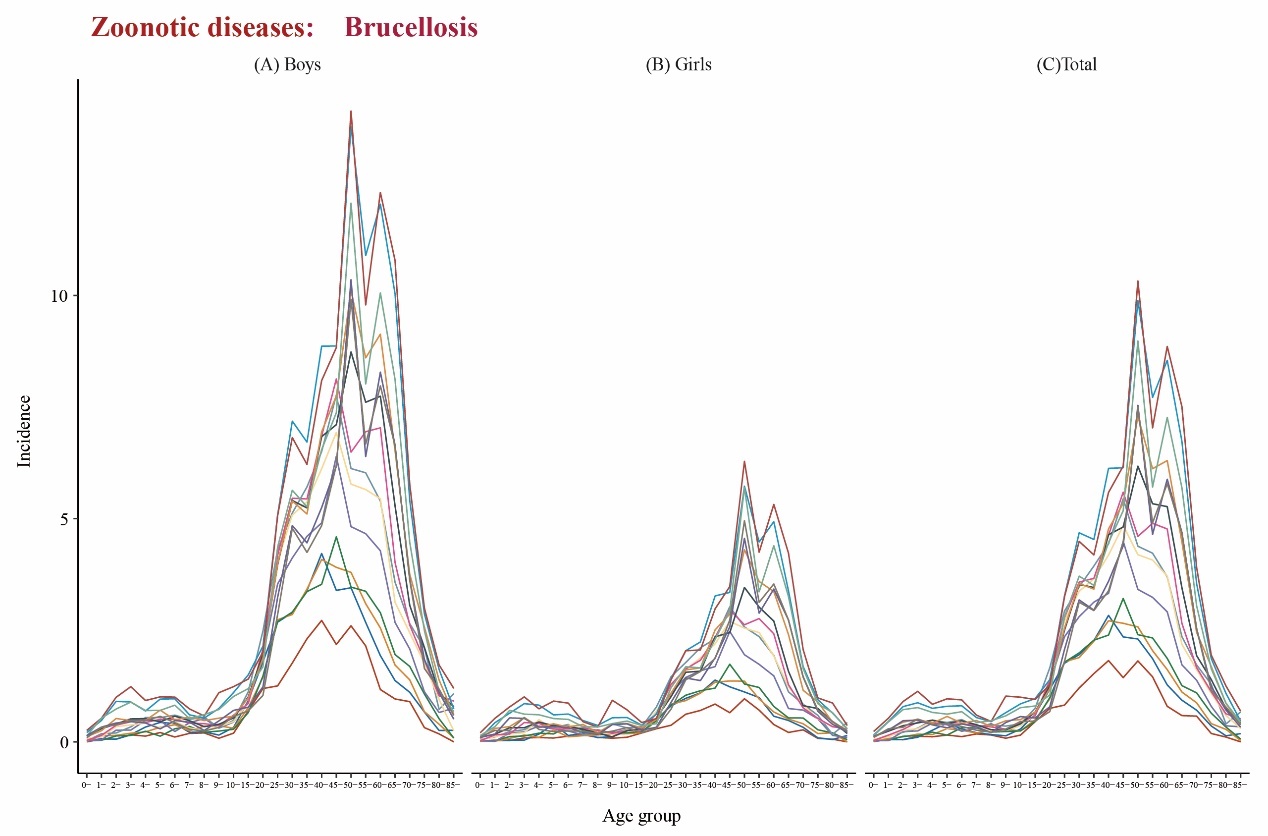


**Fig S5-30. Trends in age incidence rates for Brucellosis, females and males, 2004-2018 (Legends were the same to Fig S5-1)**


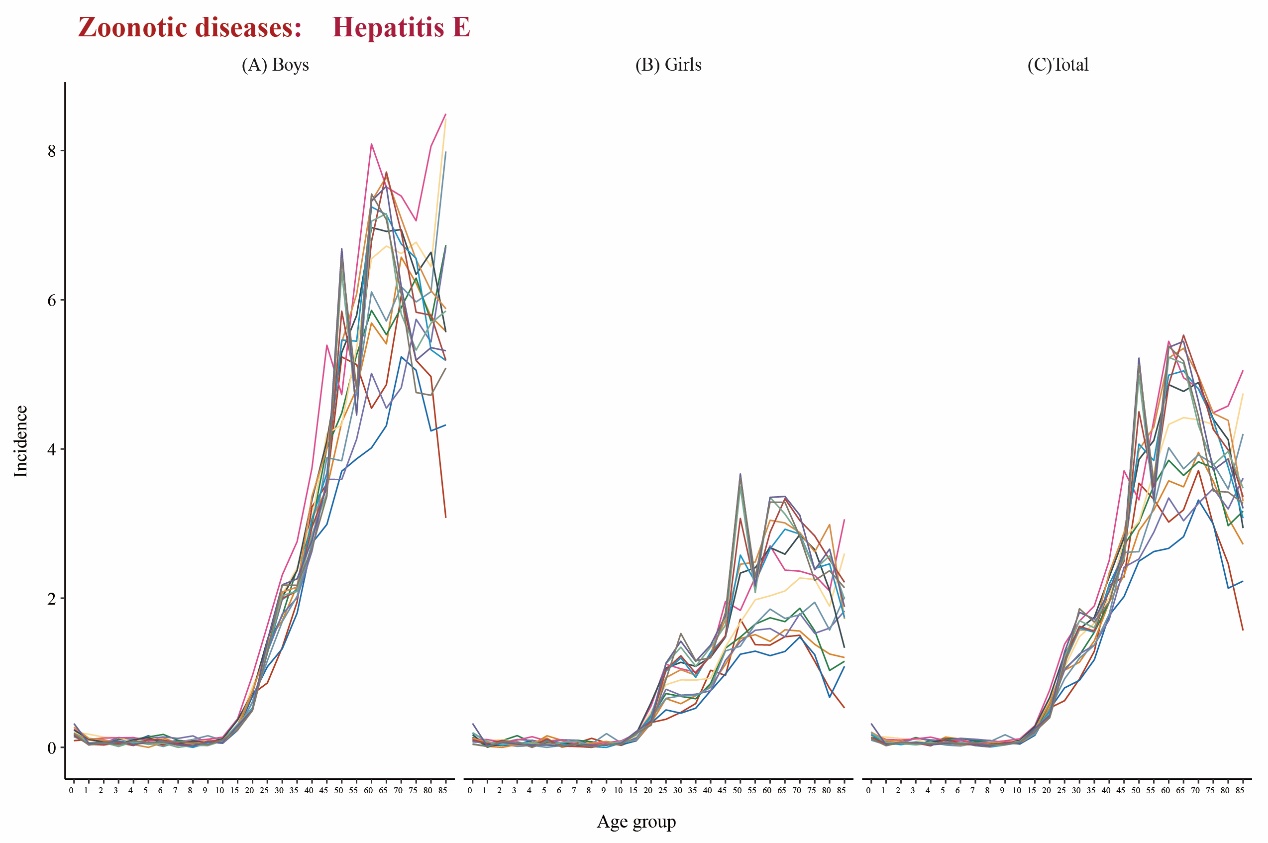


**Fig S5-31. Trends in age incidence rates for Hepatitis E, females and males, 2004-2018 (Legends were the same to Fig S5-1)**


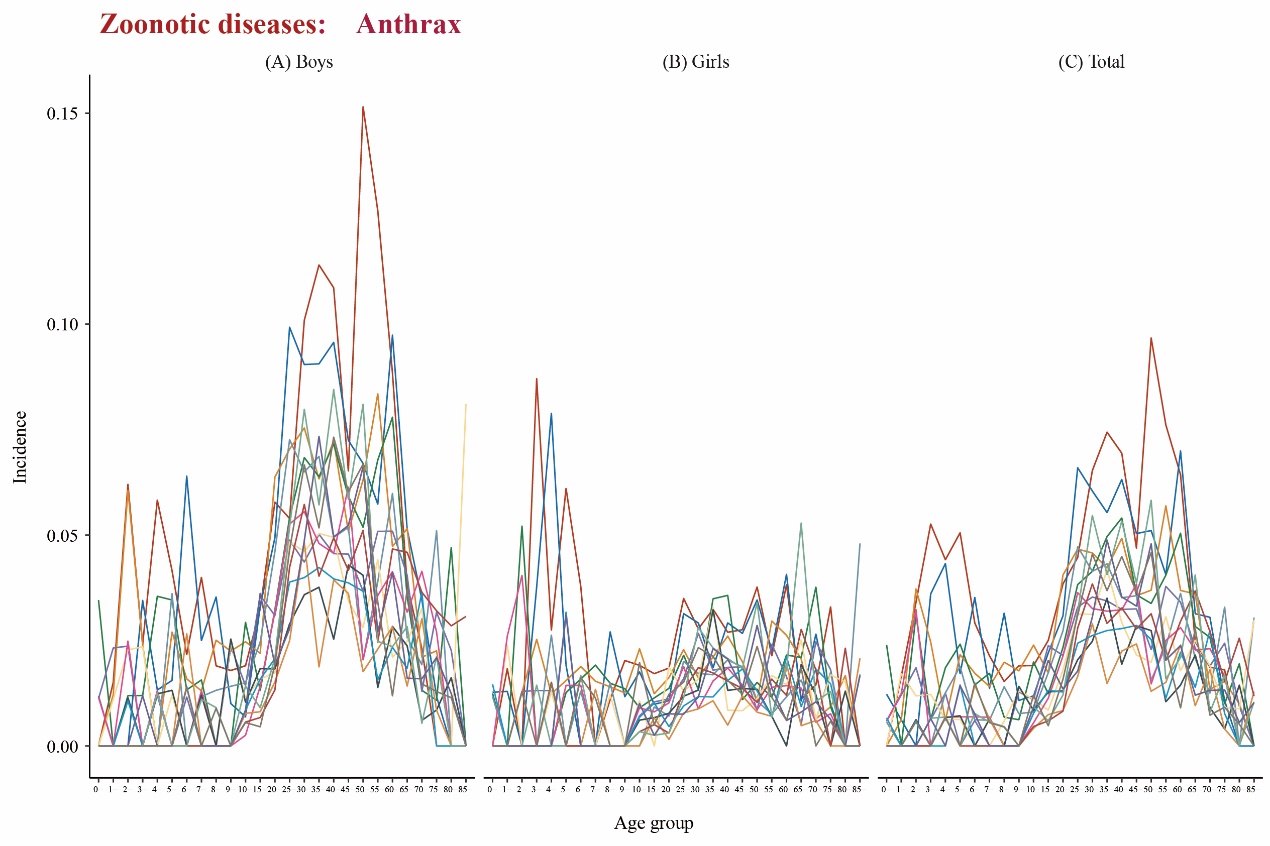


**Fig S5-32. Trends in age incidence rates for Anthrax, females and males, 2004-2018 (Legends were the same to Fig S5-1)**


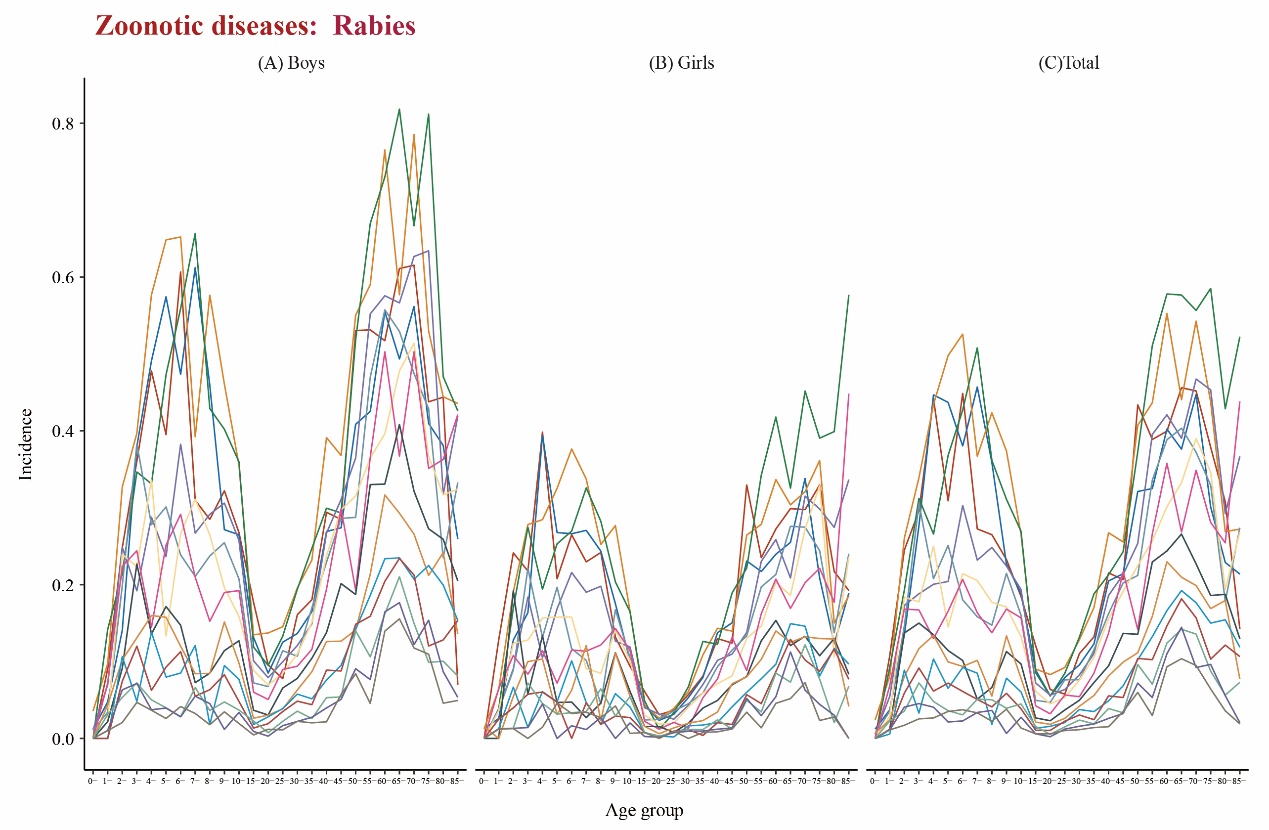


**Fig S5-33. Trends in age incidence rates for Rabies, females and males, 2004-2018 (Legends were the same to Fig S5-1)**


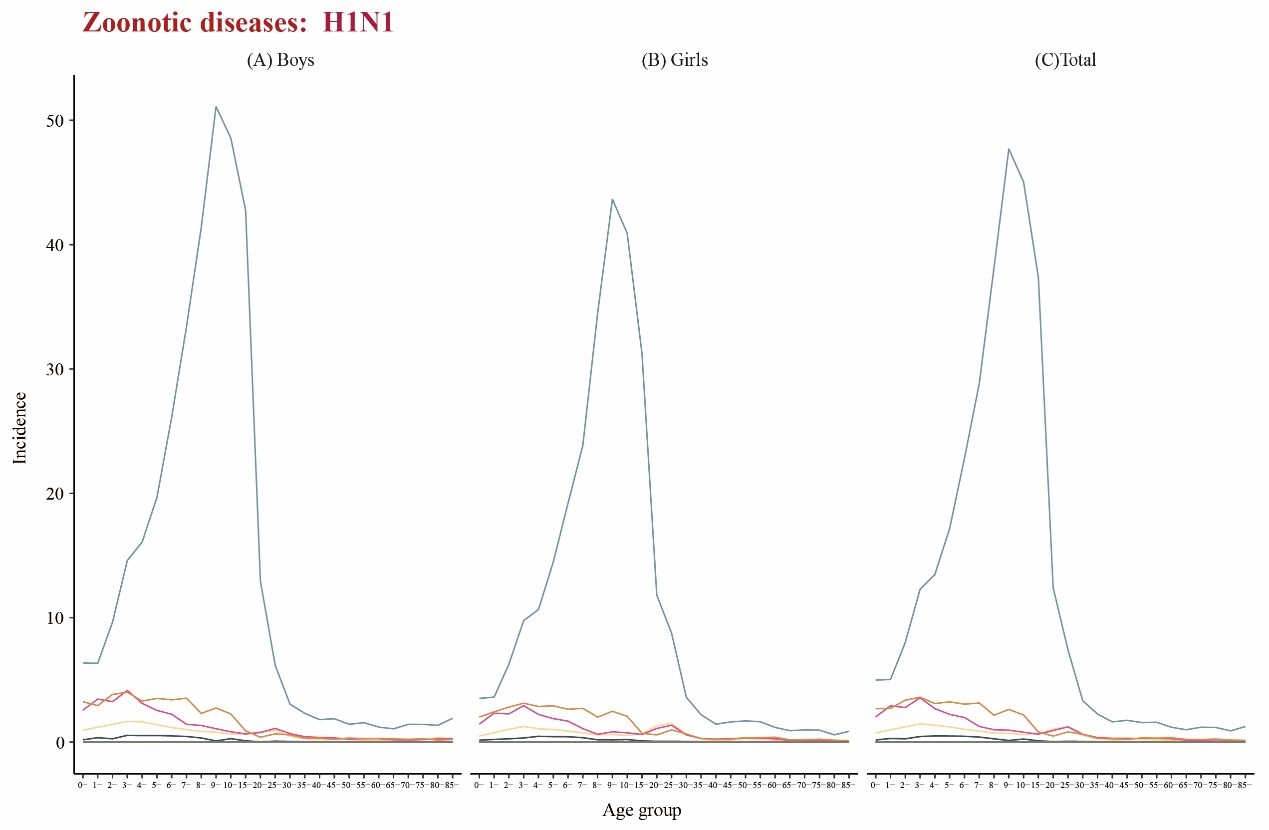


**Fig S5-34. Trends in age incidence rates for H1N1, females and males, 2004-2018 (Legends were the same to Fig S5-1)**


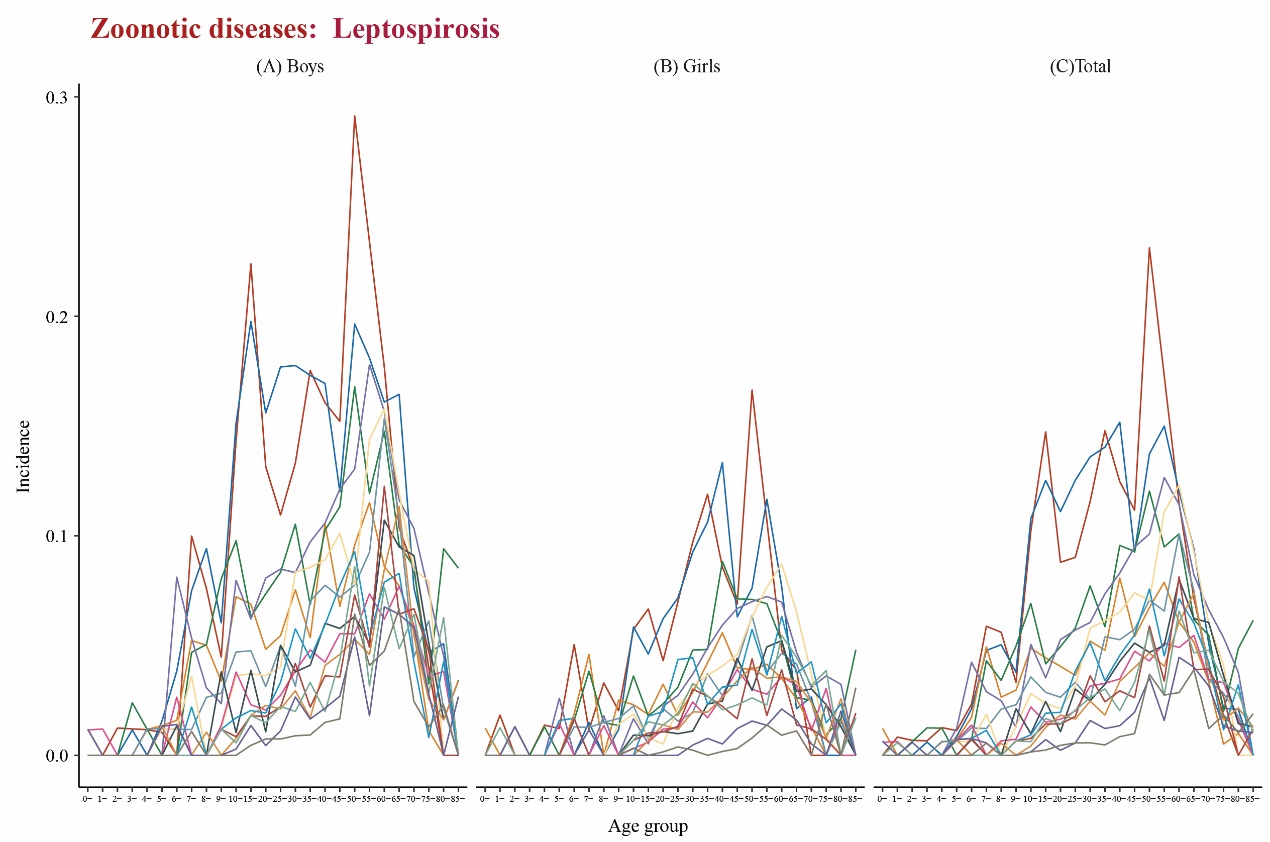


**Fig S5-35. Trends in age incidence rates for Leptospirosis, females and males, 2004-2018 (Legends were the same to Fig S5-1)**


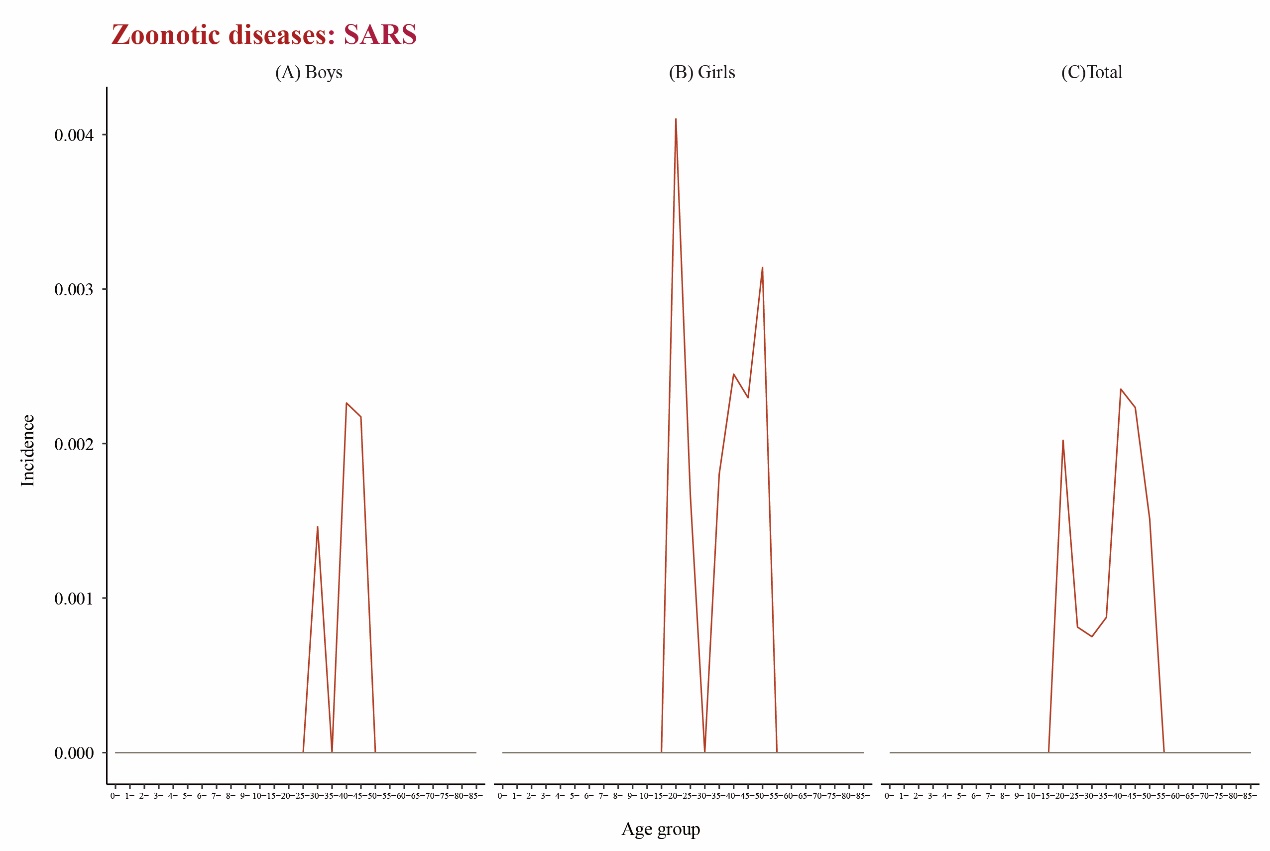


**Fig S5-36. Trends in age incidence rates for SARS, females and males, 2004-2018 (Legends were the same to Fig S5-1)**


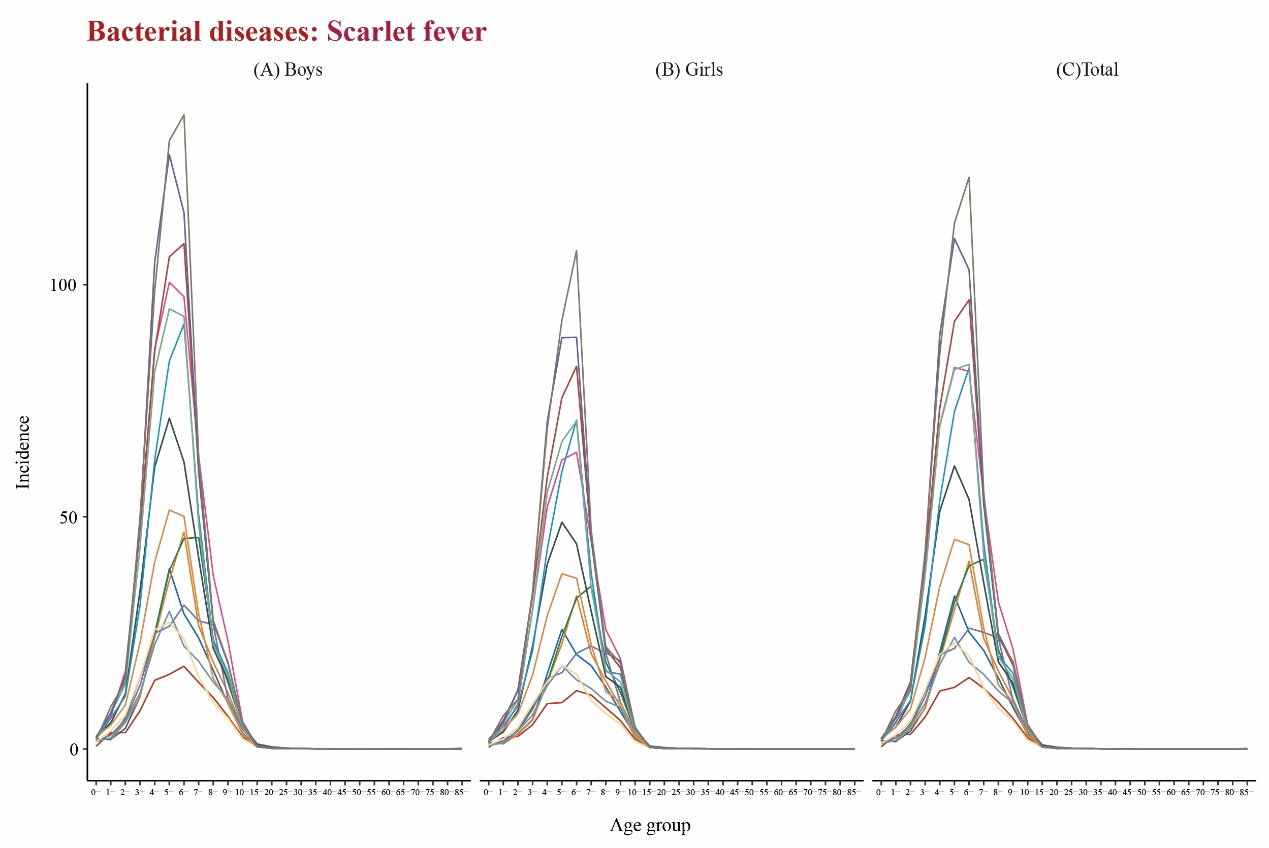


**Fig S5-37. Trends in age incidence rates for Scarlet fever, females and males, 2004-2018 (Legends were the same to Fig S5-1)**


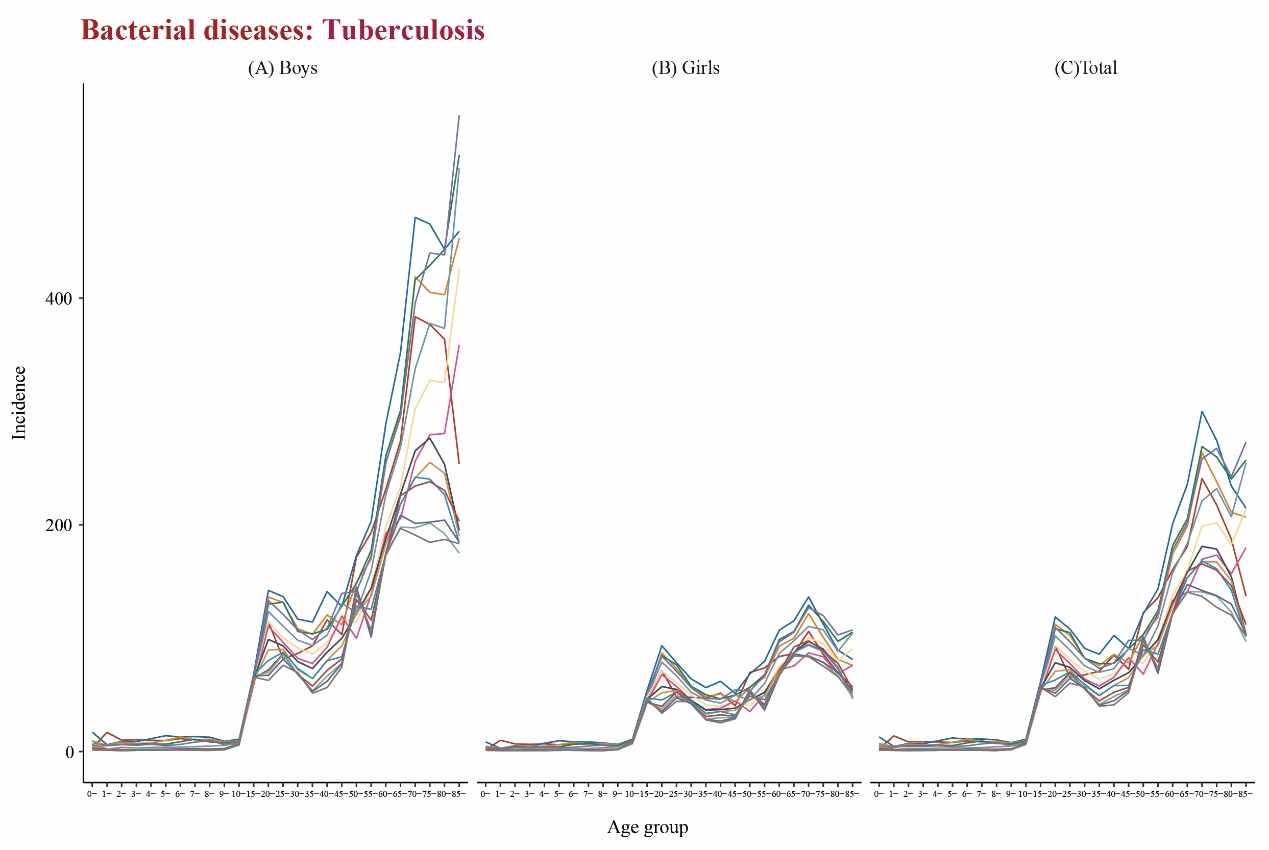


**Fig S5-38. Trends in age incidence rates for Tuberculosis, females and males, 2004-2018 (Legends were the same to Fig S5-1)**


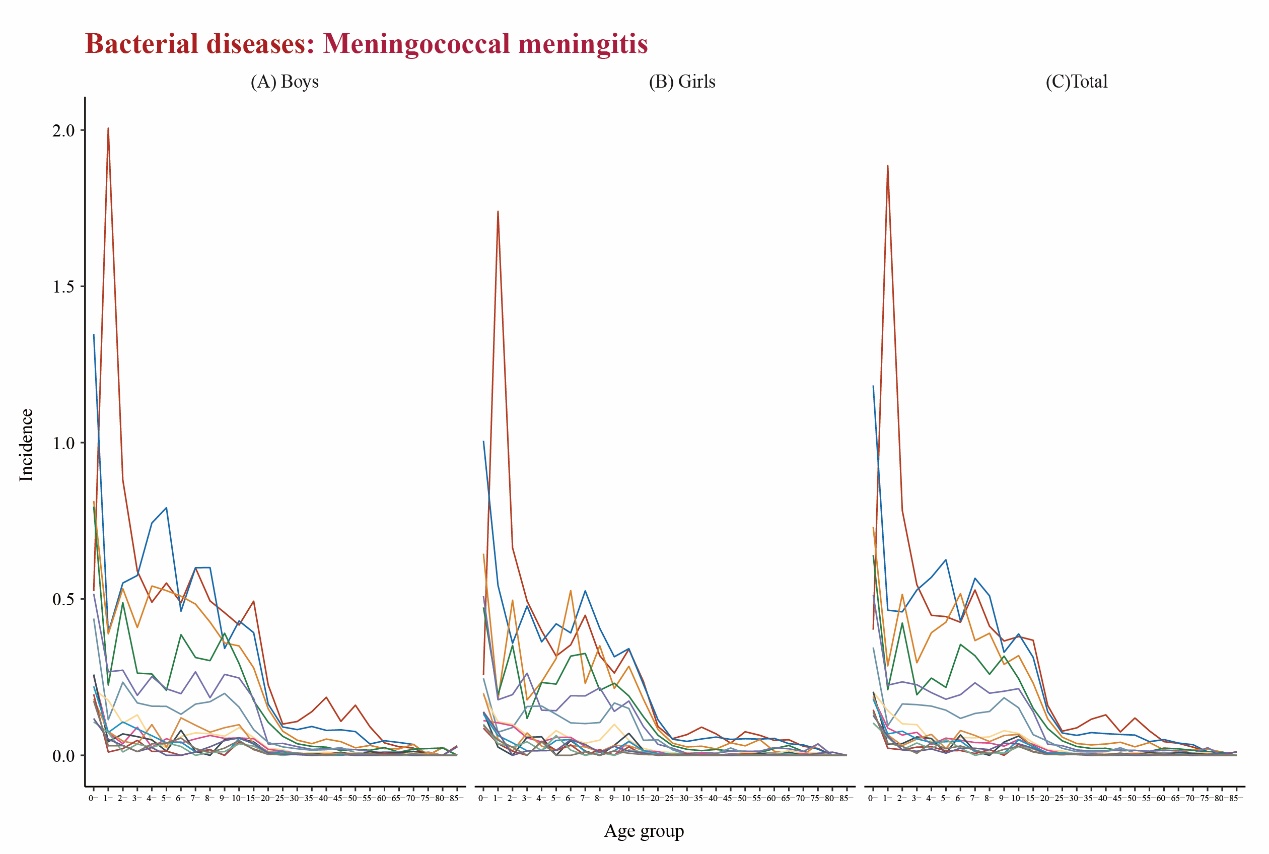


**Fig S5-39. Trends in age incidence rates for Meningococcal meningitis, females and males, 2004-2018 (Legends were the same to Fig S5-1)**
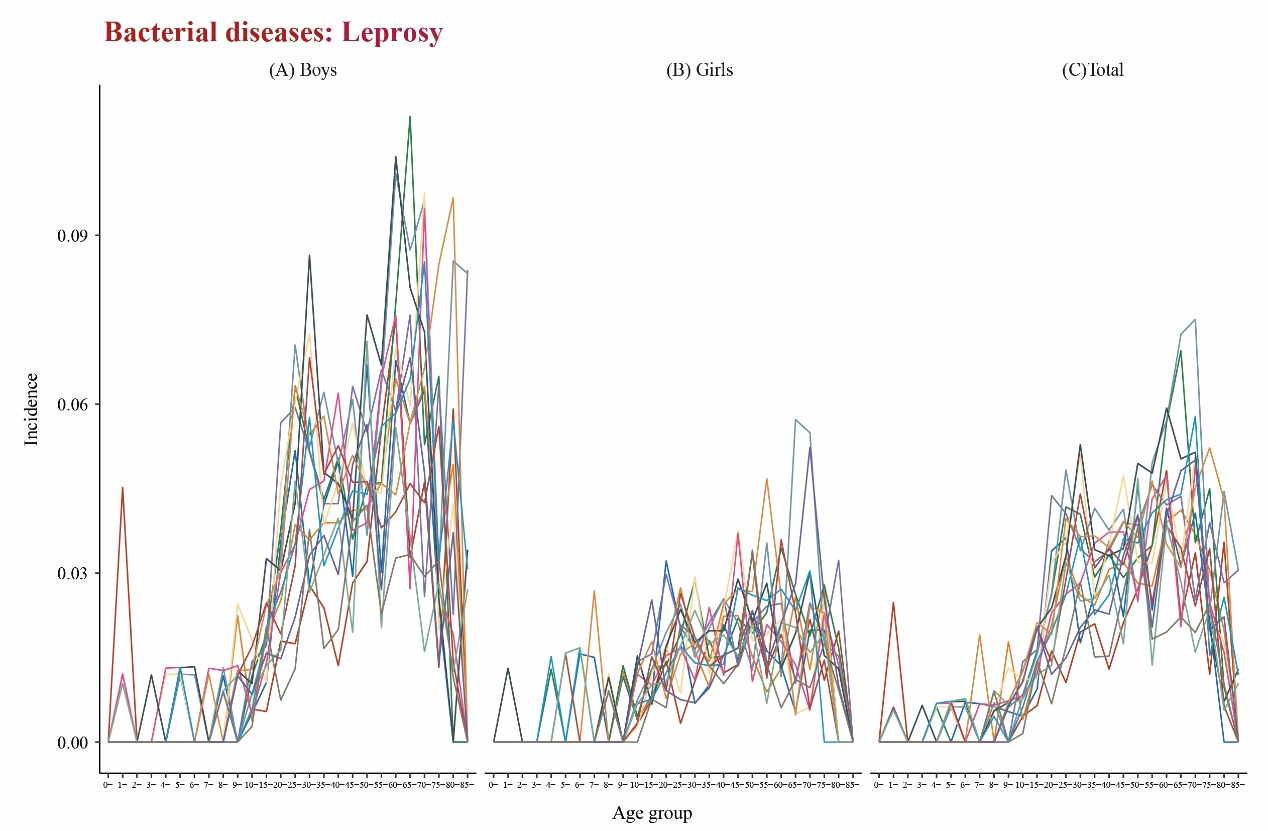


**Fig S5-40. Trends in age incidence rates for Leprosy, females and males, 2004-2018 (Legends were the same to Fig S5-1)**


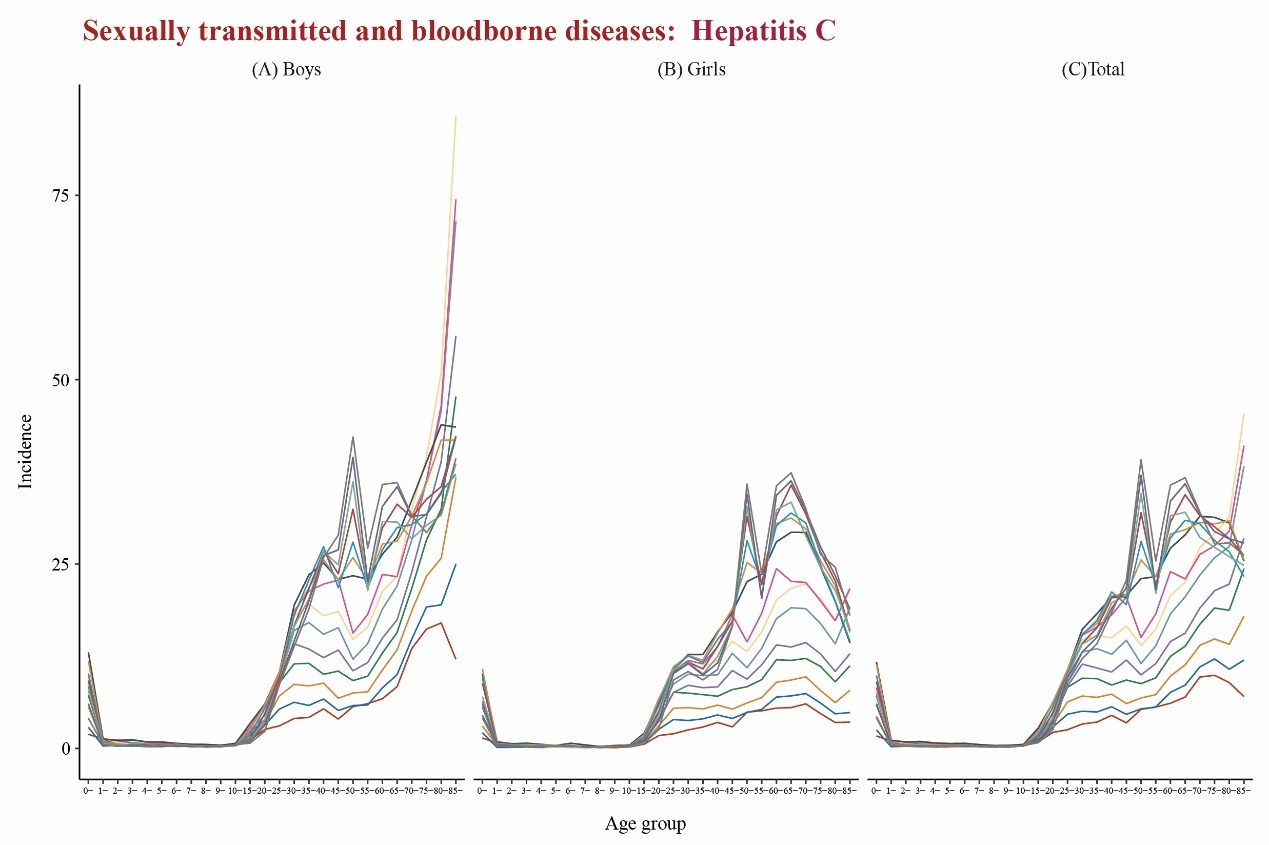


**Fig S5-41. Trends in age incidence rates for Hepatitis C, females and males, 2004-2018 (Legends were the same to Fig S5-1)**


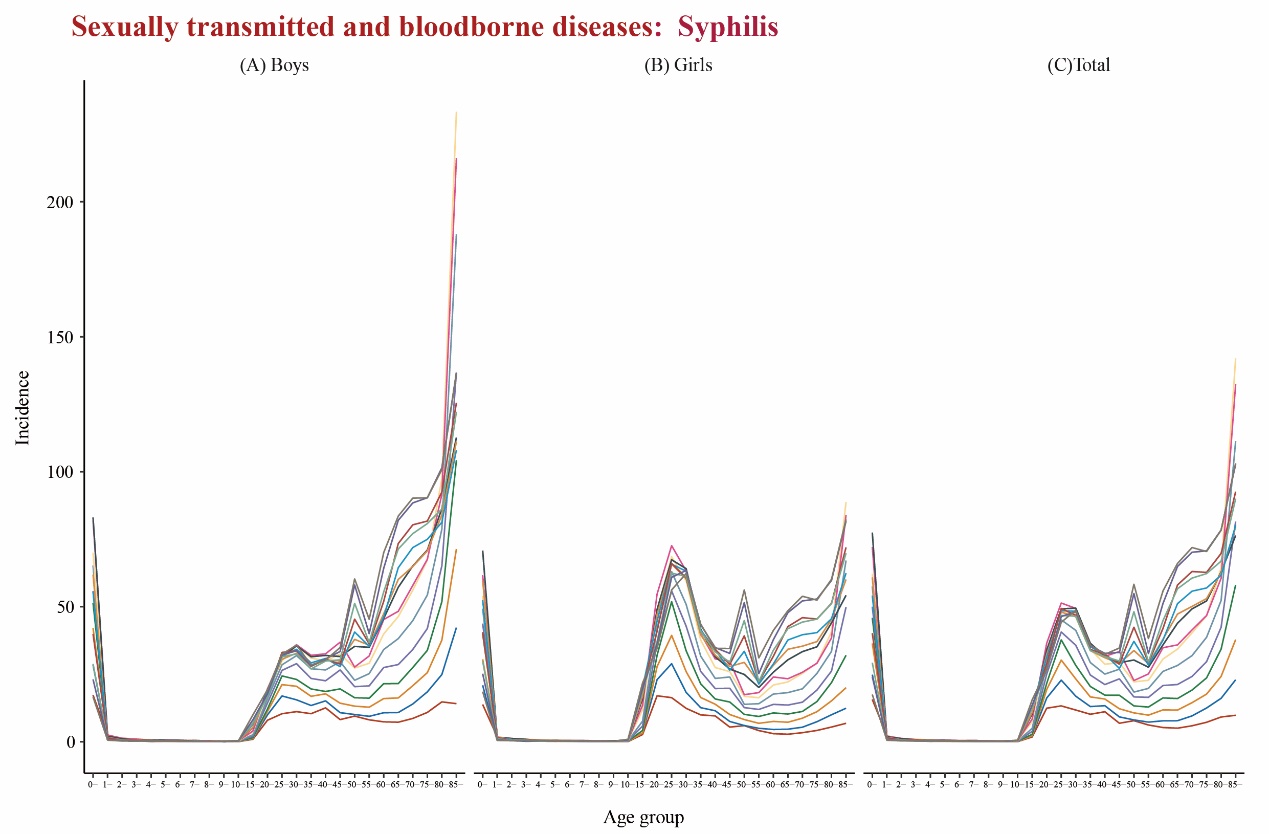


**Fig S5-42. Trends in age incidence rates for Syphilis, females and males, 2004-2018 (Legends were the same to Fig S5-1)**


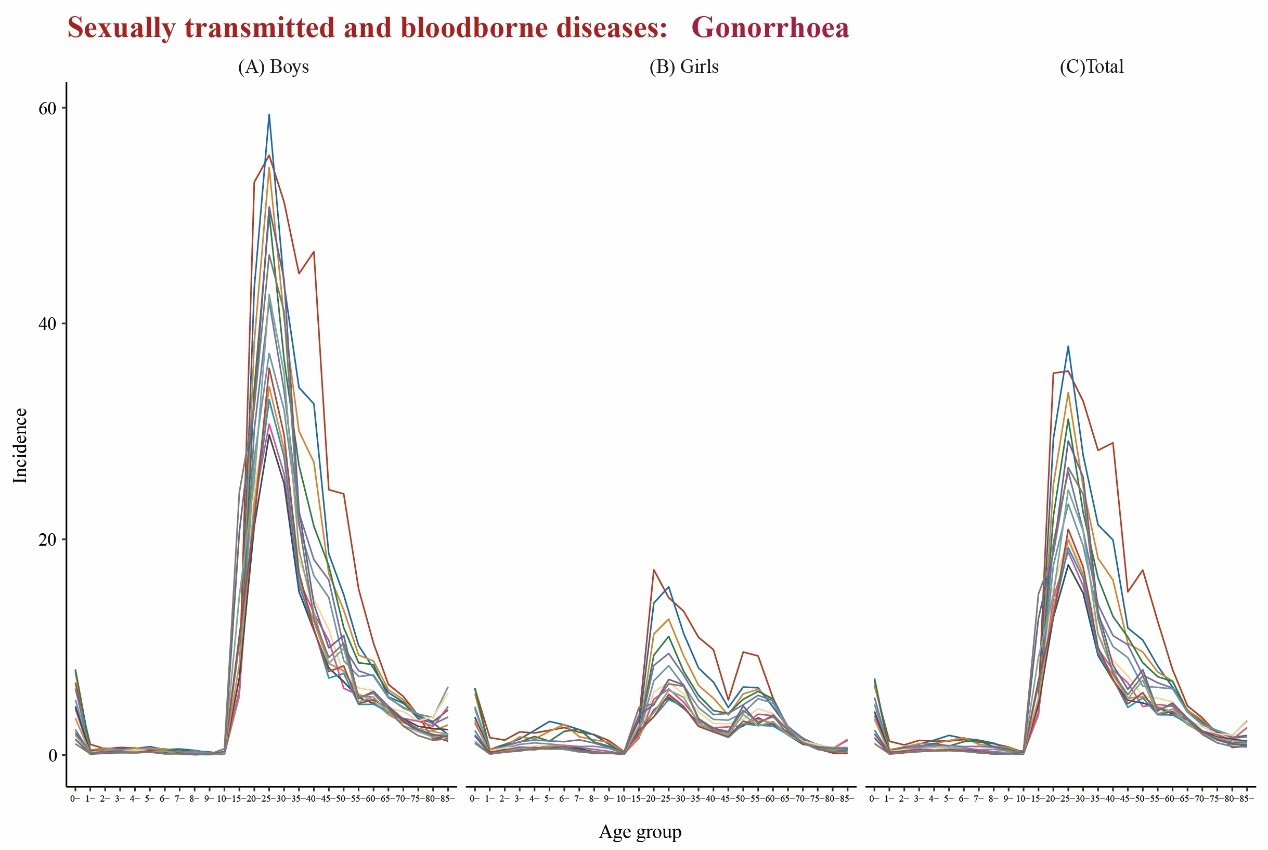


**Fig S5-43. Trends in age incidence rates for Gonorrhoea, females and males, 2004-2018 (Legends were the same to Fig S5-1)**


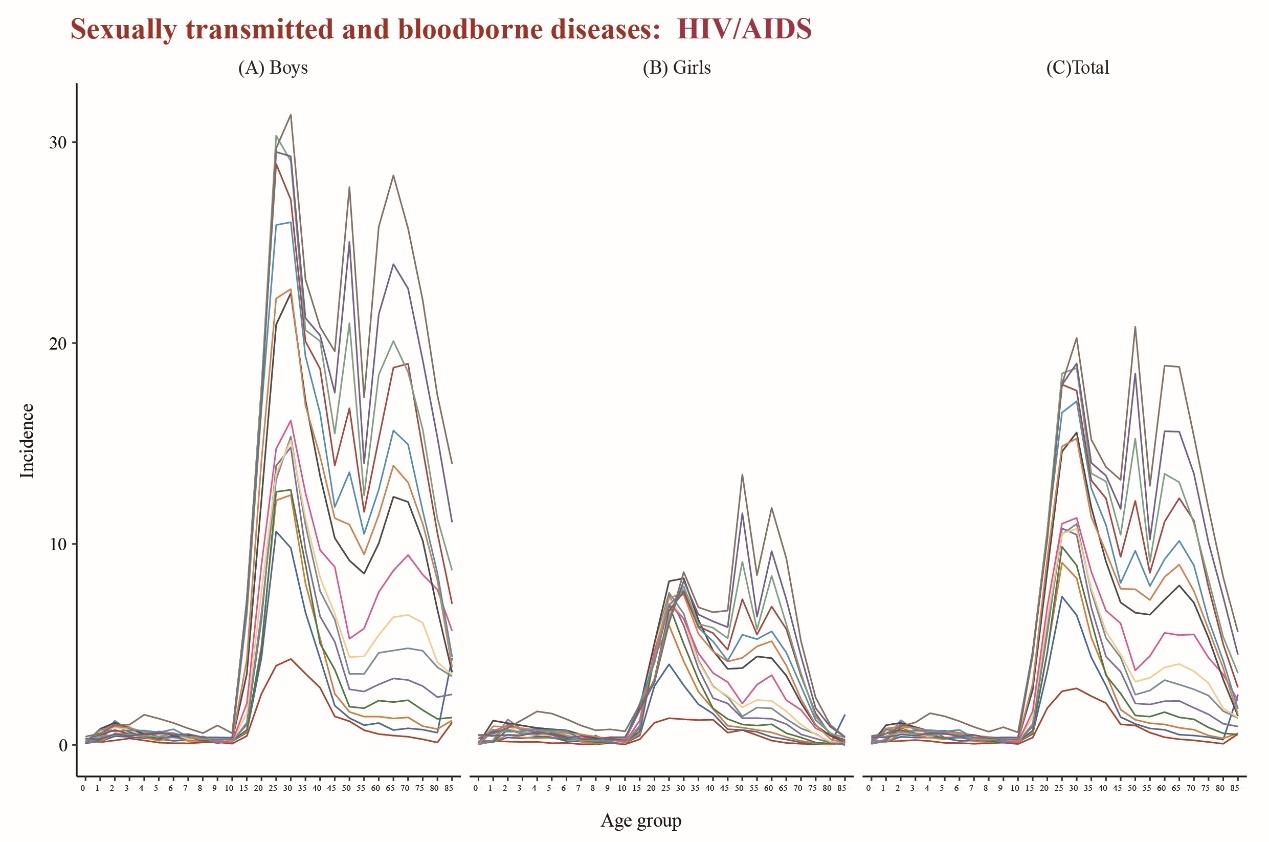


**Fig S5-44. Trends in age incidence rates for HIV/AIDS, females and males, 2004-2018 (Legends were the same to Fig S5-1)**

**Fig S6. The seasonal variation by month of seven categories of 44 notifiable infectious diseases from 2004 to 2018**


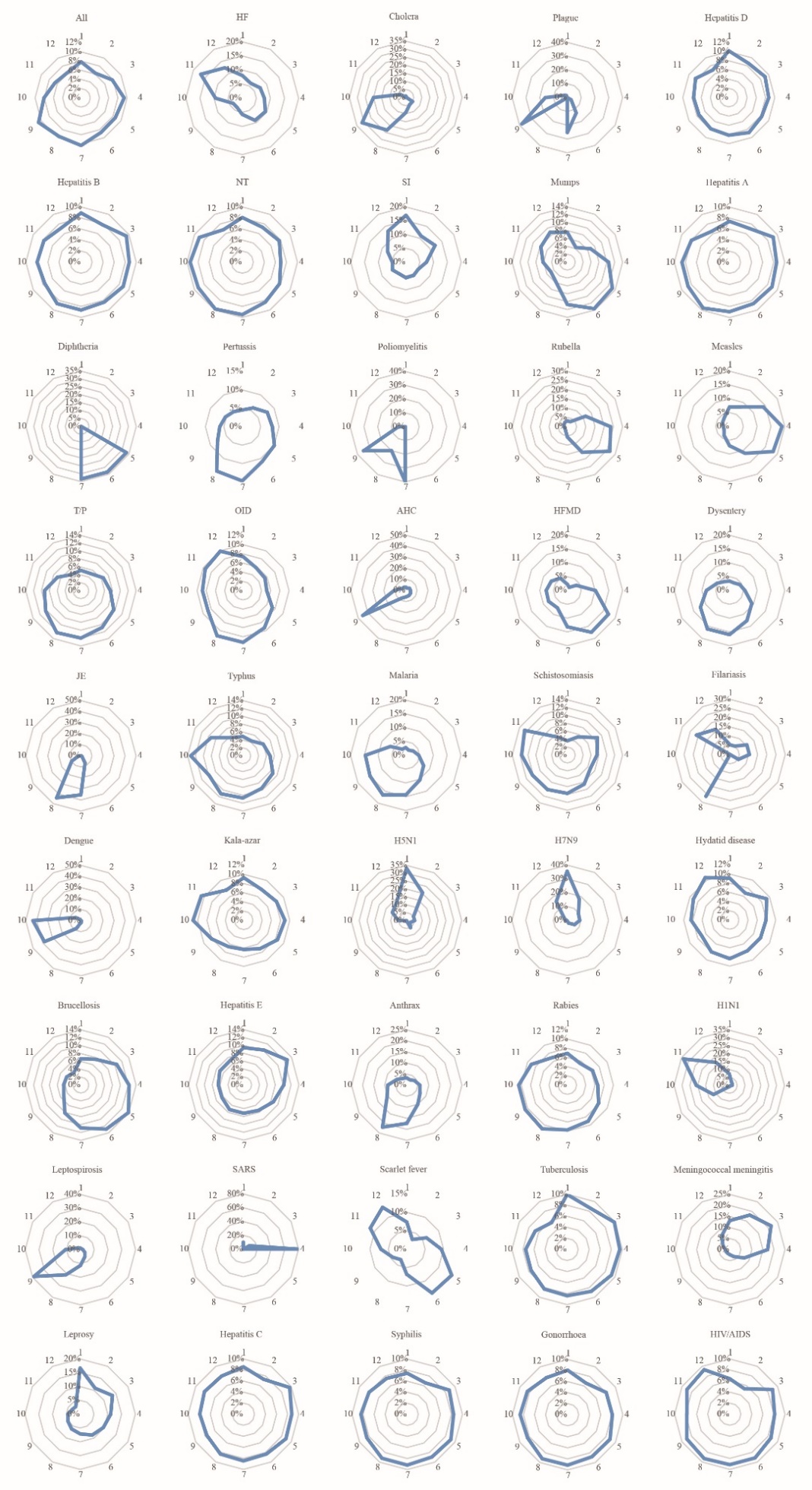


Note: In the radar diagram, the circumference represents 12 months clockwise, and the radius represents the incidence (per 100 000 people). HF = haemorrhagic fever. NT = neonatal tetanus. SI = seasonal influenza. T/P = typhoid/paratyphoid. OID = infectious diarrhoeal diseases other than cholera, bacterial and amoebic dysentery, typhoid, and paratyphoid. AHC = acute haemorrhagic conjunctivitis. HFMD = hand, foot, and mouth disease. JE = japanese encephalitis. SARS=severe acute respiratory syndrome.
